# The Leucine-mTOR-Autophagy Axis in Granulosa Cells Mediates Circadian Disruption-Induced Anovulation

**DOI:** 10.7150/ijbs.116803

**Published:** 2026-01-01

**Authors:** Renke He, Jiaying Mo, Zhongliang Lin, Kejing Zhu, Yishu Wang, Jiaen Yu, Haiyan Wu, Zhaoying Jiang, Qinyu Luo, Xueying Liu, Lin Yin, Chuanjin Yu, Jianzhong Sheng, Guolian Ding, Hefeng Huang

**Affiliations:** 1 Key Laboratory of Reproductive Genetics (Ministry of Education), Department of Reproductive Endocrinology, Women's Hospital, Zhejiang University School of Medicine, Hangzhou, China.; 2 Department of Obstetrics and Gynecology, Center for Reproductive Medicine, The Fourth Affiliated Hospital of School of Medicine and International School of Medicine, International Institutes of Medicine, Zhejiang University, Yiwu, China.; 3 Molecular Medicine Center, Guangzhou Women and Children's Medical Center, Guangzhou Medical University, Guangzhou, China.; 4 Assisted Reproduction Unit, Department of Obstetrics and Gynecology, Sir Run Shaw Hospital, Zhejiang University School of Medicine, Hangzhou, China.; 5 The International Peace Maternity and Child Health Hospital, School of Medicine, Shanghai Jiao Tong University, Shanghai, China.; 6 Department of Reproductive Endocrinology, The Second Affiliated Hospital Zhejiang University School of Medicine, Hangzhou, China.; 7 Obstetrics and Gynecology Hospital, Institute of Reproduction and Development, Fudan University, Shanghai, China.; 8 Research Units of Embryo Original Diseases, Chinese Academy of Medical Sciences, Shanghai, China.

**Keywords:** Continuous light, Ovulation disorders, BCAA, Autophagy, Mendelian randomization

## Abstract

**Background:** Ovulation disorders represent the most common cause of infertility in women. Previous studies have reported that continuous light exposure can induce anovulation. However, the underlying mechanisms remain unclear.

**Methods:** We assessed the phenotypes of ovulation disorders by using vaginal smears, hormone levels, and ovarian morphology. Metabolomics and RNA sequencing were employed to identify key metabolites and explore potential underlying mechanisms. Additionally, we investigated the effects of a leucine-rich diet on the phenotypes of ovulation disorders and autophagy. Serum levels of branched-chain amino acids (BCAAs) in patients with polycystic ovary syndrome (PCOS) were also measured. Causality was explored by using Mendelian randomization (MR) methods based on GWAS summary data.

**Results:** Female SD rats subjected to continuous light exhibited disrupted estrous cycles and polycystic ovaries, as well as increased anti-Müllerian hormone (AMH) levels. Metabolic profiling revealed that leucine was a pivotal metabolite. Specifically, a high-leucine diet induced anovulation and polycystic morphology, along with reducing autophagy, in rats under normal light conditions; additionally, leucine restriction alleviated these effects in recovered rats. Moreover, the mTOR-ULK1-LC3-II/I autophagy pathway was influenced both *in vivo* and *in vitro* by leucine. In patients with PCOS, elevated serum BCAA levels (especially leucine) were observed to be correlated with increased AMH levels, higher luteinizing hormone (LH)-to-follicle-stimulating hormone (FSH) ratios, and higher antral follicle counts. MR analysis indicated that night shift work may increase the risk of PCOS through elevated serum leucine levels.

**Conclusions:** These results suggest that the disruption of the leucine-mTOR-autophagy axis in granulosa cells (GCs) mediates continuous light-induced ovulation disorders. The potential therapeutic targeting of leucine-mTOR pathways for managing PCOS should be investigated.

## Introduction

The circadian rhythm, which is a natural 24-hour cycle, governs a range of physiological and behavioral processes in organisms, including sleep-wake patterns, thermoregulation, appetite, hormone secretion, and glucose homeostasis 1. However, contemporary lifestyle shifts, which are characterized by increased artificial light exposure, erratic eating habits, and reduced sleep duration, have led to a chronic misalignment between the body's internal circadian rhythm and the external environment 2. This misalignment has been implicated in an array of health risks, such as increased susceptibility to cancer, cardiovascular diseases, neuropsychiatric disorders, and metabolic dysfunctions 3-5.

Nighttime light exposure is particularly prevalent among shift workers and significantly affects reproductive health. This type of exposure has been associated with menstrual cycle irregularities, reduced fertility, and ovulation disorders, although the clinical evidence on this topic is inconsistent. These findings underscore the need to more thoroughly investigate the relationship between shift work and reproductive dysfunction 6, 7. Polycystic ovary syndrome (PCOS), which is the most common ovulation disorder among women of reproductive age, is associated with circadian rhythm disruptions 8-10. Studies have demonstrated that continuous light exposure can induce irregular estrous cycles and persistent anovulation in adult female rats, along with enlarged cystic follicles, which typically revert upon the cessation of light exposure 11. Although associations with neuroendocrine function 12, chronic stress 13, and the gut microbiota 10 have been explored in previous research, the precise mechanisms underlying these relationships remain unclear.

Healthy individuals exhibit rhythmic metabolic patterns 14, and disturbances in these patterns can lead to metabolic disorders, including glucose and lipid metabolism dysregulation, thereby increasing the risks of developing type 2 diabetes and obesity 4. The mammalian target of rapamycin (mTOR) pathway is a key regulator of cellular activity and is implicated in the development of ovulation disorders. The modulation of mTOR activity via nutritional status, drug concentrations, and hormone levels can regulate the apoptosis, autophagy, and proliferation of ovarian granulosa cells (GCs) 15. However, the relationship between circadian rhythm disturbances and the mTOR-autophagy pathway remains unexplored. PCOS is characterized by ovulation disorders, hyperandrogenism, obesity, insulin resistance, and other metabolic abnormalities. These features underscore the interplay between circadian rhythm disruptions, metabolic dysfunction, and impaired ovulation. GCs, which constitute more than 50% of the ovarian cell population, play a central role in folliculogenesis, steroidogenesis, and oocyte support. Importantly, GC dysfunction has been strongly implicated in the pathogenesis of key PCOS traits 16, 17.

An understanding of the molecular mechanisms through which continuous light exposure influences metabolism and leads to ovulation disorders is of clinical importance. Moreover, the investigation of intervention strategies that modify unhealthy lifestyles and dietary habits could offer new approaches for treating ovulation disorders. Our previous research revealed that continuous light exposure can disrupt normal ovulation 18. Based on these findings, we aimed to elucidate the mechanisms by which metabolites mediate these effects through the mTOR signaling pathway. Therefore, we employed an integrative approach that combines metabolomics and transcriptomics analyses to identify a candidate key metabolite (leucine). We also utilized light recovery models and leucine feeding experiments to simulate the potential benefits of lifestyle changes on ovulation disorders. Clinical samples were analyzed to determine serum leucine levels and their correlations with clinical manifestations. Finally, we conducted a two-sample, two-step, bidirectional Mendelian randomization analysis based on genome-wide association study (GWAS) data for validation. This research addresses a significant gap in the literature and is crucial for developing lifestyle intervention strategies to manage ovulation disorders associated with circadian rhythm disruptions, thereby ultimately promoting reproductive health and fertility in women of childbearing age.

## Methods

### Rats

The rats in the normal light (NL) group were housed in SPF facilities with a 12 h:12 h light:dark cycle. In contrast, the continuous light (CL) group included 3-week-old female SD rats housed under the same conditions but exposed to continuous light for 24 hours. The continuous light-normal light (CN) group received 24 h of light exposure for 8 weeks, followed by a transition to a 12 h light:dark cycle for 4 weeks. This research was conducted with the approval of the Experimental Animal Ethics Committee of Zhejiang University (No. ZJU20220383).

To evaluate the effect of dietary leucine on the phenotype of the model rats, we established an additional three groups based on the previously defined groups, with each group receiving varying levels of leucine. The NL group was provided with a high-leucine diet (NL+H-leu), whereas the CL group received a low-leucine diet (CL+L-leu), beginning from 3 weeks of age until 15 weeks. The CN+L-leu group was exclusively fed a low-leucine diet during the recovery period. The high-leucine diet contained twice the standard amount of leucine, whereas the low-leucine diet contained one-fifth of the standard amount 19. All of the phenotypes of the female rats were analyzed after either 12 weeks or 4 weeks of dietary intervention.

### Sample preparation

Weekly body weight measurements were obtained between 3 and 15 weeks of age. To examine the role of the circadian clock, rats from each group were euthanized at 4-hour intervals across a 24-hour cycle (ZT0/24, ZT4, ZT8, ZT12, ZT16, and ZT20). Zeitgeber time (ZT) was standardized based on the light:dark cycle in the animal facility, with ZT0 (lights-on) set at 7:00 AM and ZT12 (lights-off) set at 7:00 PM. Vaginal cytology confirmed the estrous stage before sample collection. The adrenal glands, liver, ovaries, serum, and GCs were harvested after euthanasia for experiments and analysis. Moreover, the ovarian index was calculated as the ovarian weight (mg)/body weight (g).

### Isolation and culture of granulosa cells

In accordance with our previously published methods 18, GCs were isolated from 3-week-old female rats following priming with 10 IU of pregnant mare serum gonadotropin (PMSG). After 48 hours, GCs were collected via follicular puncture and subsequently purified via sequential centrifugation and resuspension steps. The isolated cells were cultured at a density of 5×10⁵ cells/mL in high-glucose DMEM (Vivacell, C3101-0500) supplemented with 10% fetal bovine serum (Biological Industries, 04-001-1A) and antibiotics. Through a comprehensive literature review, as well as time-course and dose-response analyses, we determined that treatment with L-leucine (Sigma, L8912) at 3× the physiological concentration for 2 hours and treatment with an autophagy inducer (MCE, HY-119137) at 10 μM for 2 hours yielded optimal results for our experimental objectives 20, 21.

### Quantitative real-time PCR (qPCR)

Total RNA was extracted with an RNAfast 200 Kit (Fastagen, 220011), and cDNA was synthesized via the PrimeScript™ RT Reagent (Perfect Real Time) Kit (TaKaRa, RR047A). Quantitative PCR (qPCR) analysis was conducted with a SYBR Premix Ex Taq (Tli RNaseH Plus) system (TaKaRa, RR420A) on a LightCycler 480 II instrument (Roche, Switzerland). All of the procedures were performed according to the manufacturer's instructions. The differential gene expression results between the samples (ΔΔ Ct) were calculated as the fold difference (2-ΔΔ Ct). The primer sequences for the tested genes are provided in the Supplementary material.

### Western blot

The GC samples were homogenized in RIPA buffer (CST, 9806) containing a protease inhibitor cocktail (CST, 5872). The protein per sample was loaded and separated on a 4-20% SDS-PAGE gel. The separated samples were transferred to nitrocellulose membranes (Pall, P-N66485). After blocking with 5% BSA in TBST, the membranes were incubated with primary antibodies against p-mTOR (Ser2448) (CST, 2971, 1:1000), T-mTOR (CST, 4517, 1:1000), p-ULK1 (CST, 14202, 1:1000), ULK1 (MCE, HY-P80366, 1:1000), p62/SQSTM1 (ABclonal, A19700, 1:5000), LC3 (Sigma, L7543, 1:1000), p-PARP (CST, 94885, 1:1000), Caspase3 (Proteintech, 19677-1-AP, 1:1000), Caspase6 (Proteintech, 10198-1-AP, 1:1000), Gapdh (CST, 2118, 1:10000), Actin (CST, 4790, 1:1000), and Vinculin (CST, 13901, 1:1000) at 4 °C overnight. Following incubation with the corresponding secondary antibodies (LI-COR, 926-32211, 926-68071), the results were obtained with an Odyssey CLx system (LI-COR, USA) and analyzed with Image Studio software.

### Flow cytometric analysis of apoptosis

The viability and apoptotic status of the GCs were assessed via flow cytometry (Beckman Coulter Life Sciences) by using dual staining with annexin V and MitoTracker. Apoptosis was detected with a commercial apoptosis detection kit (C1071; Beyotime Biotechnology, China) in accordance with the manufacturer's instructions. The early phase of apoptosis was defined as annexin V+/Mito+, whereas the late phase of apoptosis was defined as annexin V+/Mito-.

### Serum untargeted metabolomics sequencing

Following sample extraction, the samples were separated via the Nexera UHPLC LC-30A liquid system in both positive (POS) and negative (NEG) modes, after which they were analyzed on a Q Exactive HF-X mass spectrometer. The raw data from the mass spectrometry analysis were processed via Progenesis QI software for database retrieval to eventually obtain identification information of the sample. Bioinformatics analysis, including cluster analysis, orthogonal partial least-squares discriminant analysis (OPLS-DA), and KEGG pathway annotation and enrichment, was also performed.

### GC-targeted amino acid metabolomics

The standard configuration was prepared, and the metabolites were extracted from the GCs according to the manufacturer's instructions. Afterward, an ACQUITY UPLC^®^ BEH C18 chromatographic column (2.1x100 mm, 17 μm; Waters, USA) was used, and multiple reaction monitoring (MRM) was used for the scan analysis.

### RNA sequencing

The GCs were harvested from three rats in each group. RNA extraction was performed via the TRIzol method according to the manufacturer's instructions. The RNA sequence was provided by Shenzhen Genome Institute (BIG, China) and subsequently quantified with an Agilent 2100 Bioanalyzer and ABI StepOnePlus Real-Time PCR System. The library was subsequently sequenced on an Illumina HiSeq 2000 system. DEGs were identified based on an adjusted p value ≤ 0.05 and a fold change ≥ 1.5 by using Cuffdiff. Venn analysis was performed, and heatmaps were generated online at https://biosys.bgi.com. KEGG, GO and GSEA analyses, as well as visualization, were performed by using the online platform OmicShare tools (https://www.omicshare.com/tools/).

### Statistical analysis

All of the experiments were independently repeated at least three times. Statistical analyses were performed by using SPSS (version 25.0) and GraphPad Prism (version 10.0). For comparisons between two groups, an unpaired Student's t test was used, whereas ANOVA was used for multiple-group analyses, followed by either Dunnett's test, Tukey's multiple test or Sidak's multiple comparisons test. For comparisons of nonparametric data, the Mann-Whitney test was used. The data distribution was assumed to be normal; however, this was not formally tested. The data are expressed as the mean±SEM. Pearson correlation analysis was conducted to evaluate the relationships between BCAA levels and clinical indicators. All of the tests were two-sided, with statistical significance set at p < 0.05.

### Study population

#### Subjects

Human serum samples were collected from the International Peace Maternity and Child Health Hospital, School of Medicine, Shanghai Jiao Tong University. The study received approval from the Ethics Committee with all the participants providing written informed consent. In total, 45 participants (including women undergoing *in vitro* fertilization treatment) were enrolled. Among them, 29 participants were diagnosed with PCOS, whereas 16 age-matched and BMI-matched infertile women with tubal blockage served as controls. The antagonist protocol for controlled ovarian hyperstimulation (COH) was performed according to previously established methods. Specific exclusion criteria and additional information are provided in the Supplementary materials.

#### Data source

The dataset of the PCOS patients was obtained from the GWAS cohort in Finland and included 31,548 cases and 179,322 controls. The summary dataset for female reproductive hormones was acquired from the UK Biobank (European ancestry, TT levels, n=230,454; Bio-T levels, n = 188507; SHBG levels, n= 189,473; E_2_ levels, n=163,985) 22, 23. AMH summary statistics originated from a GWAS meta-analysis including five queues and 3,344 premenopausal women 24. The dataset of serum BCAA levels was retrieved from the public GWAS database known as IEU open GWAS (https://gwas.mrcieu.ac.uk/), which included 115,048 participants with BCAA and valine (Val) levels, as well as 115,047 participants with isoleucine (Ile) and leucine (Leu) levels 25 (Supplementary Table 2).

#### Selection of instrumental variables

The SNPs employed as instrumental variables (IVs) were obligated to have achieved genome-wide significance, with *P* values < 5.00E-8 or 5.00E-6. Additionally, we performed linkage disequilibrium (LD) detection, which was conducted to guarantee the independence of the SNPs and eliminate the SNPs of LD 26. The conventional parameters were set as R^2^ = 0.001 and KB = 10000 (Supplementary Table 3-8).

#### Statistical analysis

Two-sample MR analysis (TSMR) was conducted with R software (version 4.2) via the “TwoSampleMR” package. The results were divided into four types, including inverse variance weighting (IVW), the MR-Egger method (MRE), the weighted median (WM), and the weighted model (wm), to increase the reliability of the results 27. The IVW model was selected as the primary analysis method because it is the most effective and widely utilized model. When the calculation results of the various abovementioned methods were in the same direction and the *P* value was < 0.05, causal inference was suggested 28. MR-PRESSO (MRE and Mendelian randomization pleiotropy residual sum and outlier) analysis was employed to detect outliers and eliminate deviations caused by horizontal pleiotropy. After the removal of abnormal SNPs, correction results were obtained in the MR-PRESSO analysis 29. The heterogeneity level was quantified by using the Cochran's Q test and the I^2^ statistic, and significant heterogeneity was determined by a *P* value < 0.05. Additionally, the “leave-one-out” approach was utilized for sensitivity analysis to determine when the exclusion of any single SNP would lead to the disappearance of the causal effect 30.

### Multivariate MR analysis

Multivariable Mendelian randomization (MVMR) was implemented after adjusting for the major determinants of exposure and outcome to minimize bias. The IVW approach regards multiple risk factors as exposures to simultaneously estimate their genetic predictive influence on the outcome via the MVMR package 31. All of the results are presented in the resulting plot, forest plot, scatter plot, leave-one-out plot, or funnel plot. A *P* value < 0.05 was considered to indicate statistical significance. However, in the repeated test, the *P* value of threshold correction was determined via Bonferroni correction (*P* value < 0.05/n, n = the number of exposures × the number of outcomes). When the outcome variable was a continuous variable, the results are presented as β values (95% CI). When the outcome was a binary classification variable, the results are presented as odds ratios (ORs) with corresponding 95% CI values.

## Results

### Continuous light exposure interferes with the reproductive cycle

**Figure 1:**
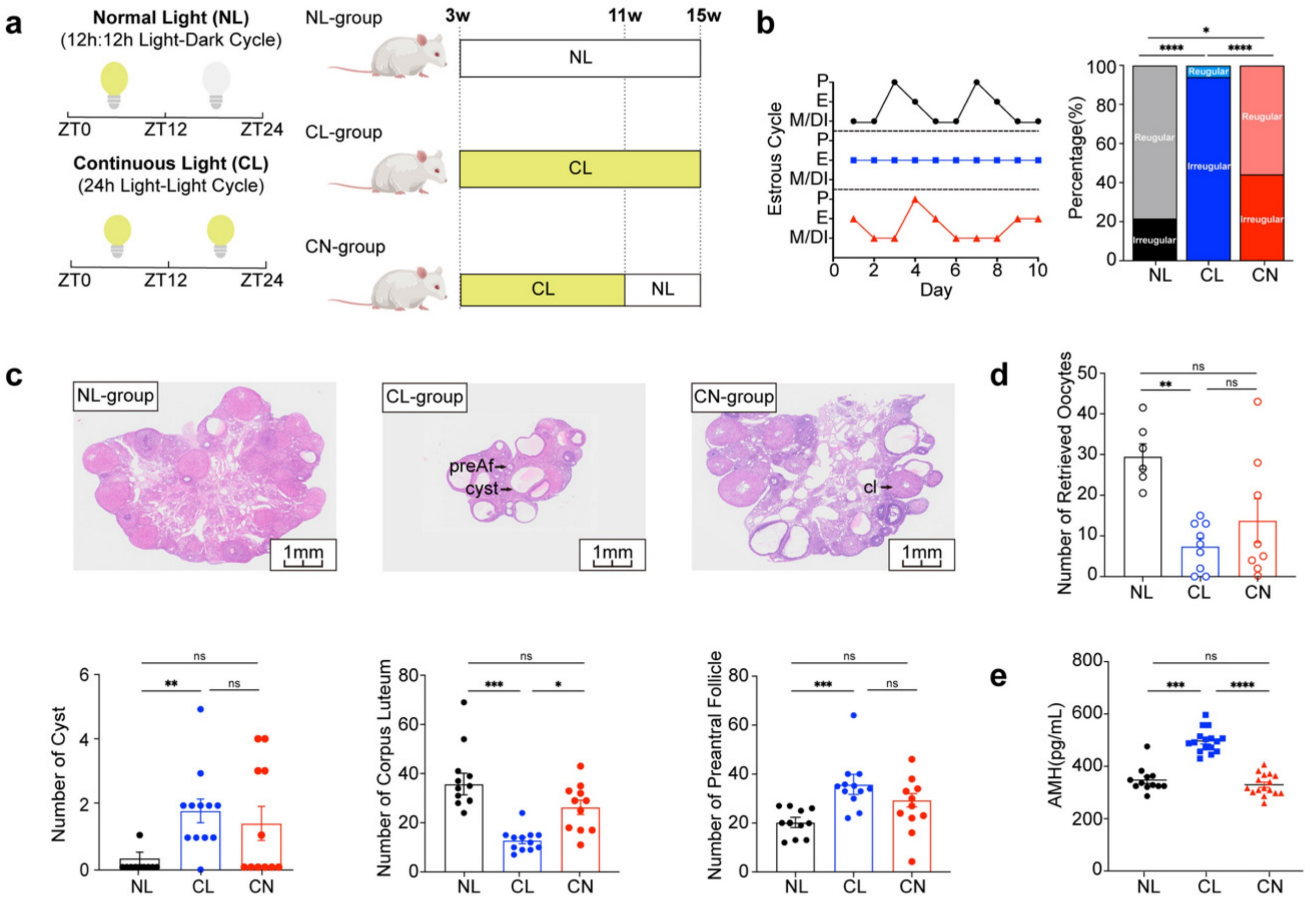
** Continuous light exposure interferes with the reproductive cycle. (a)** Schematic diagram illustrating the construction of a rat model with continuous and reinstated light illumination. Zeitgeber time (ZT): ZT0 (7:00 AM) or ZT12 (7:00 PM). **(b)** Estrus cycles were recorded for 10 continuous days. The panels display the normal light (NL), continuous light (CL), and continuous-normal light (CN) groups, which are indicated in black, blue, and red colors, respectively. P (proestrus); E (estrus); M (metestrus); D (diestrus) (Left). The bar chart shows the percentage of irregular estrous cycles in the three groups of rats (right). **(c)** Histological anatomy and microscopic ovarian structures are shown in panels from left to right and represent the NL, CL, and CN groups. The arrow indicates preantral follicles (preAf), cysts (cyst), and the corpus luteum (cl). The bar chart shows the numbers of preAf, cysts, and corpus lutea in all of the groups of rats (n = 11, 12, and 11 for the NL, CL and CN groups, respectively).** (d)** Number and statistical analysis of oocytes recruited after PMSG-hCG ovulation induction (n = 6, 9, and 8 for the NL, CL and CN groups, respectively). **(e)** Serum levels of AMH were assessed via ELISA (n = 12, 16, and 16 for the NL, CL and CN groups, respectively). The data are presented as the mean ± SEM. Statistical analysis was performed via one-way ANOVA with Tukey's multiple comparison post hoc test. *P < 0.05; **P < 0.01; ***P < 0.001; ****P < 0.0001.

Three-week-old female SD rats were randomly divided into three groups (Fig. 1a). The normal light (NL) group was maintained under a 12-hour light:dark cycle, with lights on at 7:00 AM (designated ZT0) and lights off at 7:00 PM (designated ZT12), with a light intensity of 600 lux being utilized. The continuous light (CL) group was exposed to constant light at an intensity of 600 lux for 24 hours per day over a duration of 12 weeks. The continuous-normal light (CN) group was exposed to continuous light for the first 8 weeks and then to a normal light cycle for 4 weeks.

We evaluated the reproductive phenotypes of the three groups. The typical estrous cycle involves four phases, including the proestrus (P), estrus (E), metestrus (M), and diestrus (DI) phases, which occur for 4-5 days (Supplementary Fig. 1a). Continuous exposure to light disrupted this cycle, thus resulting in a prolonged E phase. However, upon returning to a normal light:dark cycle, the estrous cycle in the CN group exhibited signs of recovery. The incidence rates of irregular estrous cycles among the three groups were approximately 20%, 90% and 40%, respectively (Fig. 1b). Chronic disruption of circadian rhythms resulted in polycystic ovaries and ovulation disorders, as indicated by a reduced ovarian-to-body weight ratio, lower corpus luteum counts, and increases in the numbers of cysts and preantral follicles (Fig. 1c and Supplementary Fig. 1b and c). Moreover, circadian disruption adversely affected the number of retrieved oocytes; however, some improvement was noted after adjustments were made to the lighting conditions (Fig. 1d and Supplementary Fig. 1d). Notably, the serum levels of anti-Müllerian hormone (AMH) were significantly elevated in the CL group (Fig. 1e), whereas no significant differences were detected in the serum levels of luteinizing hormone (LH), follicle-stimulating hormone (FSH), estrogen (E_2_), or total testosterone (TT) (Supplementary Fig. 1e).

Additionally, we performed a comprehensive analysis of fasting blood glucose levels, lipid levels, serum insulin levels, body composition, glucose tolerance tests (GTTs), and insulin tolerance tests (ITTs) across the NL, CL, and CN groups. Our findings revealed no statistically significant differences among these indicators (Supplementary Fig. 2a-j).

### Continuous light exposure leads to dysregulation of serum metabolite rhythms

**Figure 2:**
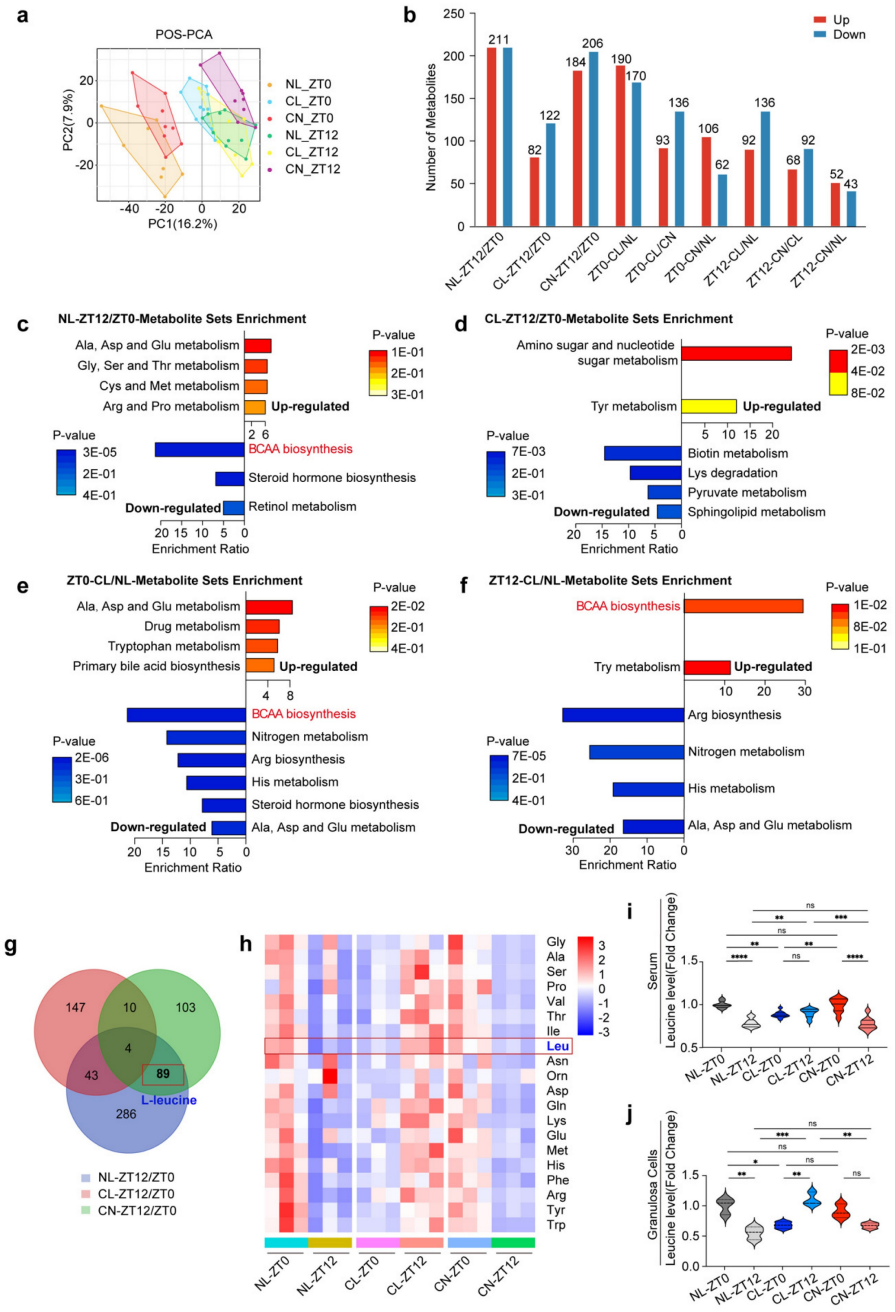
** Continuous light exposure leads to dysregulation of serum metabolite rhythms. (a)** PCA score plots derived from LC-MS data of serum samples at different timepoints (ZT0 and ZT12) in three groups of rats. Panels show the PCA score in POS mode (n = 8, 10, and 9 for the NL, CL and CN groups, respectively, at ZT0; n = 10, 8, and 9 for the NL, CL and CN groups, respectively, at ZT12). **(b)** Number of differentially abundant metabolites in all of the comparisons, including circadian changes in the NL, CL, and CN groups, as well as the differences at ZT0/ZT12 in the three groups; upregulated metabolites are represented by red bars, whereas downregulated metabolites are represented by blue bars.** (c-d)** Top enriched KEGG pathways associated with differentially abundant metabolites in the NL and CL groups at ZT12/ZT0. **(e-f)** Top KEGG enrichment pathways enriched with differentially abundant metabolites when comparing CL/NL at ZT0 and ZT12. **(g)** Venn diagram overlapping ZT12/ZT0 in the NL, CL and CN groups. **(h)** Heatmap showing the amino acid changes in 20 types of GCs in the three groups at ZT0 and ZT12 (n = 3 for all groups). **(i-j)** Violin plots showing the changes in leucine levels in both the serum and GCs. The data are presented as the mean ± SEM. Statistical analysis was performed via two-way ANOVA with Sidak's multiple comparisons. *P < 0.05; **P < 0.01; ***P < 0.001; ****P < 0.0001.

We subsequently examined the overall serum metabolic changes across the NL, CL, and CN groups. In both POS and NEG mode, the histograms revealed a significant number of different metabolites identified by comparing two timepoints (ZT0 vs. ZT12) within the same group and by analyzing different groups at the same timepoint (Fig. 2a and b and Supplementary Fig. 3a).

We first conducted comparisons between two timepoints (ZT0 and ZT12) for each group and identified the most significantly enriched pathways. Our findings revealed that the metabolites exhibiting circadian patterns in the NL group were predominantly enriched in multiple metabolic pathways. Notably, a series of amino acid pathways (including alanine, cysteine, methionine, aspartic acid and proline) were upregulated, whereas the biosynthesis of steroid hormones, retinol metabolism, and BCAA biosynthesis and degradation were downregulated (Fig. 2c). Importantly, the diurnal rhythm pattern of BCAA metabolism in the CL group at ZT0 and ZT12 was lost (Fig. 2d). In the CN group, the BCAA metabolic pattern recovered, which was consistent with that in the NL group (Supplementary Fig. 3b).

Furthermore, we compared the NL and CL groups at the same timepoint. At ZT0, the spectrum of differentially abundant metabolite enrichment pathways was broader in the CL group than in the NL group. This included the upregulation of alanine, aspartic acid and glutamic acid metabolism; tryptophan metabolism; and primary bile acid biosynthesis, along with the downregulation of pathways related to BCAA biosynthesis and degradation; nitrogen metabolism; arginine biosynthesis; and histone metabolism (Fig. 2e). At ZT12, the BCAA biosynthesis pathway was upregulated in the CL group (Fig. 2f).

### The rhythmic patterns of leucine in the serum and granulosa cells are disrupted by continuous light exposure

We obtained a thorough understanding of serum metabolite fluctuations and subsequently compared the differentially abundant metabolites across the NL, CL, and CN groups at ZT0 and ZT12. A Venn diagram revealed that 89 metabolites exhibit circadian rhythms under normal light conditions; these rhythms disappear under continuous light conditions, and they can recover after a return to normal light conditions (Fig. 2g). Further analysis via KEGG pathway annotation and enrichment revealed that these metabolites were primarily associated with BCAA biosynthesis, steroid hormone biosynthesis, BCAA degradation and terpenoid backbone biosynthesis (Supplementary Fig. 3c).

Based on these results, we specifically focused on the pathways associated with serum amino acid metabolism. We normalized the relative values of each amino acid to the mean of the NL group at ZT0 and performed statistical analyses. Violin plots and heatmaps were constructed to illustrate both the classical and significantly altered amino acids (Supplementary Fig. 3d and Supplementary Fig. 4a-l). Similar visualizations were applied to represent the levels of these amino acids in GCs (Fig. 2h and Supplementary Fig. 5a-j). Interestingly, our metabolic sequencing of both the serum and GCs consistently revealed rhythmic patterns for leucine, with significant variations being observed. Therefore, leucine was identified as a potential differentially abundant metabolite worthy of further exploration (Fig. 2i and j).

### The control of dietary intake of leucine can regulate ovulation in female rats

**Figure 3:**
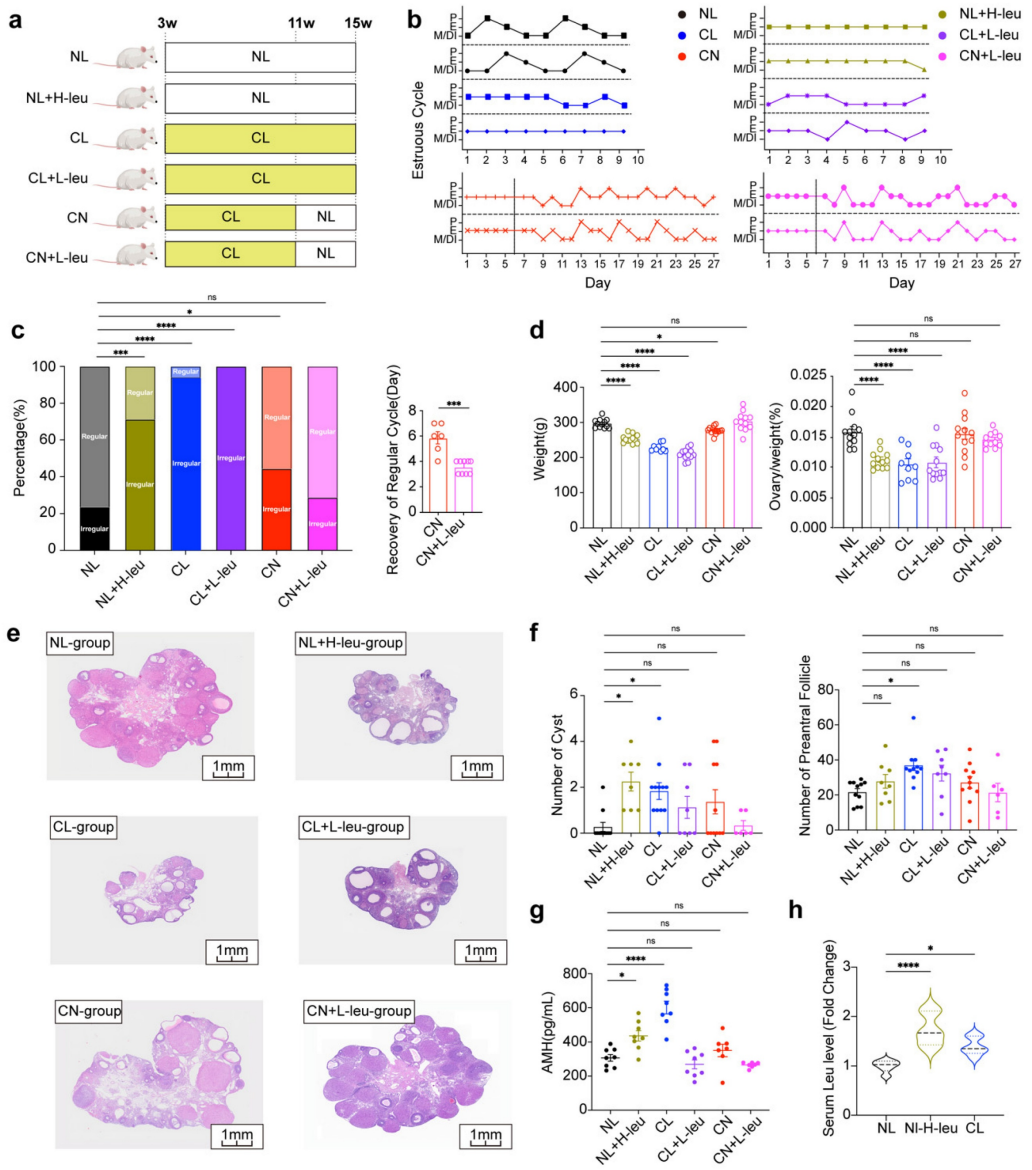
** The control of dietary intake of leucine can regulate ovulation in female rats. (a)** Schematic diagram illustrating the construction of a rat model with a leucine diet and circadian disturbance, including NL, NL combined with a high-leucine diet (NL+H-leu), CL, CL combined with a low-leucine diet (CL+L-leu), CN, and CN combined with a low-leucine diet (CN+L-leu). Details are provided in the diagram. **(b)** Estrus cycles were monitored for the preceding month. The panels show the NL (n = 13), NL+H-leu (n = 13), CL (n = 13), CL+L-leu (n = 13), CN (n = 14), and CN+L-leu (n = 14) groups, which are colored black, olive green, blue, purple, red and pink, respectively. **(c)** Bar chart showing the percentage of irregular estrous cycles in each group of rats (left) and the days of recovery from the irregular cycle between the CN (n = 6) and CN+L-leu groups (n = 9). **(d)** Comparisons of the weights and ovary indices of the NL (n = 12), NL+H-leu (n = 12), CL (n = 9), CL+L-leu (n = 12), CN (n = 12), and CN+L-leu (n = 12) groups. **(e)** Histological microscopy images of the ovarian structures of all of the groups are shown. **(f)** Bar chart showing the analysis of the numbers of cysts and preAFs in the NL (n = 11), NL+H-leu (n = 8), CL (n = 11), CL+L-leu (n = 8), CN (n = 11), and CN+L-leu (n = 6) groups. **(g)** Serum levels of AMH were measured via ELISA in the NL (n = 8), NL+H-leu (n = 8), CL (n = 8), CL+L-leu (n = 8), CN (n = 6), and CN+L-leu (n = 7) groups. **(h)** Bar chart showing the serum leucine levels in the NL, NL+H-leu and CL groups of rats at ZT12 (n = 6). The data are presented as the mean ± SEM. Statistical analysis was performed via one-way ANOVA with Dunnett's test and an unpaired two-sided Student's t test. *P < 0.05; **P < 0.01; ***P < 0.001; ****P < 0.0001.

Lifestyle interventions, including exercise and diet, have consistently been considered as primary treatments for women with PCOS 32. To further assess the impact of leucine on ovulation, we examined various experimental groups (Fig. 3a). The estrous cycle of the NL group was observed to be regular, whereas those of the NL with high leucine intake (NL+H-leu), CL, and CL with low leucine intake (CL+L-leu) groups were disrupted (Fig. 3b). The rates of recovery in both the CN and CL with low leucine intake (CN+L-leu) groups were similar to those of a regular estrous cycle; however, the recovery time of the CN+L-leu group was significantly faster (Fig. 3c).

Compared with those of the NL group, the body weights and ovarian indices of the NL+H-leu group were significantly lower. No significant differences were observed between the CL and CL+L-leu groups or between the CN and CN+L-leu groups (Fig. 3d). Furthermore, changes in ovarian morphology were more pronounced in the NL+H-leu, CL, and CL+L-leu groups than in the other groups. Our findings indicated that the ovaries exhibited polycystic characteristics, with multiple thin-GC follicles being detected in the three abovementioned groups. These findings suggest that high leucine intake can mimic the effects of continuous light exposure on these parameters, whereas treatment with a low-leucine diet alone cannot reverse this phenotype. In contrast, the NL, CN, and CN+L-leu groups maintained normal ovarian morphology, with no apparent large follicles being detected. Notably, although the changes in the CN+L-leu group did not achieve statistical significance compared with those in the CN group, we also observed a clear decreasing trend in the numbers of cysts and preAFs, which suggests that light recovery combined with a low-leucine diet may have a better restorative effect on these phenotypes (Fig. 3e-f). Moreover, compared with the NL+H-leu and NL groups, a high-leucine diet may promote the secretion of AMH. Additionally, AMH levels were significantly reduced by a lower leucine diet, as observed in the CL+L-leu and CL groups (Fig. 3g). All of the observed phenotypes may have been caused by abnormal serum leucine levels at ZT12; furthermore, compared with the NL group, AMH levels in both the NL with high leucine intake (NL+H-leu) group and the CL group were significantly greater (Fig. 3h).

### The impact of continuous light exposure on the gene expression rhythm in GCs

**Figure 4:**
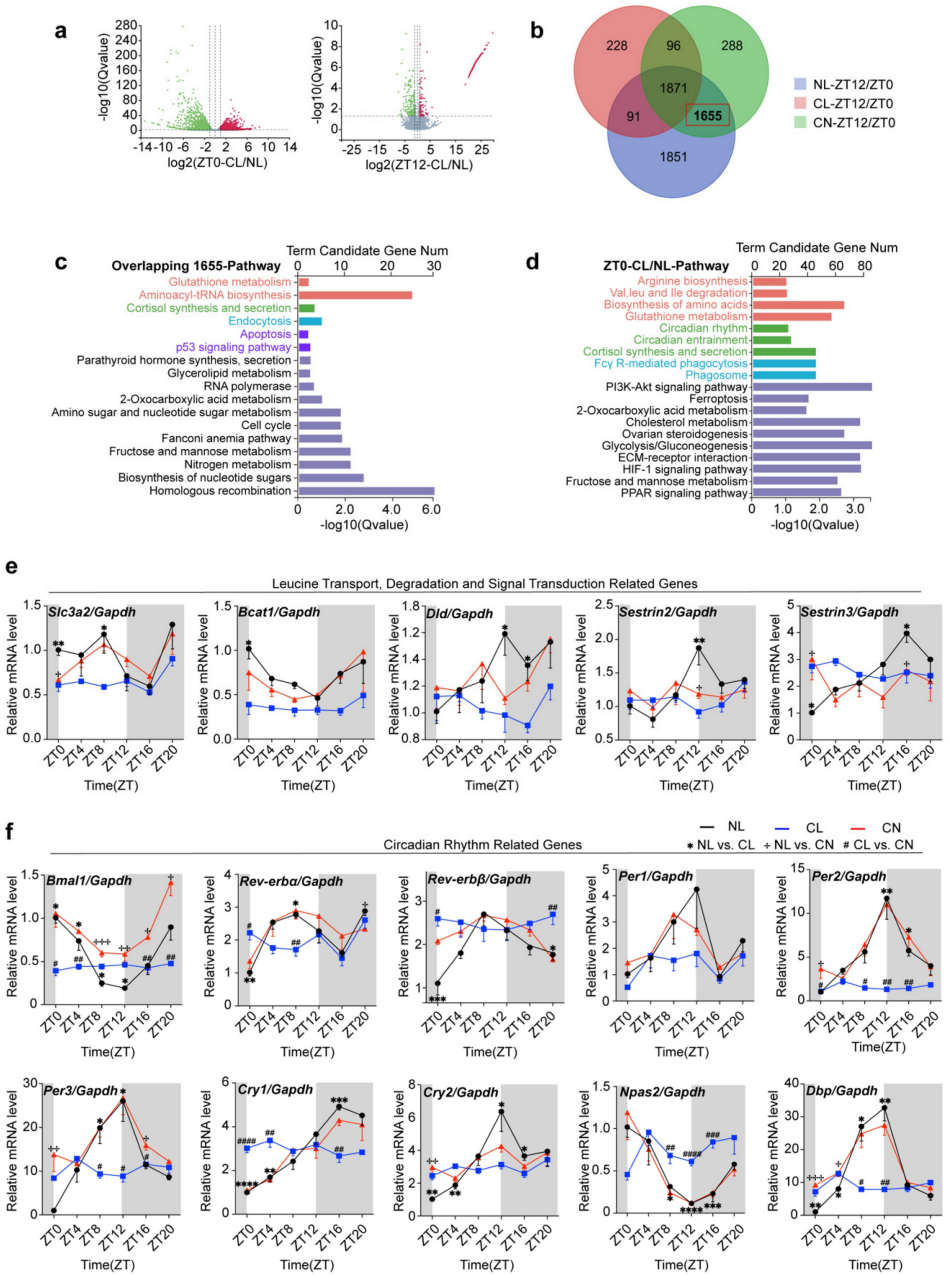
** The impact of continuous light exposure on the gene expression rhythm in GCs. (a)** Volcano plots showing the genes that were differentially expressed between the NL and CL groups at ZT0 and ZT12. **(b)** Venn diagram illustrating the overlap of differentially expressed genes from ZT12/ZT0 in the three groups. In total, 1655 DEGs were identified as being influenced by continuous light exposure but were rescued by normal light exposure.** (c)** Enriched KEGG and GO analyses of 1655 genes. **(d)** Top KEGG and GO pathway enrichment analyses of differentially expressed genes comparing CL/NL at ZT0. **(e)** Circadian expression patterns of genes related to leucine transport, degradation, and signal transduction from ZT0 to ZT20 (n = 4 for all groups and timepoints). **(f)** Circadian expression patterns of core clock genes (including Bmal1, Rev-erbα, Rev-erbβ, Per1, Per2, Per3, Cry1, and Cry2) from ZT0 to ZT20, as determined via RT-qPCR in granulosa cells after exposure to continuous light (n = 4 for all groups and timepoints). The data are presented as the mean ± SEM. Statistical analysis was performed via one-way ANOVA with Tukey's multiple comparison post hoc test. *P < 0.05; **P < 0.01; ***P < 0.001; ****P < 0.0001.

Metabolic shifts are often accompanied by changes in gene expression, as these biological processes are intricately linked. We focused subsequent experiments on GCs and conducted transcriptome sequencing. The resulting volcano plot highlighted a substantial number of differentially expressed genes (DEGs) at both ZT0 and ZT12 across the NL, CL, and CN groups (Fig. 4a and Supplementary Fig. 6a). Further enrichment analysis of these genes revealed their significant roles in amino acid metabolism and autophagy pathways (Supplementary Fig. 6b-d).

Based on these findings, a Venn diagram overlap analysis revealed 1,655 genes that exhibited circadian rhythms under normal light conditions, thereby suggesting their disruption under continuous light and subsequent recovery (Fig. 4b). KEGG pathway annotation and enrichment analysis revealed the predominant involvement of these genes in glutathione metabolism, aminoacyl-tRNA biosynthesis, corticosterone synthesis and secretion, endocytosis, the cell cycle, and apoptosis (Fig. 4c).

Moreover, the differential gene enrichment pathways observed at ZT0 in the NL and CL groups were quite diverse, including pathways related to amino acid biosynthesis (such as arginine metabolism, leucine degradation, isoleucine degradation, valine degradation, and glutamine metabolism), as well as circadian rhythm regulation, phagosome function, Fc-γR-mediated phagocytosis, glycolysis, and gluconeogenesis (Fig. 4d). Additionally, the differential changes among the CL and CN groups were similar to the abovementioned changes (Supplementary Fig. 6e).

We utilized qPCR to assess the rhythmic expression of genes related to leucine transport (Scl3a2), degradation (Bcat1 and Dld), and signal transduction (Sestrin2 and Sestrin3), as well as core clock-related genes in GCs, including Bmal1, Rev-erbα, Rev-erbβ, Per1, Per2, Per3, Cry2, Npas2, and Dbp. Further evidence supporting the disruption of circadian rhythms was observed in our study. The serum corticosterone levels in the NL group typically peaked at ZT12, and food consumption exhibited a diurnal pattern, with lower intake being observed during the day and higher intake being observed at night. However, these rhythmic patterns were disrupted under continuous light exposure (Supplementary Fig. 6f). Our findings indicated that these genes exhibited rhythmic expression patterns in the NL group; however, their rhythms and expression levels were significantly altered in response to continuous light exposure (Fig. 4e and f).

### Circadian expression of genes involved in the leucine-mTOR-autophagy and apoptosis pathways in GCs is disrupted by continuous light

**Figure 5:**
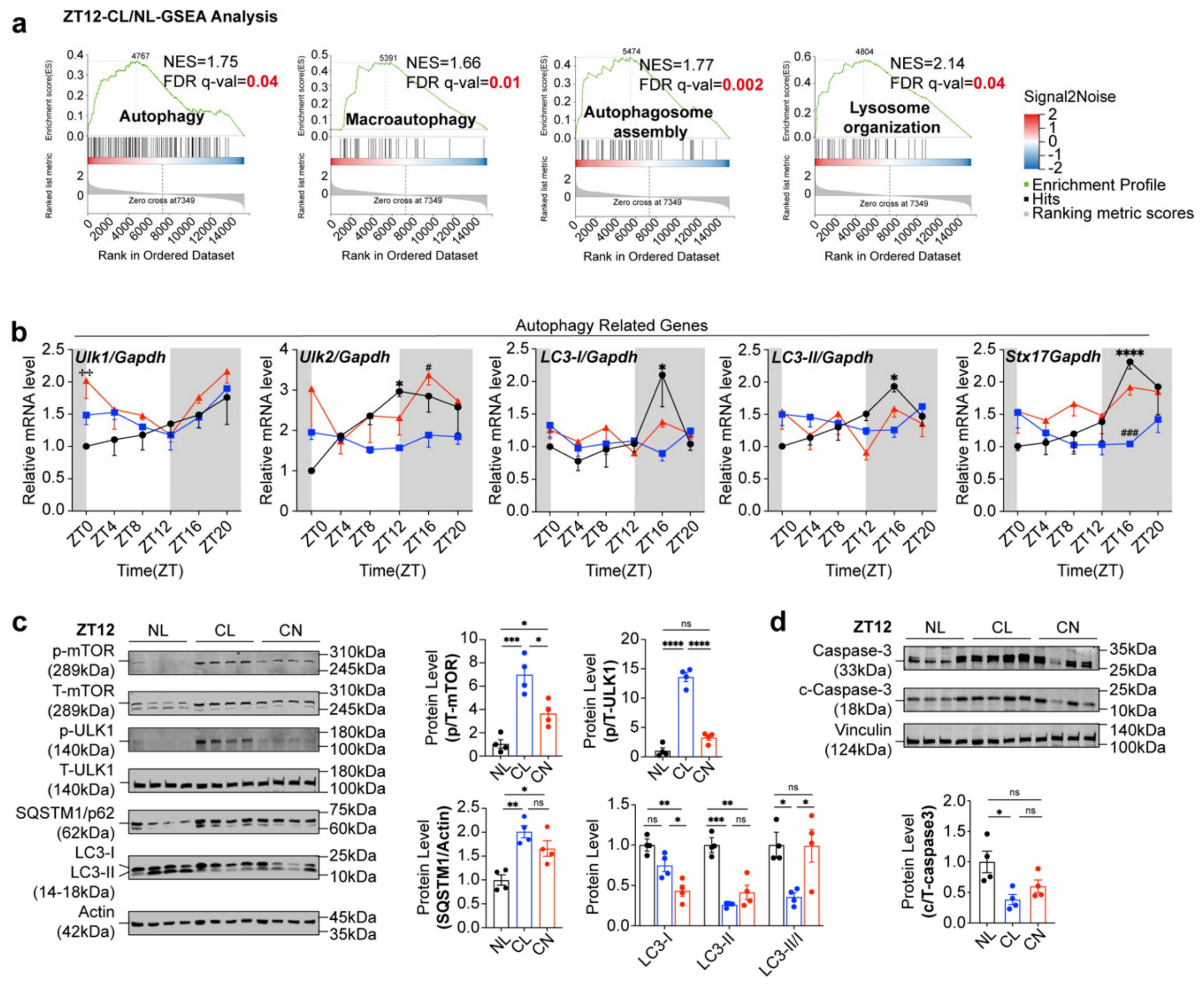
** Circadian expression of the leucine-mTOR-autophagy and apoptosis pathways in GCs was disrupted by continuous light exposure. (a)** The autophagy pathway was identified via GSEA by comparing the CL and NL groups at ZT12. **(b)** Circadian expression of autophagy pathway-related genes was disrupted via continuous light exposure (n = 4 for all groups). **(c)** The expression levels of leucine-mTOR-autophagy pathway-related proteins in the three groups at ZT12 were analyzed and are displayed as a bar chart (n = 4 for all groups). **(d)** The expression levels of apoptosis-related proteins in the three groups at ZT12 were analyzed and are displayed as a bar chart (n = 4 for all groups). The data are presented as the mean ± SEM. Statistical analysis was performed via one-way ANOVA with Tukey's multiple comparison post hoc test. *P < 0.05; **P < 0.01; ***P < 0.001; ****P < 0.0001.

At ZT12, GSEA revealed that the DEGs in the NL and CL groups were significantly enriched in pathways such as autophagy, lysosome organization, autophagosome assembly, and macroautophagy (Fig. 5a). Conversely, the DEGs in the CL and CN groups were predominantly enriched in pathways related to amino acid biosynthesis, arginine and proline metabolism, glutathione metabolism, aminoacyl-tRNA biosynthesis, circadian rhythm entrainment, and phagosome pathways (Supplementary Fig. 6g). Additionally, the expression of key autophagy-related genes (including Ulk1, Ulk2, LC3-I/II, and Stx17) was significantly disrupted (Fig. 5b).

When considering that alterations in autophagy-related genes predominantly occur nocturnally, we focused our analysis on the time interval ranging from ZT12 to ZT24. We initially assessed the diurnal rhythms of the ratio of phosphorylated mTOR to total mTOR (p/T-mTOR) and LC3 expression in both the NL and CL groups. The results revealed robust rhythmic fluctuations in the expression levels of p/T-mTOR and LC3 in the NL group. Specifically, mTOR activity gradually increased from ZT12 and peaked at ZT16; moreover, it was accompanied by a concurrent suppression of the autophagic wave. In contrast, these rhythmic patterns were abolished in the CL group (Supplementary Fig. 7a and b). We subsequently further examined the expression of autophagy-related proteins at different timepoints at night in the NL, CL, and CN groups. Our findings revealed that, compared with that in the NL group, the phosphorylation of mTOR and ULK1 at ZT12 in the CL group increased. The significant increase in p62 expression at ZT12 indicated a decrease in autophagic activity and the LC3-II/I ratio (Fig. 5c). In the NL group, mTOR phosphorylation gradually increased and peaked at ZT16, after which point the differences between the groups became less pronounced. After ZT16, mTOR phosphorylation began to decrease in the NL group (particularly at ZT20). However, compared with the NL group, the CL group maintained higher mTOR phosphorylation levels, which was accompanied by reduced autophagic activity. At ZT24, both the mTOR and ULK1 phosphorylation levels remained elevated in the CL group compared with those in the NL group, although no significant differences were noted in the expression of the autophagy-related proteins p62 and LC3-II/I (Supplementary Fig. 7c-e). Notably, in the CN group, both mTOR phosphorylation levels and the expression of autophagy-related proteins were significantly restored. Consistent with the close relationship between autophagy, apoptosis, and cell survival, western blot analysis revealed a reduction in cleaved caspase-3 levels (Fig. 5d).

### Leucine can inhibit autophagy and apoptosis in GCs at high concentrations

**Figure 6:**
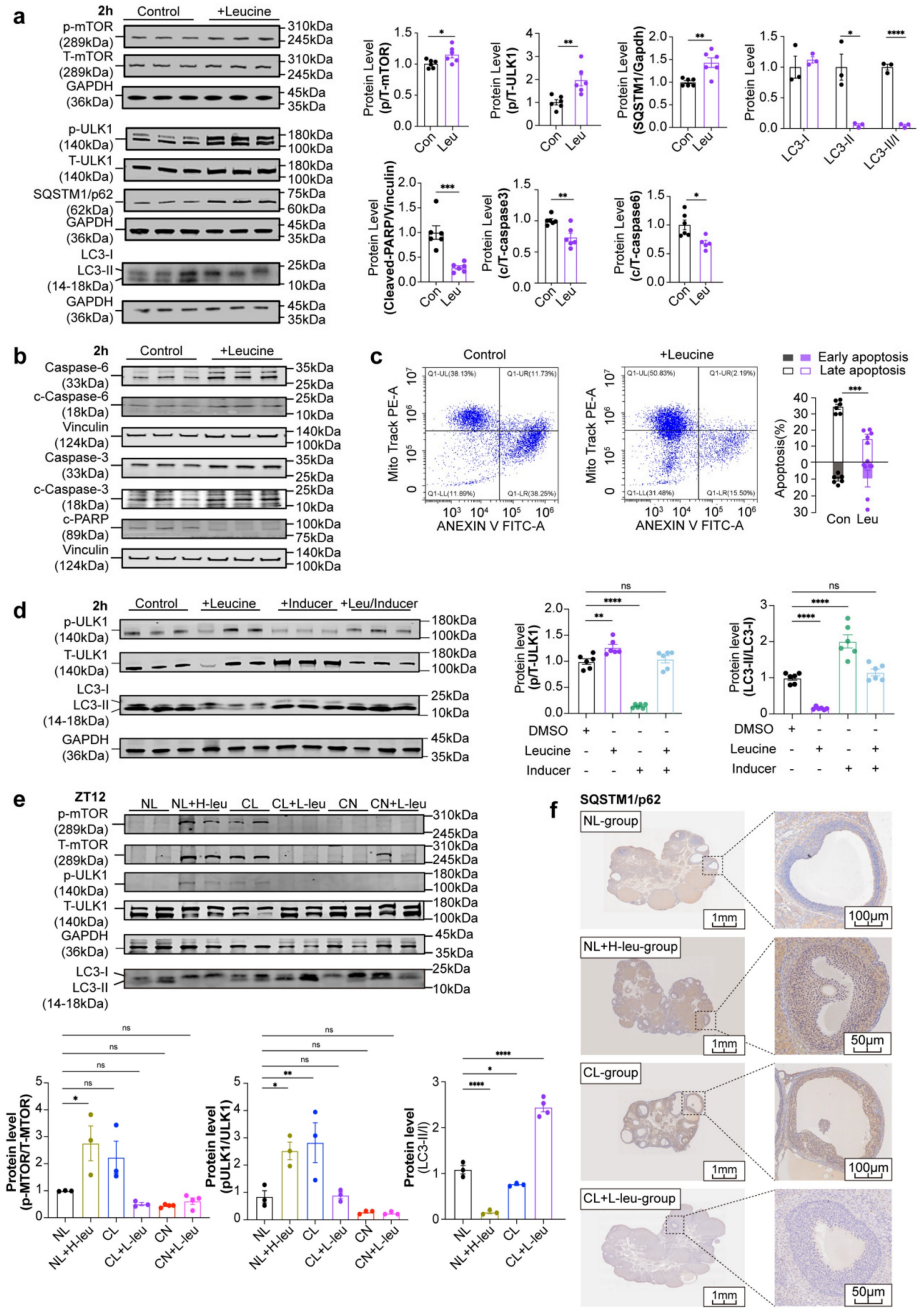
** High concentrations of leucine can inhibit autophagy and apoptosis in GCs. (a)** The expression levels of leucine-mTOR-autophagy pathway-related proteins were stimulated by a 3-fold increase in leucine for 2 h (n = 6 for all groups). **(b)** The expression levels of apoptosis-related proteins were also stimulated by a 3-fold increase in leucine for 2 h. The results were analyzed and are displayed as a bar chart (n = 6 for all groups). **(c)** Flow cytometry was used to detect changes in the level of apoptosis stimulated by high concentrations of leucine in GCs (n = 6 for all groups). The early phase of apoptosis was defined as annexin V+/Mito+ (solid square), whereas the late phase of apoptosis was defined as annexin V+/Mito- (hollow square). (d) Effects of leucine and autophagy inducers on GCs (n = 6 for all groups). (e) The expression levels of leucine-mTOR-autophagy pathway-related proteins in the six groups at ZT12 were analyzed and are displayed as a bar chart (n = 4 for all groups). (f) Representative images of immunohistochemical results of SQSTM1/p62 protein in the ovaries of NL, NL+H-leu, CL and CL+L-leu group. The data are presented as the mean ± SEM. Statistical analysis was performed via an unpaired two-sided Student's t test and one-way ANOVA with Tukey's multiple comparison post hoc test. *P < 0.05; **P < 0.01; ***P < 0.001; ****P < 0.0001.

To directly assess the influence of leucine on GCs, we extracted primary GCs from rats and exposed them to leucine in a time-dependent and dose-dependent manner. The most effective concentration and exposure duration were identified as a 3-fold increase in leucine for a period of 2 hours. This treatment led to significant increases in p-mTOR and T-mTOR levels, as well as the p/T-mTOR ratio, which correspondingly activated downstream total ULK1 (T-ULK1) and increased its phosphorylation. As a result, autophagic activity was suppressed, as indicated by a marked increase in p62 levels and a decrease in the LC3-II/I ratio (Fig. 6a). No significant changes were detected when the cells were treated for either 1 hour or 4 hours (Supplementary Fig. 8a-d). Furthermore, after leucine stimulation, the levels of apoptosis-related proteins, including cleaved caspase-3/6 and the downstream substrate cleaved-PARP, were noticeably decreased (Fig. 6b). These results were corroborated by the flow cytometry analysis, which revealed a significant decrease in the level of apoptosis in GCs under leucine stimulation (Fig. 6c).

To further demonstrate the ability of leucine to inhibit mTOR-ULK1-mediated autophagy in GCs, after stimulating GCs under time and concentration gradients, we treated GCs with 10 μM autophagy inducers for 2 hours as the optimal conditions (Supplementary Fig. 8e-f). Leucine upregulated p/T-ULK1 and inhibited LC3-II/I, whereas autophagy inducers downregulated p/T-ULK1 and activated LC3-II/I. Cotreatment neutralized both effects (Fig. 6d). To determine whether the phenotypes were associated with leucine-induced alterations in GC autophagy, we examined autophagic activity across these groups. Compared with that in the NL group, the mTOR-ULK1 axis was activated in both the NL+H-leu group and the CL group, thus leading to the inhibition of autophagy. In contrast, autophagy was significantly enhanced in the CL+L-leu group. No statistically significant differences were observed between the CN and CN+L-leu groups relative to the NL group (Fig. 6e and f).

### Positive correlation and causal relationship between leucine and PCOS according to evaluations of clinical serum samples and MR analysis

Our findings were demonstrated in both animal models and cell stimulation experiments, thus prompting us to extend our study to human patients to determine whether similar conclusions could be obtained. We measured the serum levels of BCAAs in clinical patients and assessed the relationships between BCAA levels and clinical characteristics. We subsequently conducted MR analysis with published GWAS data to evaluate causality. As of December 2020, 16 healthy volunteers in the control group and 29 patients in the PCOS group (including 12 patients with HA and 17 patients without HA) were recruited. Analysis of the baseline characteristics revealed a significantly prolonged menstrual cycle in the PCOS group, along with increased serum AMH and LH levels and LH/FSH ratios. Notably, the antral follicle count (AFC) was significantly greater only in the HA-PCOS subgroup (Supplementary Table 1).

**Figure 7:**
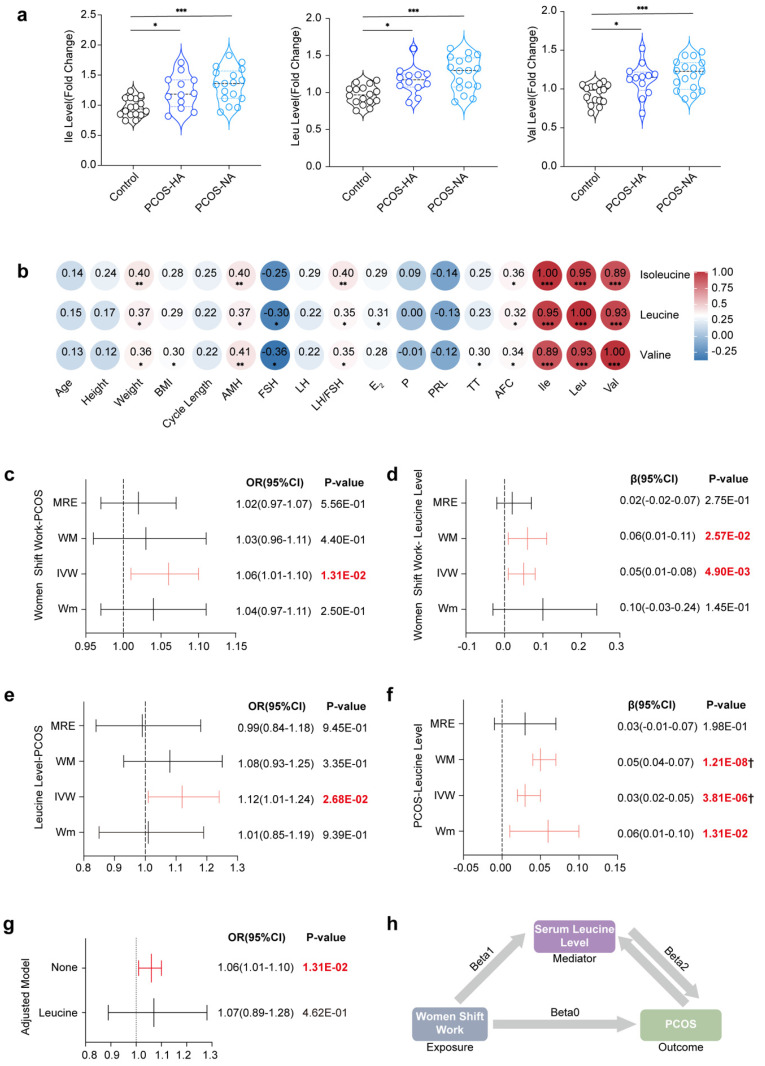
** Positive correlation and causal relationship between leucine and PCOS in clinical serum samples and MR analysis. (a)** Serum levels of leucine, isoleucine, and valine in women in the control and PCOS groups are indicated in black (control, n = 16), blue (PCOS-HA, n = 12), and light blue (PCOS-NA, n = 17), respectively. **(b)** Correlations between leucine, isoleucine, or valine and clinical reproductive markers. **(c)** Causality results of MR between the night shift work of women and PCOS. **(d)** Causality results of MR between the night shift work of women and serum leucine levels. **(e)** Causality results of MR between serum leucine levels and PCOS. **(f)** Reverse causality results of MR between PCOS and serum leucine levels. **(g)** Causality results of multivariable MR between the night shift work of women, serum leucine levels, and PCOS. **(h)** Summary diagram illustrating the causal relationships among the night shift work of women, serum leucine levels, and PCOS. The data are presented as the mean ± SEM. Statistical analysis was performed via one-way ANOVA with Tukey's multiple comparison post hoc test. *P < 0.05; **P < 0.01; ***P < 0.001; ****P < 0.0001; Bonferroni correction †*P* <0.05/42.

The results indicated that serum isoleucine, leucine, and valine levels were significantly higher in the PCOS group than in the control group, regardless of HA status (Fig. 7a). There were no significant differences observed in age or BMI among the three groups, thus eliminating the need for adjustments for confounding factors. Furthermore, the levels of these three serum BCAAs (including AMH, LH/FSH, and the AFC) were closely correlated with the endocrine and metabolic indices of patients with PCOS. Correlation analysis revealed that only valine was positively correlated with BMI and TT, whereas only leucine was positively correlated with E_2_ (Fig. 7b).

In the Mendelian randomization analysis, the initial results indicated a significant association between female night shift work and the occurrence of PCOS (OR_IVW_=1.06, 95% CI [1.01-1.10], *P=1.31E-02*) (Fig. 7c). However, no significant effects on the levels of hormones such as TT (OR_IVW_=0.00, 95% CI [-0.02-0.01], *P=7.07E-01*), Bio-T (OR_IVW_=-0.02, 95% CI [-0.04-0.00], *P=7.22E-02*), or SHBG (OR_IVW_=0.003, 95% CI [-0.006-0.013], *P=5.11E-01*) were detected (Supplementary Fig. 9 and Supplementary Table 9). The heterogeneity and pleiotropy results are shown in Supplementary Figs. 10-13 and Supplementary Tables 10-11, with further analysis being conducted via the MRPRESO package to identify sources of heterogeneity. After excluding outliers, the causal correlation between female night shift work and Bio-T was adjusted (β_MRPRESSO_ = -0.02, 95% CI [-0.04- -0.00], *P=5.36E-02*).

Additionally, a positive correlation was observed between female night shift work and serum leucine levels (β_IVW_=0.05, 95% CI [0.01-0.08], *P=4.93E‒03*) (Fig. 7d), with corresponding forest plots, funnel plots, leave-one-out plots, and scatter plots provided in Supplementary Figs. 14-18. We subsequently demonstrated that serum leucine may contribute to the development of PCOS (OR_IVW_=1.12, 95% CI [1.01-1.24], *P=2.68E-02*) (Fig. 7e). Although it had no significant effect on TT (β_IVW_=-0.07, 95% CI [0.16-0.02], *P=1.19E-01*), it had a positive influence on bioavailable testosterone (Bio-T) (β_IVW_=0.12, 95% CI [0.04-0.20], *P=5.25E-03*) and a reduction in SHBG levels (β_IVW_=-0.16, 95% CI [-0.26- -0.06], *P=1.96E-03*) (Supplementary Fig. 19). Conversely, our results indicated that PCOS could also increase serum leucine levels (β_IVW_=0.03, 95% CI [0.02-0.05], *P=3.81E-06*) (Fig. 7f).

Furthermore, Bio-T promoted an increase in leucine levels (β_IVW_=0.06, 95% CI [0.02-0.10], *P=6.71E-03*), whereas SHBG inhibited leucine levels (β_IVW_=-0.20, 95% CI [0.25-0.14], *P=4.52E-13*). Given the presence of heterogeneity and pleiotropy, the following corrections for the results were made: leucine-Bio-T (β_MRPRESSO_=0.09, 95% CI [0.04-0.13], *P=4.78E-04*), Bio-T-leucine (β_MRPRESSO_=0.05, 95% CI [0.01-0.09], *P=1.07E-02*), leucine-SHBG (β_MRPRESSO_=-0.10, 95% CI [0.14-0.06], *P=2.20E-05*), and SHBG-leucine (β_MRPRESSO_=-0.17, 95% CI [-0.22- -0.13], *P=6.61E-13*) (Supplementary Fig. 19-23). Overall, we propose that the elevated risk of PCOS associated with shift work may be mediated by increased serum leucine levels (OR_IVW_=1.06, 95% CI [1.01-1.10], *P=1.31E-02*) (Fig. 7g and h).

## Discussion

Our study provides compelling evidence that continuous light exposure significantly impairs ovulatory function in female rats, as evidenced by the disruption of reproductive hormone levels and the development of polycystic ovarian morphology. A critical finding of this study involved the role of leucine, for which an abnormal rhythm and elevated levels were observed to be linked to the disruption of autophagy patterns in GCs, thus leading to the onset of disease. These findings are further supported by the observation that the levels of BCAAs (particularly leucine) were markedly elevated in the PCOS group (compared with those in the control group) and positively correlated with reproductive markers such as AMH, FSH, and the AFC. Our Mendelian randomization analysis suggests that shift work may promote the development of PCOS in women via elevated circulating leucine levels, thereby resulting in the first integration of animal experiments with clinical and genetic information systems to elucidate potential mechanisms and causal relationships between rhythm disorders, serum leucine levels, and ovulatory dysfunction.

A comprehensive observation of phenotypes revealed that AMH (which is predominantly secreted by small antral follicles) corresponds to an increased number of preantral follicles, which aligns with the findings of previous studies 33, 34. Compared with that in the NL group, the number of retrieved oocytes in the CL group was significantly lower, thus indicating poor ovarian response (POR) due to continuous light exposure. High circulating AMH levels were observed to inhibit primordial follicle initiation and follicle sensitivity to FSH, thereby leading to resistance to hMG ovulation induction and necessitating a higher starting dose 35. This study revealed that the CL group required higher doses and longer intervals during the ovulation induction cycle, thus underscoring the detrimental effects of continuous light exposure on ovulatory function and emphasizing the need for further research into potential interventions.

The circadian rhythm system is extensively distributed across various organs of the reproductive system, including the hypothalamus, pituitary gland, and gonads/adrenal glands 28, 36. Prior research has consistently demonstrated that such exposure suppresses LH peaks without altering FSH levels, with this finding being corroborated by our serum data. In contrast to the central circadian regulation of the HPO axis (which exhibits minimal intrinsic rhythmicity in hypothalamic-pituitary tissues), we identified a leucine-mediated mTORC1 pathway in ovarian GCs that disrupts autophagy and represents a novel metabolic rhythm mechanism contributing to ovulation disorders 37. Moreover, qPCR analyses revealed that continuous light exposure disrupts the circadian rhythm of clock genes in GCs, which is a phenomenon that has also been observed in liver and adipose tissues 38, 39. Disruptions to the circadian system, such as artificial night light exposure, irregular eating patterns, and shift work, can lead to disturbances in hormonal rhythms and metabolic processes 40. The endogenous clock significantly influences human metabolic pathways independent of sleep or feeding 41, 42, and significant metabolic changes have been observed after the knockout of certain core clock genes 43-45. The 600-lux intensity was selected for continuous light exposure due to its established role in modeling circadian disruption in rodents, as well as its translational relevance to human shift-work environments. This intensity aligns with our standardized protocols for inducing metabolic and endocrine disturbances 18 and is consistent with prior studies that have reproduced PCOS-like features (such as ovarian morphological changes and hyperandrogenism) after prolonged exposure in rodents 46. Furthermore, a light intensity of 600 lux corresponds to illumination levels that are commonly reported in industrial and healthcare settings (300-500 lux) and has been extensively used in chronobiology research 47, 48, thus underscoring its physiological and clinical relevance for the investigation of long-term effects of light exposure at night. Our study revealed that continuous light exposure not only affected the expression of clock genes but also disrupted metabolism-related genes and metabolite rhythms, thereby enriching pathways that are primarily related to amino acid and lipid metabolism.

Transcriptome analysis revealed evident changes in both metabolism and the autophagy pathway. Differences in gene enrichment between the PCOS and control groups were noted in terms of steroid biosynthesis and metabolism, amino acid biosynthesis, and the HIF-1 pathway 49. The rhythmic expression pattern of autophagy-related genes in the NL group was disrupted in the CL group, thus suggesting that disturbances in circadian rhythm, metabolic disorders, and autophagy contribute to PCOS pathogenesis 50. Notably, extensive research has consistently identified leucine as the metabolic marker that is most strongly associated with PCOS risk 51, 52. Subsequent investigations further identified leucine (rather than other BCAAs) as the primary focus of this study due to its more pronounced and consistent dysregulation under continuous light exposure in both the systemic circulation and GCs. Moreover, leucine is well known as the most potent natural activator of the mTOR signaling pathway, which is a central regulator of cell metabolism, proliferation, and autophagy (all of which are critically involved in GC function and folliculogenesis). Although isoleucine also participates in cellular metabolic regulation, its effect on mTOR is relatively weak 21, 53, 54. Our findings suggest that leucine-induced changes in both the serum and GCs in conjunction with disordered circadian rhythms may contribute to the development of PCOS, which is similar to observations in mice. However, although leucine restriction improved certain phenotypic features, its therapeutic effect appeared to be relatively modest. This limited efficacy may be attributed to the strength of light exposure, which likely attenuated the benefits of low-leucine intervention. Notably, the most significant phenotypic improvement was observed only when leucine restriction and circadian modulation were combined, thus suggesting a synergistic therapeutic approach.

Lifestyle modifications are the primary approach for treating PCOS 55, with dietary methods such as restricted diets, the Mediterranean diet, and low-protein diets representing mainstream options. BCAA levels are associated with insulin resistance in women with PCOS and conditions such as type 2 diabetes and obesity 56, although the causal relationship remains unclear. Increased BCAA intake has been observed to promote the development of IR and lipid accumulation 57, whereas a leucine-deficient diet results in reductions in fasting blood glucose levels, insulin content, and the insulin resistance index in women with PCOS 58. Our study revealed a positive correlation between serum leucine levels and AMH levels in women with PCOS, thus further supporting the involvement of BCAAs in PCOS pathogenesis.

Mendelian randomization (MR), which uses single nucleotide polymorphisms (SNPs) as instrumental variables, offers reduced bias and increased confidence in assessing the causal relationship between exposure and outcome. Although our MR analysis indicated a potential causal influence of leucine on PCOS, the limitations inherent to MR (including possible horizontal pleiotropy, instrument strength, and the predominant reliance on European genetic datasets) constrain the generalizability and robustness of our findings. Consequently, these results should be interpreted as preliminary evidence supporting a role for leucine in PCOS pathogenesis. Further validation in clinically and ethnically diverse prospective cohorts is essential. We recommend integrating longitudinal measures of serum leucine within large-scale biobank resources (such as the China Kadoorie Biobank or UK Biobank), wherein well-phenotyped PCOS subcohorts could be established to track dynamic leucine changes and establish correlations between baseline levels and the incidence of PCOS. Additionally, future efforts should incorporate expanded multiancestry GWAS meta-analyses and multivariable MR frameworks to adjust for concomitant metabolic traits, thereby refining the causal estimate and enhancing translational relevance to clinical populations.

## Conclusion

In conclusion, continuous light exposure disrupts ovarian function by altering the leucine-mTOR-autophagy axis in granulosa cells, thus leading to polycystic ovary-like phenotypes and elevated AMH levels. Dietary leucine modulation replicates or mitigates these effects, thereby highlighting its pivotal role in ovulation disorders. Clinical data further support the association between elevated leucine levels, hormonal imbalances, and PCOS pathogenesis, whereas Mendelian randomization suggests that night shift work may increase the risk of PCOS via leucine accumulation. These findings reveal a mechanistic link between environmental light disruption, metabolic dysregulation, and reproductive dysfunction, thus offering potential dietary or therapeutic strategies for the management of PCOS.

## Supplementary Material

Supplementary figures and tables.
