# Supplementary material for: The Leucine-mTOR-Autophagy Axis in Granulosa Cells Mediates Circadian Disruption-Induced Anovulation
Source: Int J Biol Sci. 2026 Jan 1;22(1):201–19. doi: 10.7150/ijbs.116803 (PMC12681872; doi:10.7150/ijbs.116803)
Supplement: Supplementary file 1 — Supplementary figures and tables. [file ijbsv22p0201s1.pdf]

Supplementary Figures:

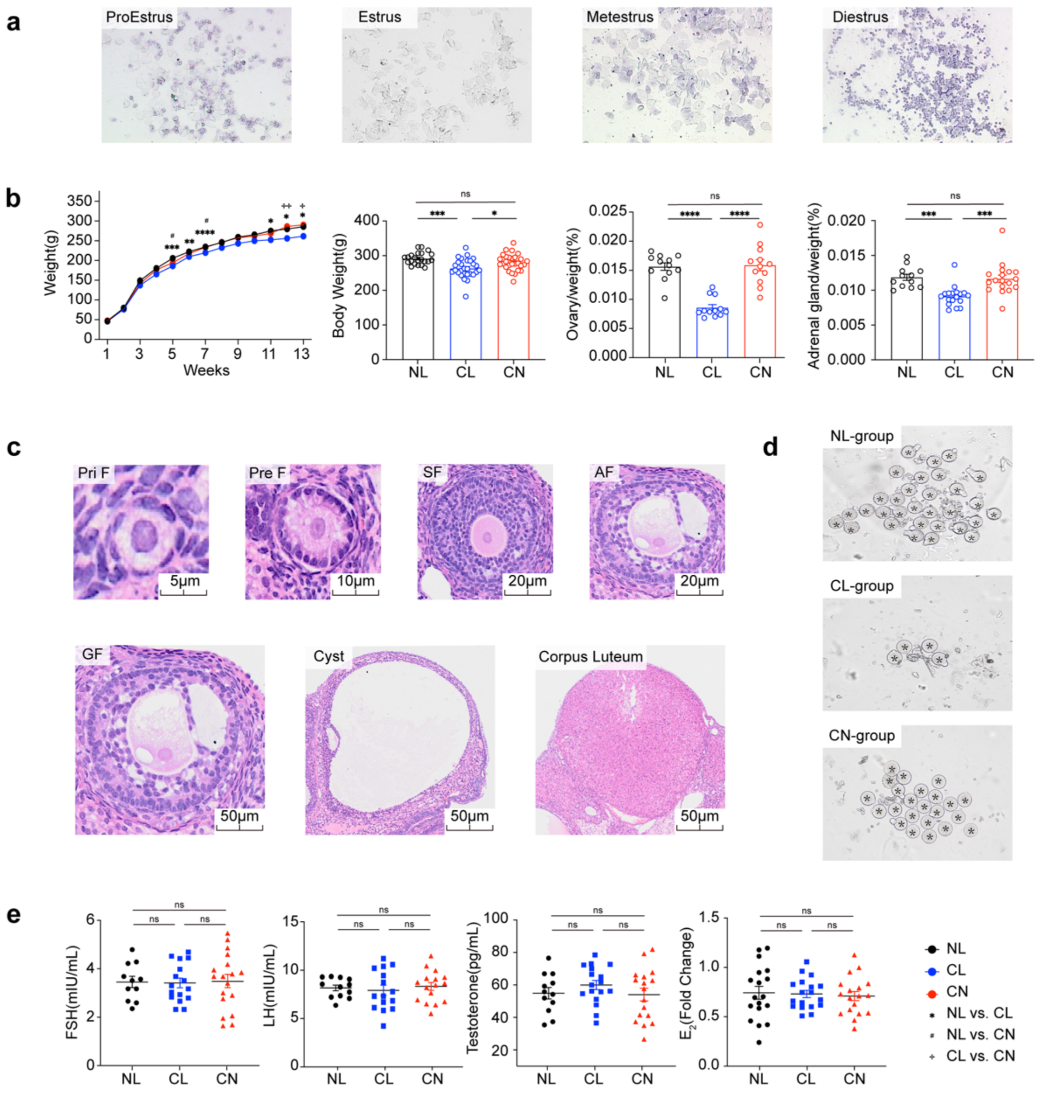

Supplementary Fig. 1: The reproductive phenotypes of rats exposed to continuous light. (a) Vaginal smear results represent four periods of the estrous cycle (P, proestrus; E, estrus; M, metestrus; DI, diestrus). **(b)** Growth and development of rats including body weight (n = 22,28,28 for NL, CL and CN group respectively), ovary (n = 12 for all groups) and adrenal gland index (n = 12,18,18 for NL, CL and CN group respectively). Ovarian Index (%) = Ovarian Weight (mg)/Body Weight (g). **(c)** Follicles in different follicular development stage. **(d)** Microscopic observation of recruited oocytes number after PMSG-hCG ovulation induction. **(e)** Serum levels of FSH, LH, TT (n = 12,16,18 for NL, CL and CN group respectively) were tested by ELISA and E_2 level were collected from untargeted metabolites results. Data are presented as mean ± SEM. Statistical analysis was performed with one-way ANOVA with Tukey's multiple comparison post-hoc test. **P* < 0.05; ***P* < 0.01; ****P* < 0.001; *****P* < 0.0001.

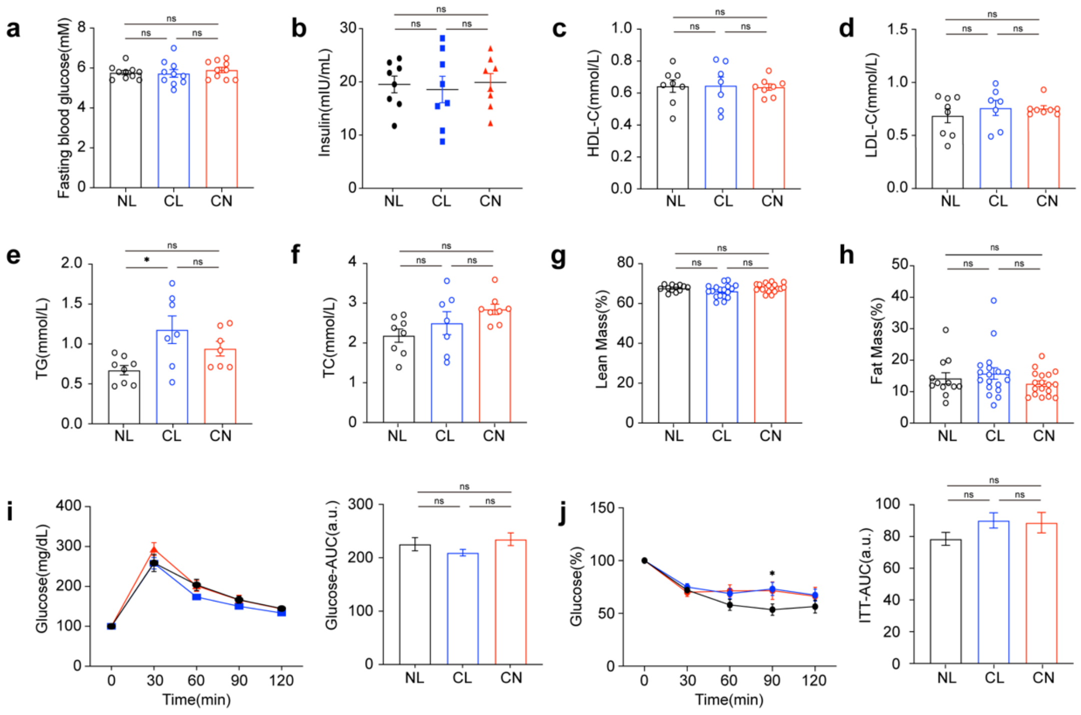

Supplementary Fig. 2: Detection of glucose and lipid metabolism indexes. (a) The level of fasting blood glucose (n =10 for all groups). **(b)** The level of serum insulin (n = 8 for all groups). **(c-f)** The level of serum HDL-C, LDL-C, TG and TC (n = 8, 7, 8 for NL, CL and CN group respectively). **(g-h)** The percentage of lean mass and fat mass (n = 12, 18, 18 for NL, CL and CN group respectively). **(i-j)** The results of glucose tolerance test (GTT) and insulin tolerance test (ITT) (n = 10, 10, 9 for NL, CL and CN group respectively). Data are presented as mean ± SEM. Statistical analysis was performed with one-way ANOVA with Tukey's multiple comparison post-hoc test. *$P < 0.05$; **$P < 0.01$; ***$P < 0.001$; ****$P < 0.0001$.

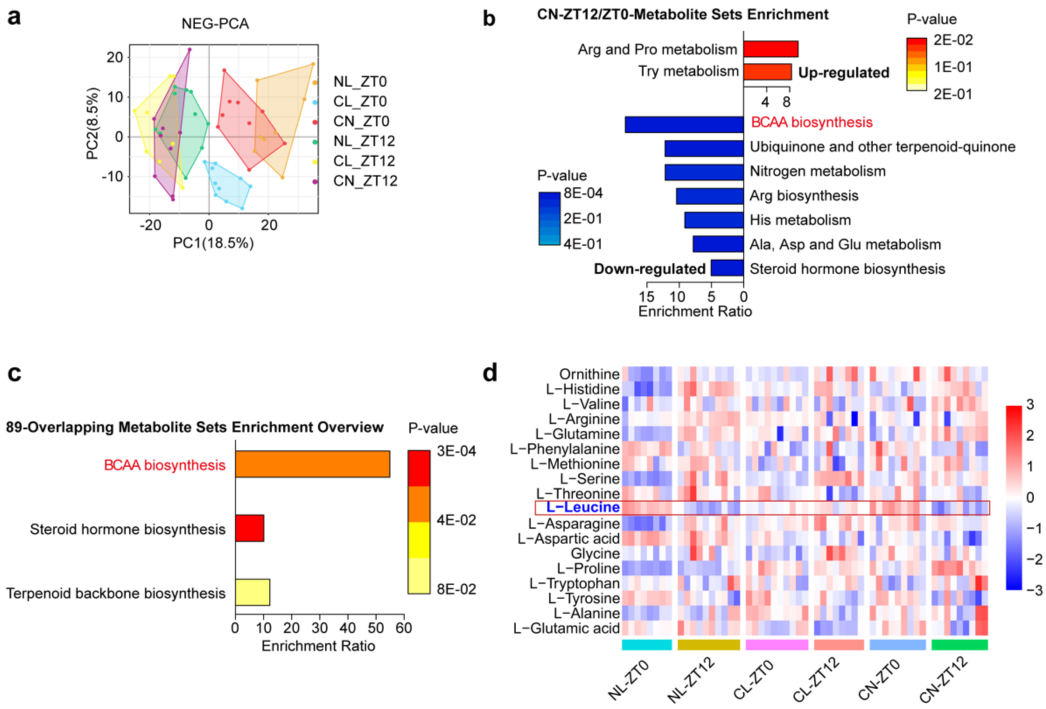

Supplementary Figure. 3: Continuous light result in leucine level changed both in serum and GCs. (a) PCA score plots derived from LC-MS data of serum samples with different time points at ZT0 and ZT12 in three groups of rats (n = 8,10,9 for NL, CL and CN group at ZT0; n = 10,8,9 for NL, CL and CN group at ZT12). Panels show the PCA score in NEG mode. **(b)** Top KEGG enrichment pathways from differential metabolites of comparing ZT12/ZT0 in CN groups. **(c)** Top 20 KEGG enrichment pathways from 89 differential metabolites. **(d)** Serum amino acid levels presented in heatmap.

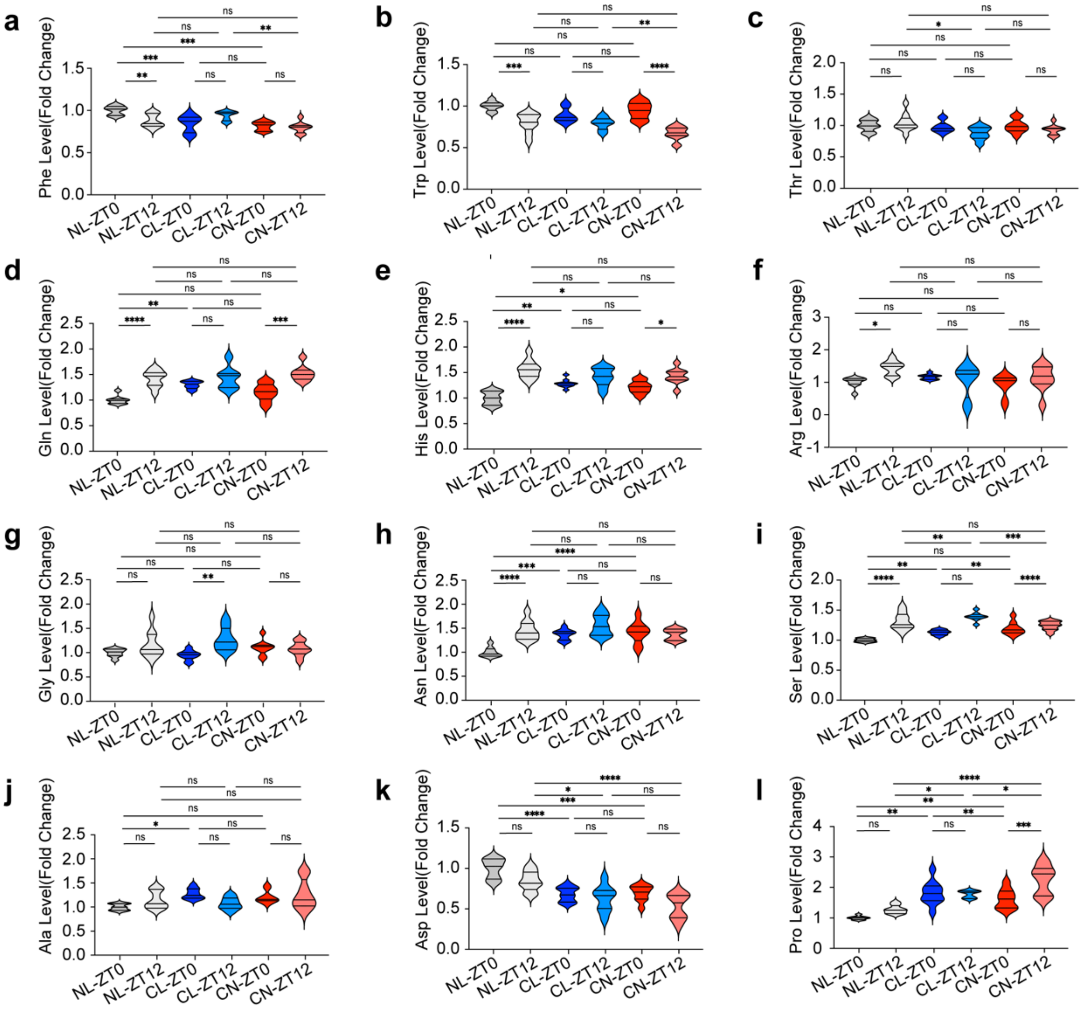

Supplementary Figure. 4: The significant circadian changes of amino acids in serum. (a-i) Serum level of Phe, Trp, Thr, Gln, His, Arg, Gly, Asn, Ser, Ala, Asp, Pro (n = 8,10,9 for NL, CL and CN group at ZT0; n = 10,8,9 for NL, CL and CN group at ZT12). Statistical analysis was performed by means of two-way ANOVA with Sidak's multiple comparisons. $*P < 0.05$; $**P < 0.01$; $***P < 0.001$; $****P < 0.0001$.

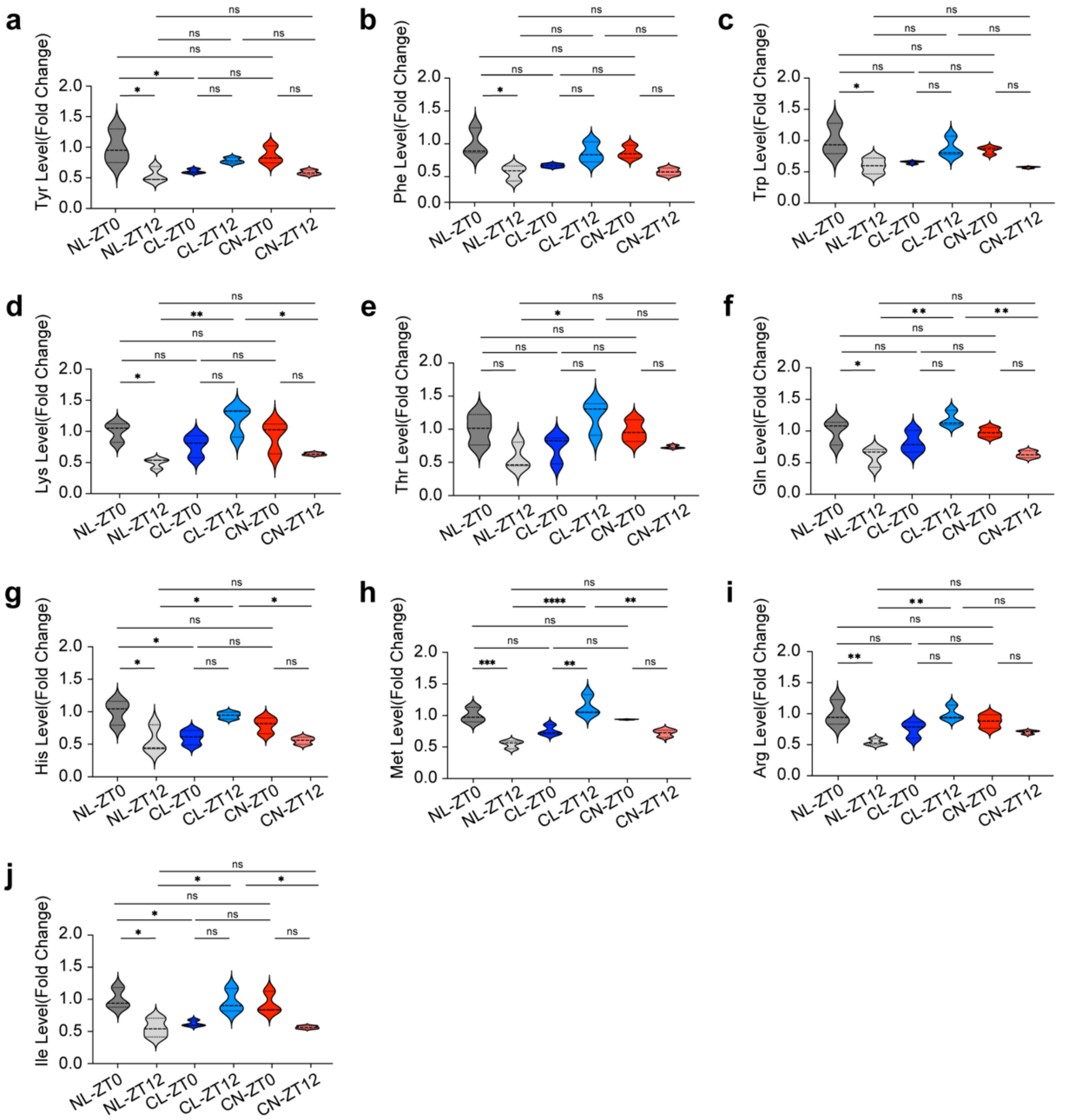

Supplementary Figure. 5: The significant circadian changes of amino acids in GCs. (a-j) Level of Tyr, Phe, Trp, Lys, Thr, Gln, His, Met, Arg, Ile in GCs (n = 3 for all groups). Statistical analysis was performed by means of two-way ANOVA with Sidak's multiple comparisons. **P* < 0.05; ***P* < 0.01; ****P* < 0.001; *****P* < 0.0001.

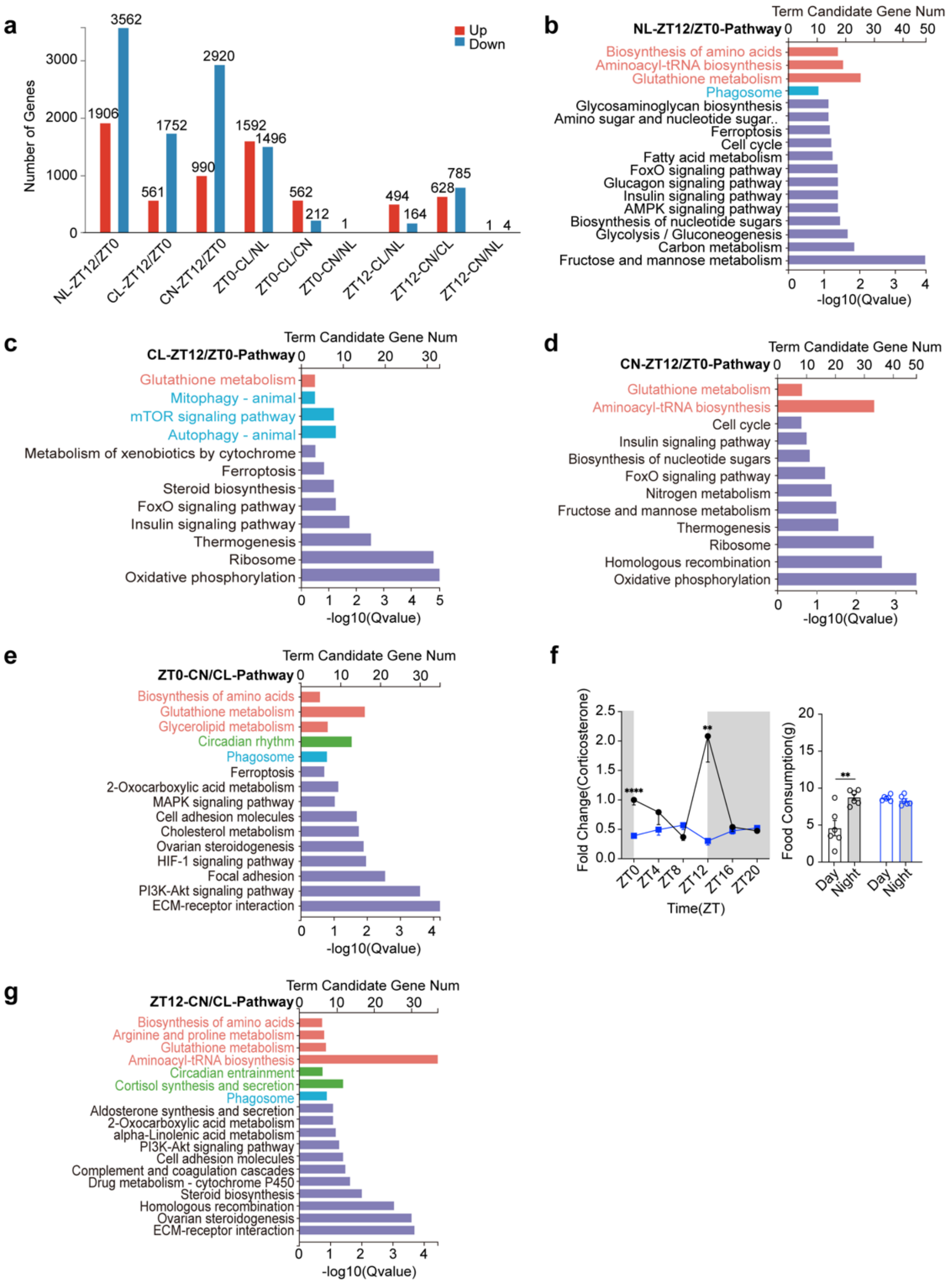

Supplementary Figure. 6: The profiles of the transcriptome in GCs reflect the interference of peripheral circadian rhythms by continuous light. (a) Bar plot shown differential genes in all comparison of groups. **(b-d)** Top 20 KEGG enrichment pathway from differential genes of comparing ZT12/ZT0 in NL, CL and CN groups. **(e)** Top 20 KEGG enrichment pathway from differential genes of comparing CL/CN at ZT0. **(f)** Serum circadian level of corticosterone (n = 5 for all groups at different timepoints). Circadian change of food consumption (n = 6 for all groups). **(g)** Top 20 KEGG enrichment pathway from differential genes of comparing CL/CN at ZT12. The data are presented as the mean ± SEM. Statistical analysis was performed by means of unpaired two-sided Student's t-test. *P < 0.05; **P < 0.01; ***P < 0.001; ****P < 0.0001.

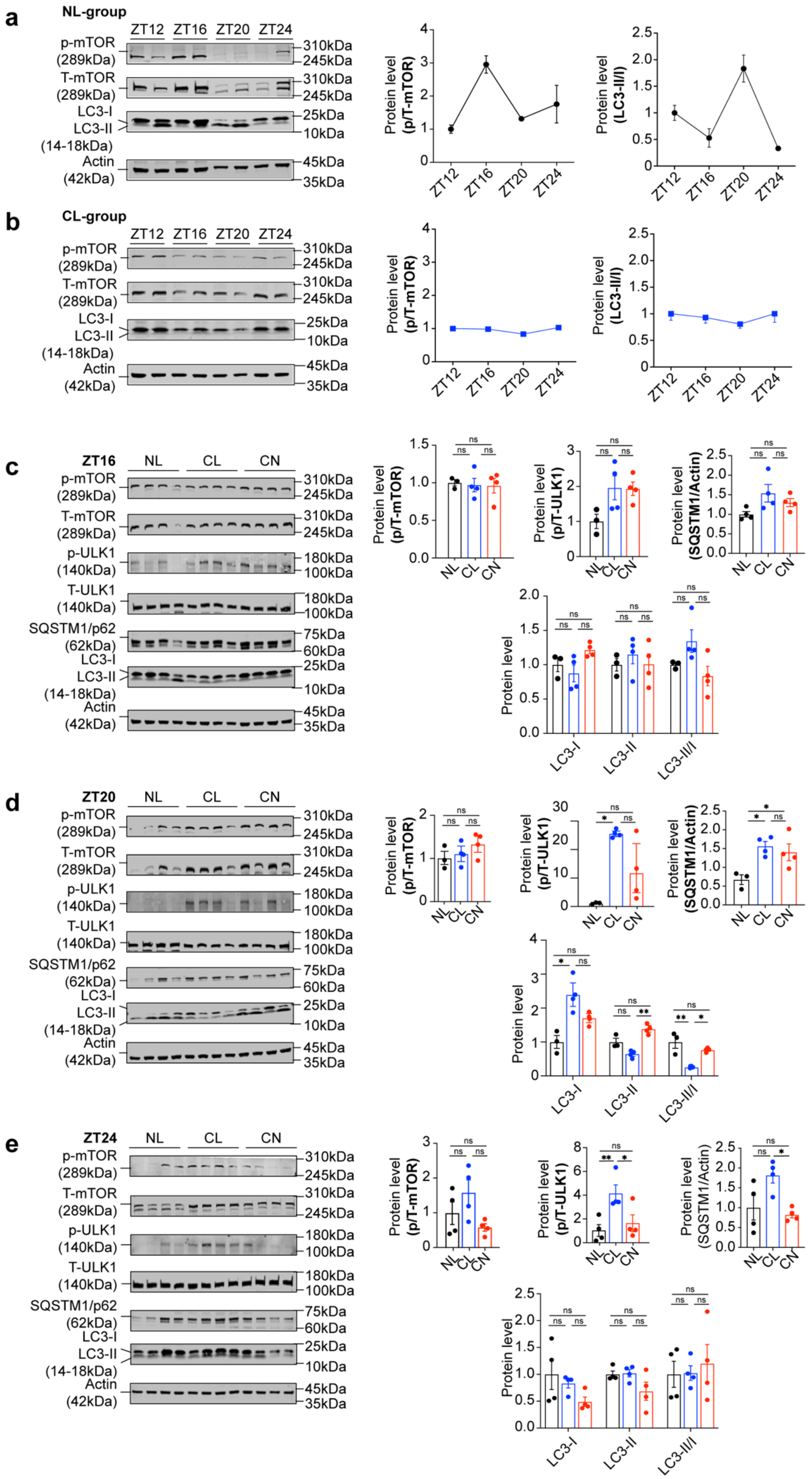

a
NL-group
ZT12 ZT16 ZT20 ZT24
p-mTOR (289kDa)
T-mTOR (289kDa)
LC3-I
LC3-II (14-18kDa)
Actin (42kDa)
310kDa
245kDa
25kDa
10kDa
45kDa
35kDa
Protein level (p/T-mTOR)
Protein level (LC3-II/I)
b
CL-group
c
ZT16
NL CL CN
p-ULK1 (140kDa)
T-ULK1 (140kDa)
SQSTM1/p62 (62kDa)
180kDa
100kDa
75kDa
60kDa
Protein level (p/T-ULK1)
Protein level (SQSTM1/Actin)
Protein level
LC3-I LC3-II LC3-II/I
ns
d
ZT20
e
ZT24

Supplementary Figure. 7: The circadian expression of the leucine-mTOR-autophagy and apoptosis pathway was disturbed by continuous light. (a-b) Expression level of leucine-mTOR-autophagy pathway-related proteins in NL and CL groups at ZT12, ZT16, ZT20 and ZT24 were analyzed and displayed as a line chart (n = 4 for all groups). **(c-e)** Expression level of leucine-mTOR-autophagy pathway-related proteins in NL, CL and CN groups at ZT16, ZT20 and ZT24 were analyzed and displayed as a bar chart (n = 3/4 for NL group and n = 4 for CL and CN groups). Data are presented as mean ± SEM. Statistical analysis was performed with one-way ANOVA with Tukey's multiple comparison post-hoc test. *$P < 0.05$; **$P < 0.01$; ***$P < 0.001$; ****$P < 0.0001$.

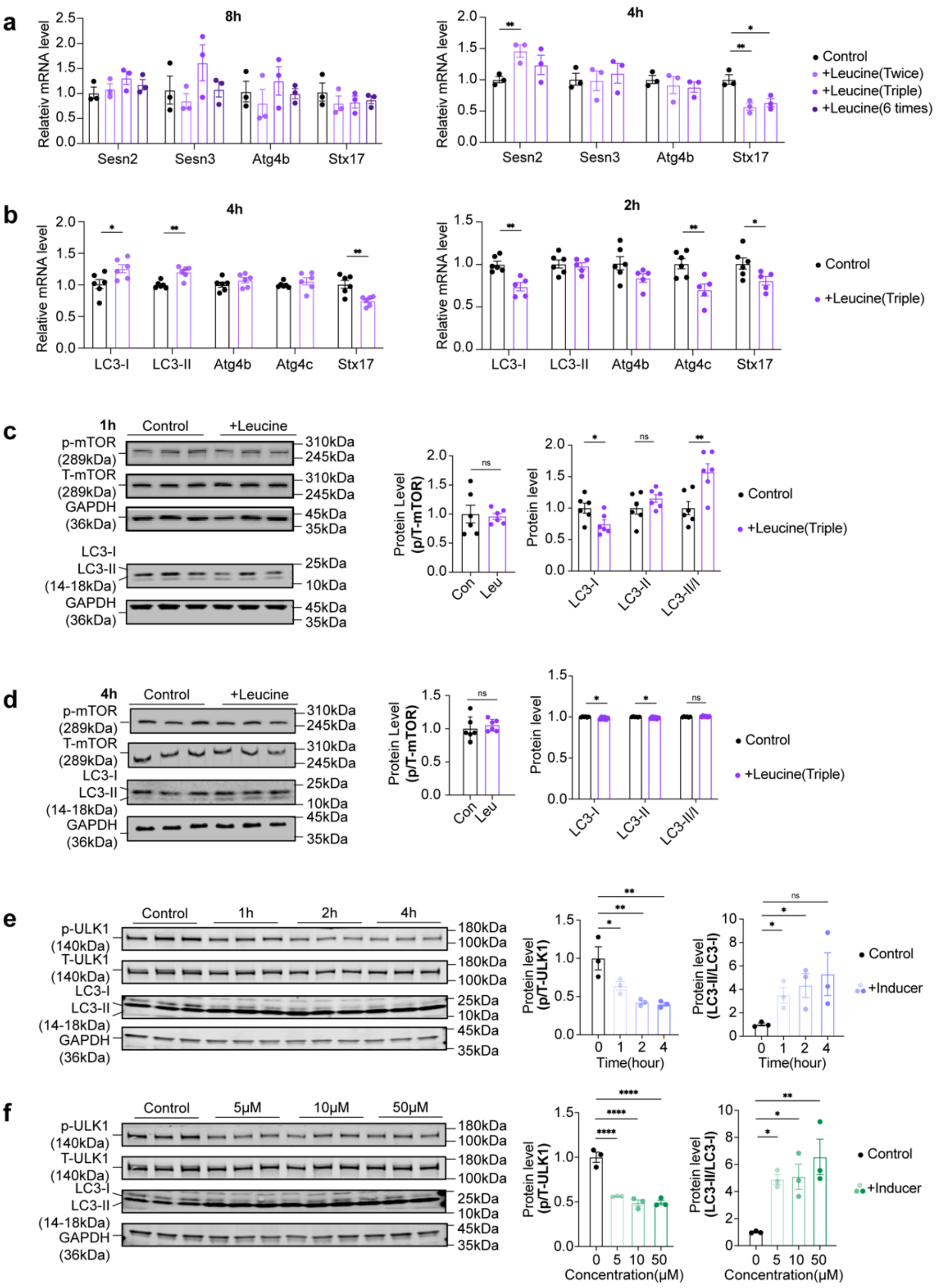

Supplementary Figure. 8: The level of mTOR-autophagy related proteins in GCs was stimulated by high-concentration leucine in vitro. (a) Expression level of mTOR-autophagy related mRNA which was stimulated by twice, triple, and 6-fold times leucine for 8h and 4h (n = 3 for all groups)**. (b)** Expression level of mTOR-autophagy related mRNA which was stimulated by triple leucine for 4h and 2h (n = 6 for control, n = 5 for leucine groups). **(c-d)** Expression level of mTOR-autophagy related proteins which was stimulated by 3-fold times leucine for 1h and 4h (n = 4 for all groups). **(e-f)** The time and dosage-dependent effect of autophagy inducer on GCs (n = 3 for all groups. Data are presented as mean ± SEM. Statistical analysis was performed with unpaired two-sided Student's t-test. **P* < 0.05; ***P* < 0.01; ****P* < 0.001; *****P* < 0.0001.

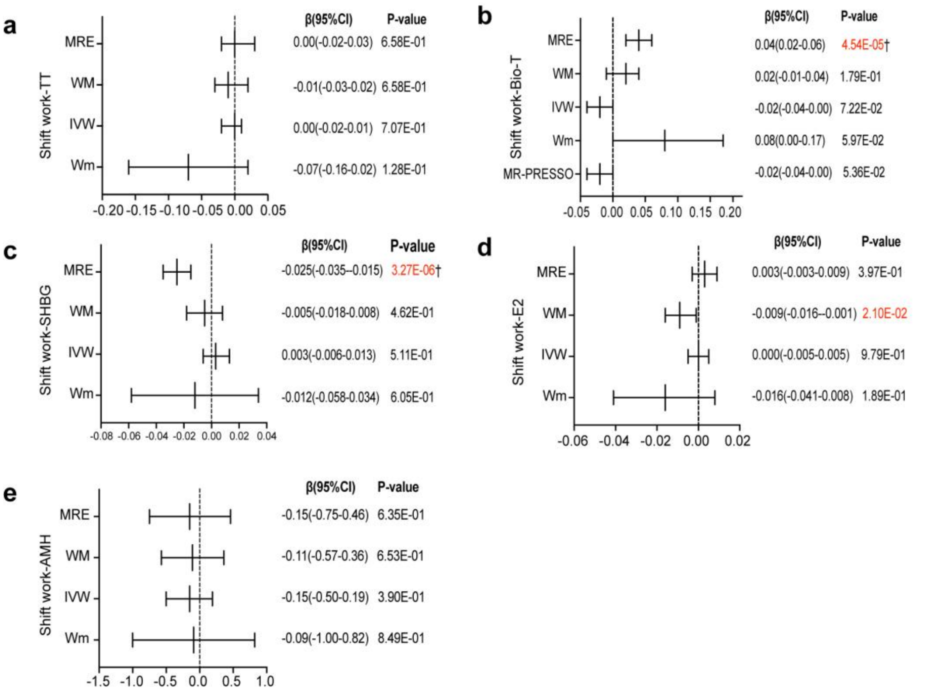

Supplementary Figure. 9: The causal inference results of female night shift and serum TT, Bio-T, SHBG, E_2 and AMH. (a) female night shift work-TT. **(b)** female night shift work-Bio-T. **(c)** female night shift work-SHBG. **(d)** female night shift work-E_2. **(e)** female night shift work-AMH. Bonferroni correction † P <0.05/42.

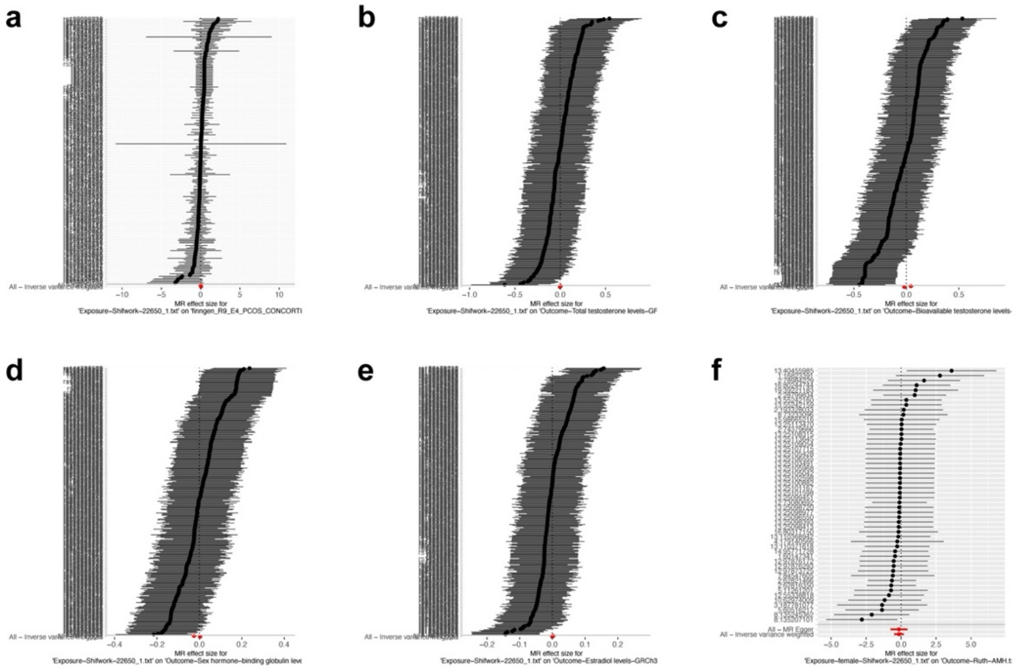

Supplementary Figure. 10: The forest plot of causal inference between female night shift and PCOS, serum TT, Bio-T, SHBG, E_2 and AMH. (a) female night shift work-PCOS**. (b)** female night shift work-TT. **(c)** female night shift work-Bio-T. **(d)** female night shift work-SHBG. **(e)** female night shift work-E_2. **(f)** female night shift work-AMH.

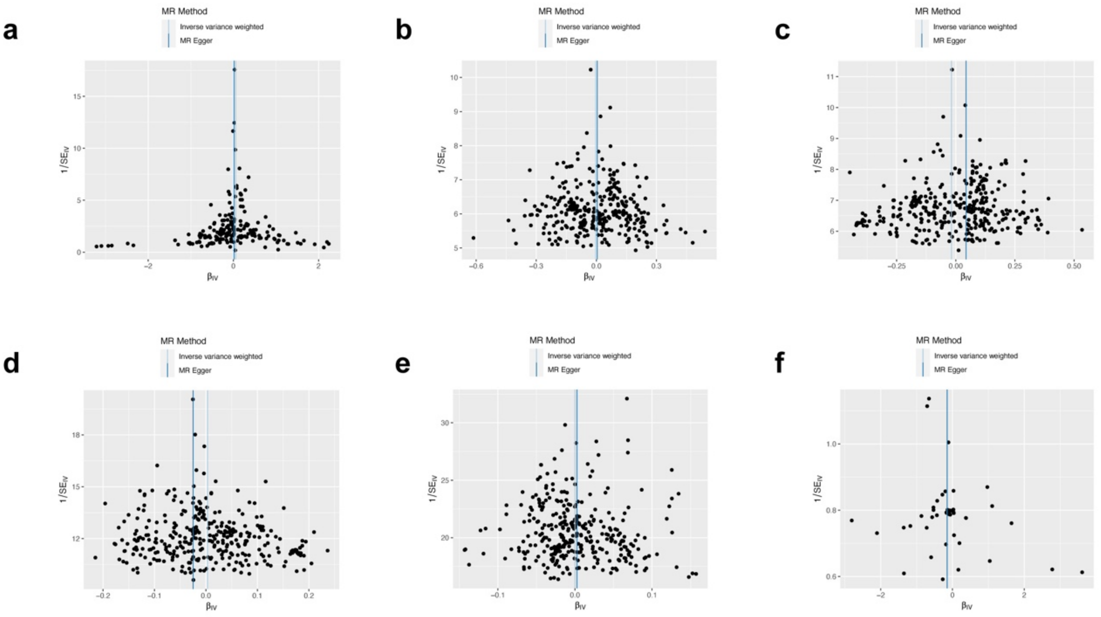

Supplementary Figure. 11: The funnel plot of causal inference between female night shift and PCOS, serum TT, Bio-T, SHBG, E_2 and AMH. (a) female night shift work-PCOS**. (b)** female night shift work-TT. **(c)** female night shift work-Bio-T. **(d)** female night shift work-SHBG. **(e)** female night shift work-E_2. **(f)** female night shift work-AMH.

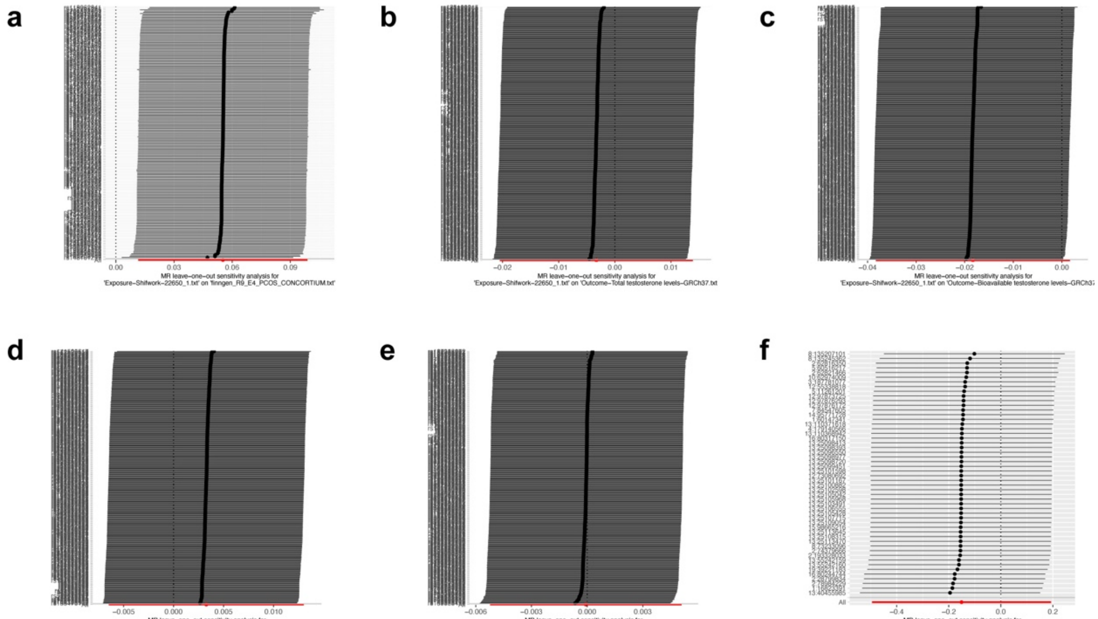

Supplementary Figure. 12: The leave-one-out plot of causal inference between female night shift and PCOS, serum TT, Bio-T, SHBG, E_2 and AMH. (a) female night shift work-PCOS**. (b)** female night shift work-TT. **(c)** female night shift work-Bio-T. **(d)** female night shift work-SHBG. **(e)** female night shift work-E_2. **(f)** female night shift work-AMH.

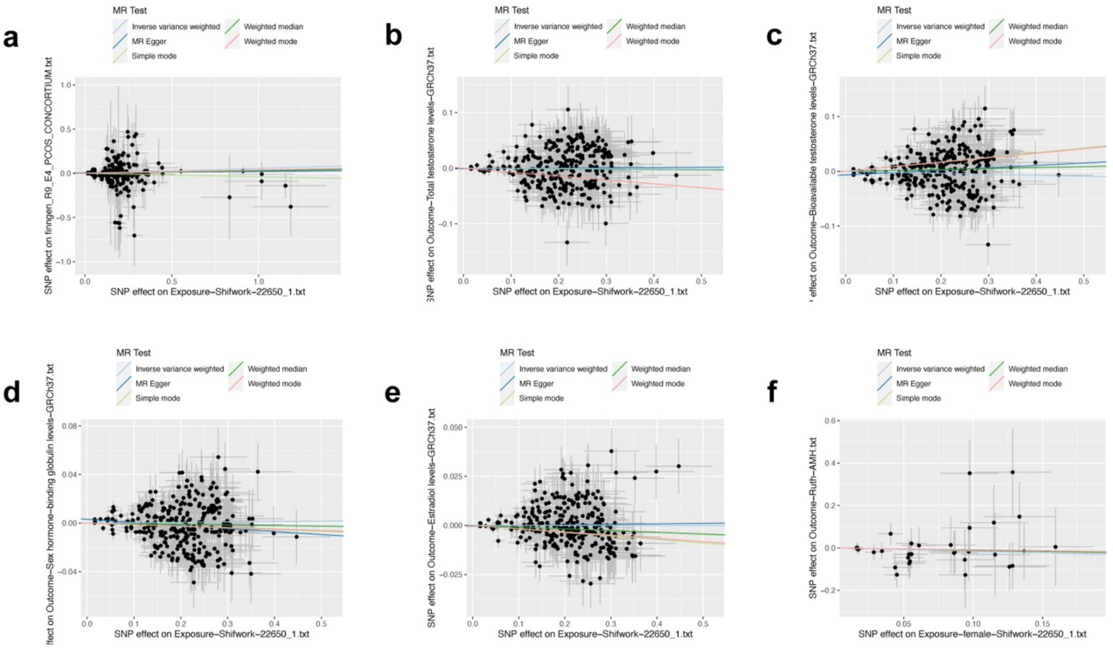

Supplementary Figure. 13: The scatter plot of causal inference between female night shift and PCOS, serum TT, Bio-T, SHBG, E_2 and AMH. (a) female night shift work-PCOS**. (b)** female night shift work-TT. **(c)** female night shift work-Bio-T. **(d)** female night shift work-SHBG. **(e)** female night shift work-E_2. **(f)** female night shift work-AMH.

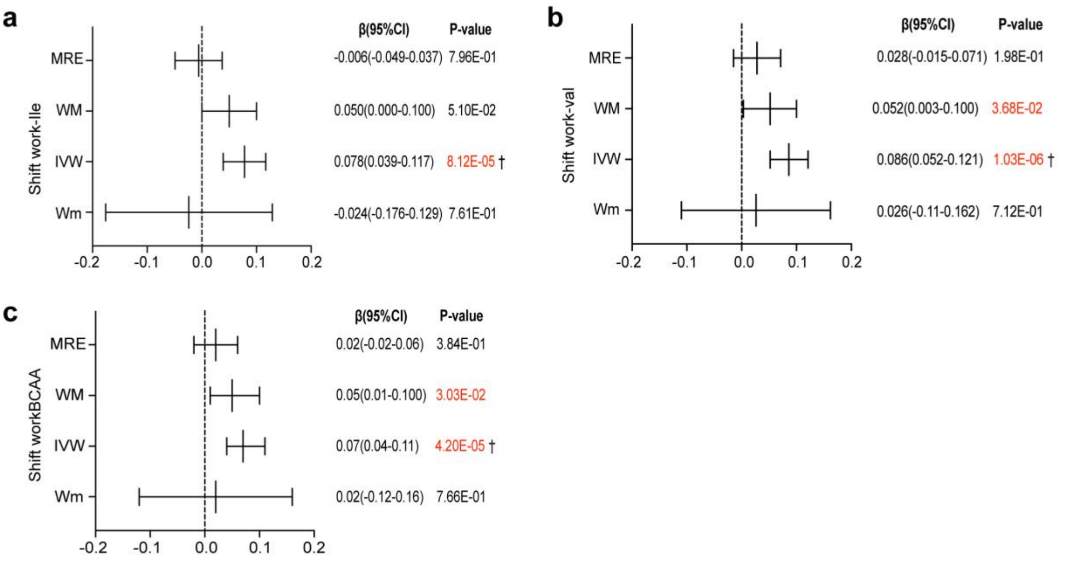

Supplementary Figure. 14: The causal inference results of female night shift and serum level of isoleucine, valine and BCAA. (a) female night shift work-isoleucine. **(b)** female night shift work-valine. (**c)** female night shift work-BCAA. † P <0.05/42.

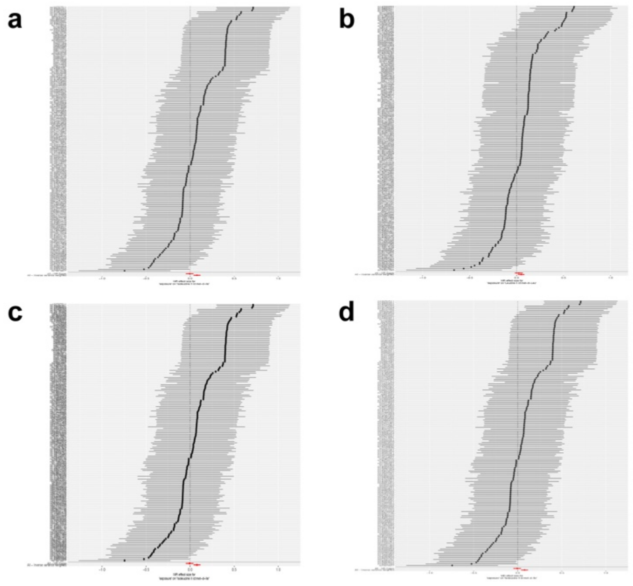

Supplementary Figure. 15: The forest plot of causal inference results of female night shift and serum level of leucine, isoleucine, valine and BCAA. (a) female night shift work-leucine. **(b)** female night shift work-isoleucine. **(c)** female night shift work-valine. (**d)** female night shift work-BCAA.

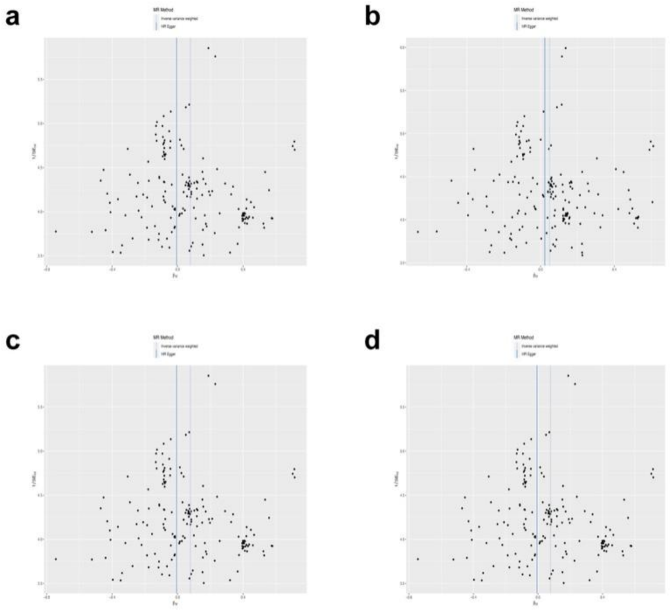

Supplementary Figure. 16: The funnel plot of causal inference results of female night shift and serum level of leucine, isoleucine, valine and BCAA. (a) female night shift work-leucine. **(b)** female night shift work-isoleucine. **(c)** female night shift work-valine. (**d)** female night shift work-BCAA.

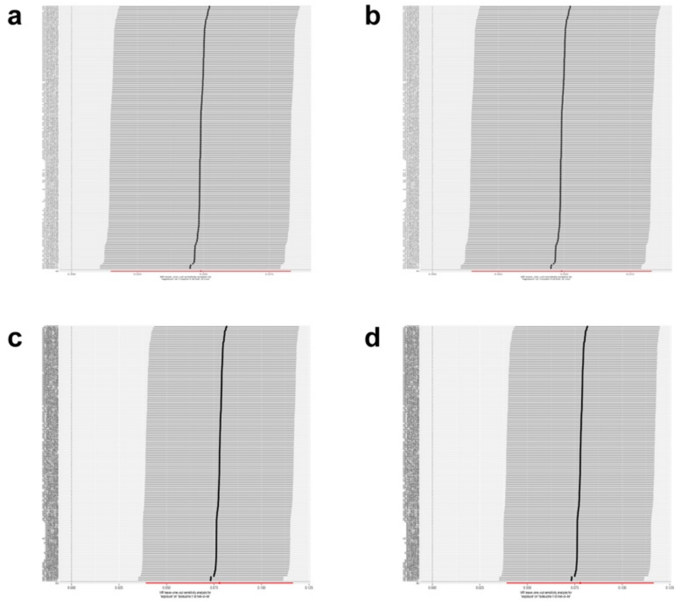

Supplementary Figure.17: The leave-one-out plot of causal inference results of female night shift and serum level of leucine, isoleucine, valine and BCAA. **(a)** female night shift work-leucine. **(b)** female night shift work-isoleucine. **(c)** female night shift work-valine. (**d)** female night shift work-BCAA.

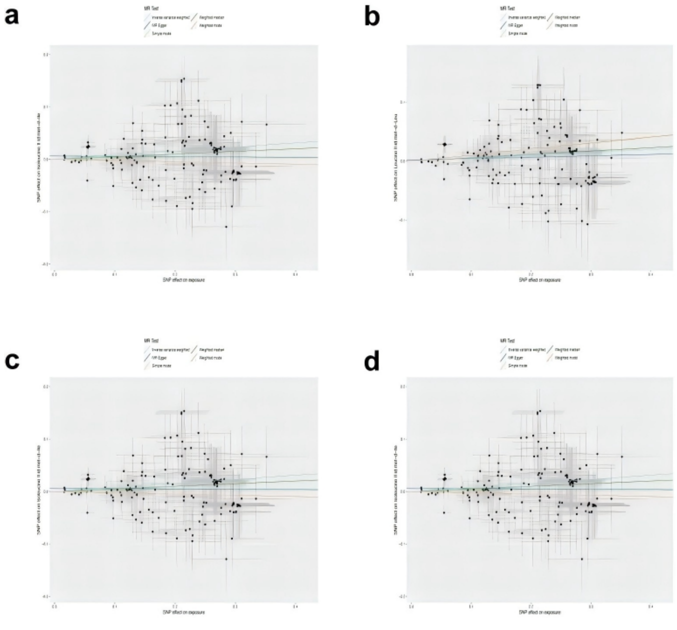

Supplementary Figure.18: The scatter plot of causal inference results of female night shift and serum level of leucine, isoleucine, valine and BCAA. **(a)** female night shift work-leucine. **(b)** female night shift work-isoleucine. **(c)** female night shift work-valine. (**d)** female night shift work-BCAA.

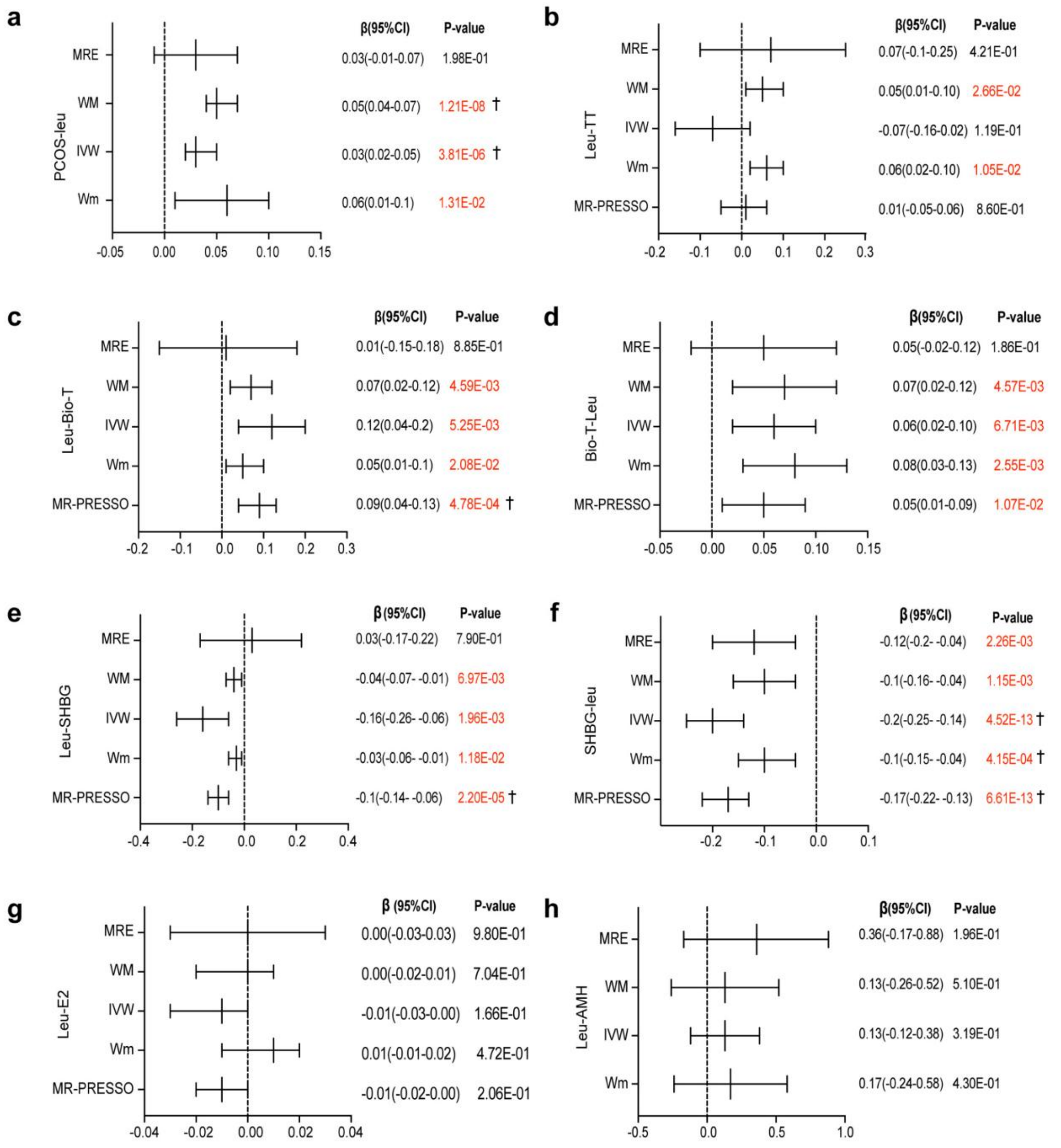

Supplementary Figure.19: The causal inference results between serum leucine level, PCOS and sex hormone level. (a) The reverse causal inference results of PCOS on serum leucine level. **(b)** The causal inference results of serum leucine level on TT. **(c-d)** The results of forward and reverse causal inference between serum leucine level and Bio-T. **(e-f)** The results of forward and reverse causal inference between serum leucine level and SHBG. **(g-h)** The causal inference results of serum leucine level and serum E_2, AMH.† P <0.05/42.

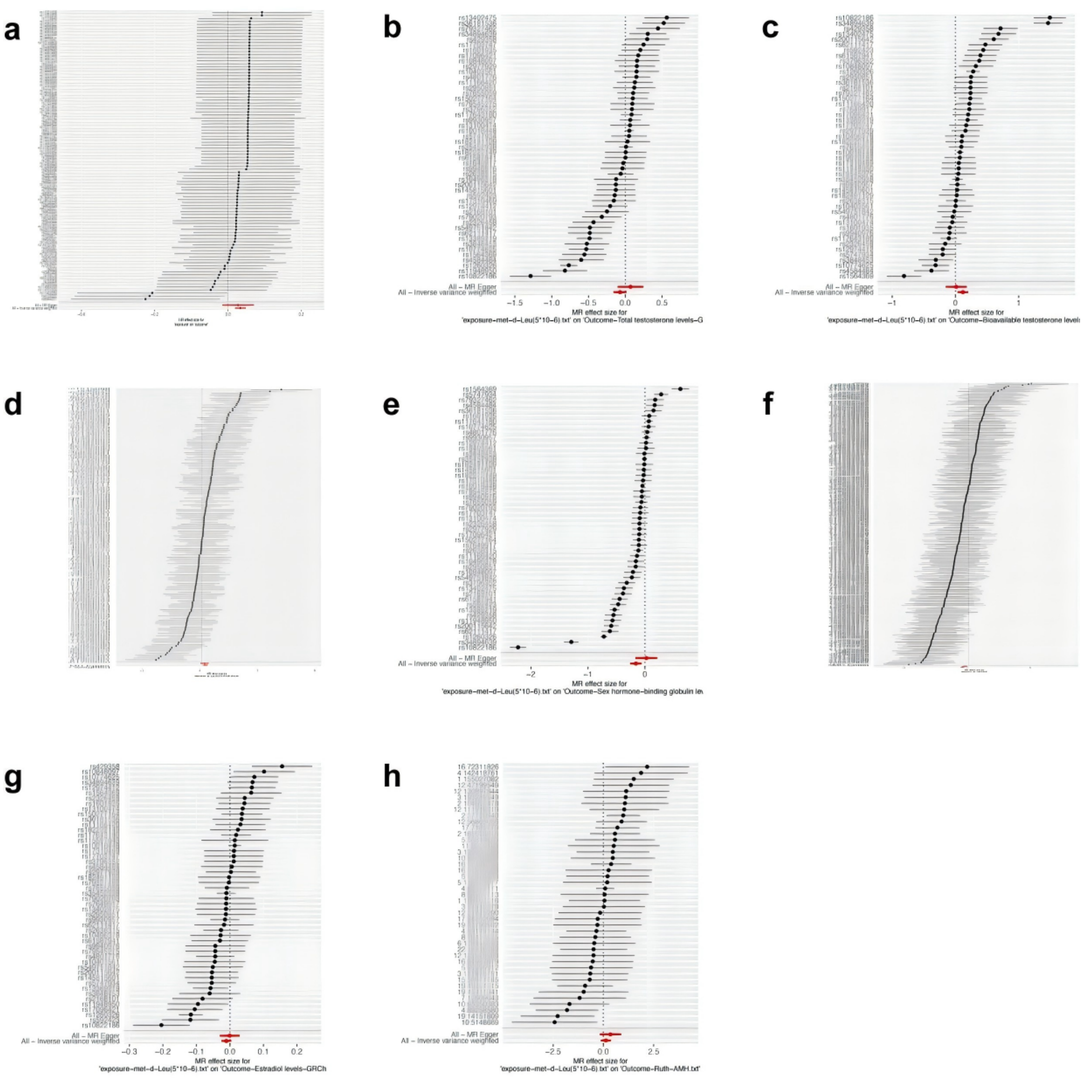

Supplementary Figure.20: The forest plots of causal inference results between serum leucine level, PCOS and sex hormone level. (a) PCOS-leucine. **(b)** leucine-TT. **(c)** leucine-Bio-T. **(d)** Bio-T- leucine. **(e)** leucine-SHBG. **(f)** SHBG-leucine. **(g)** leucine-E_2. **(h)** leucine-AMH.

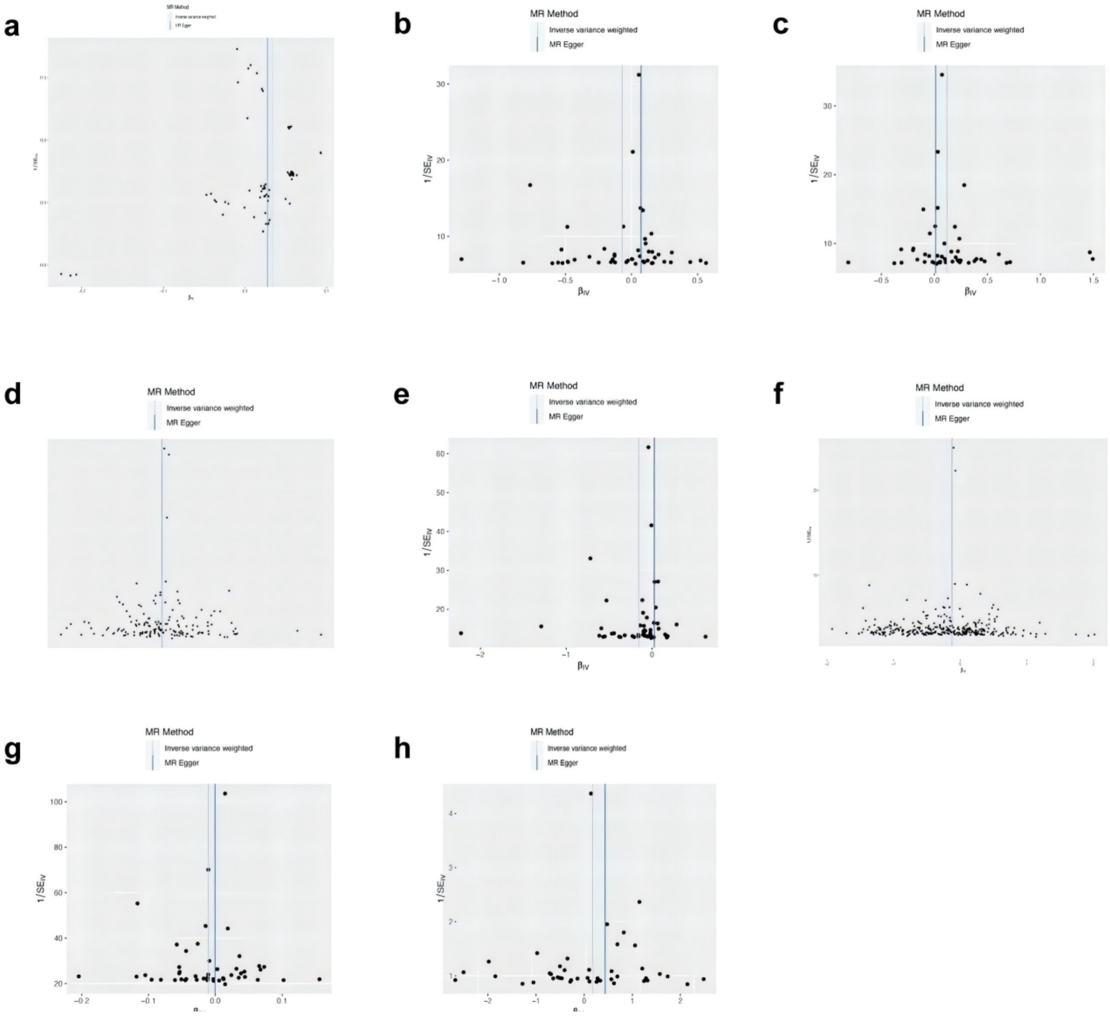

Supplementary Figure.21: The funnel plots of causal inference results between serum leucine level, PCOS and sex hormone level. (a) PCOS-leucine. **(b)** leucine-TT. **(c)** leucine-Bio-T. **(d)** Bio-T- leucine. **(e)** leucine-SHBG. **(f)** SHBG-leucine. **(g)** leucine-E_2. **(h)** leucine-AMH.

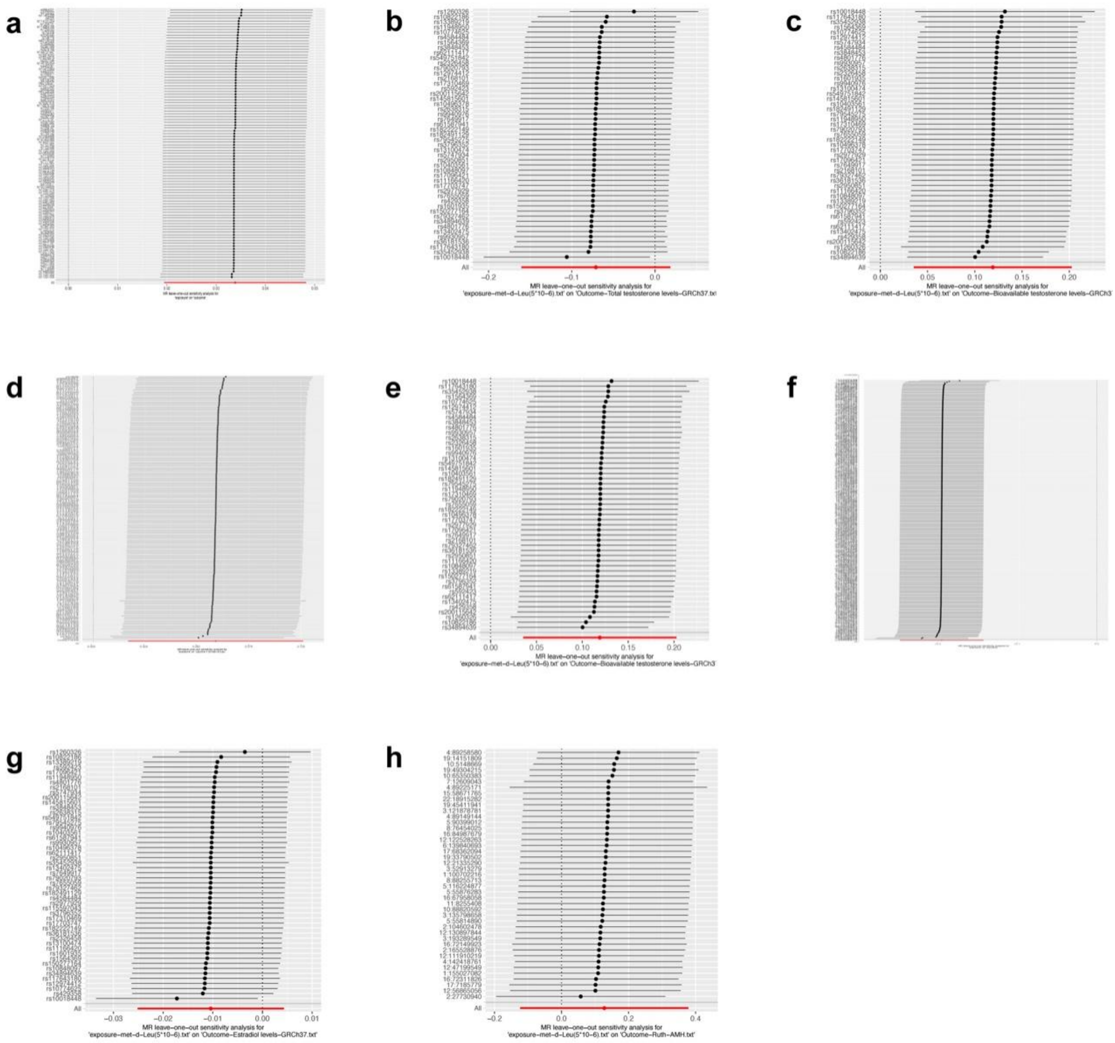

Supplementary Figure.22: The leave-one-out plots of causal inference results between serum leucine level, PCOS and sex hormone level. (a) PCOS-leucine. **(b)** leucine-TT. **(c)** leucine-Bio-T. **(d)** Bio-T- leucine. **(e)** leucine-SHBG. **(f)** SHBG-leucine. **(g)** leucine-E_2. **(h)** leucine-AMH.

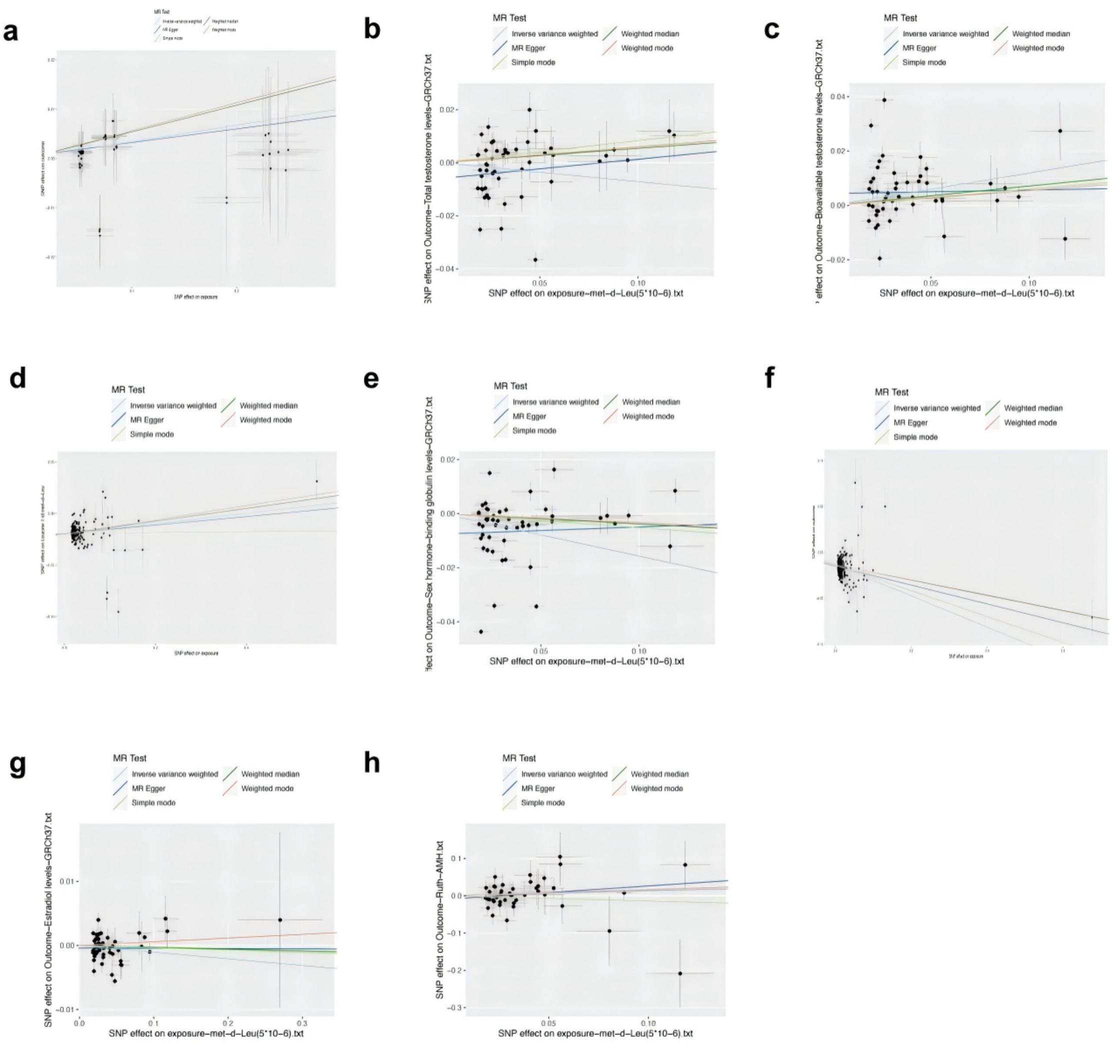

Supplementary Figure.23: The scatter plots of causal inference results between serum leucine level, PCOS and sex hormone level. **(a)** PCOS-leucine. **(b)** leucine-TT. **(c)** leucine-Bio-T. **(d)** Bio-T- leucine. **(e)** leucine-SHBG. **(f)** SHBG-leucine. **(g)** leucine-E_2. **(h)** leucine-AMH.

Supplementary methods

Estrous cycle determination

Vaginal smears were conducted on the NL, CL and CN groups of rats at a consistent time each day. The stage of the estrous cycle was determined through microscopic analysis of the predominant cell types in the vaginal smears. Proestrus: Characterized by round nucleated epithelial cells; Estrus: Dominated by keratinized squamous epithelial cells; Metestrus: Composed of epithelial cells and leukocytes; Diestrus: Featured nucleated epithelial cells with a predominance of leukocytes.

Determination of hormone and biochemical index in rats

The serum levels of corticosterone, total testosterone (TT) (Shanghai mlbio, ML059506), follicle stimulating hormone (FSH) (Qzkndbio, SU-B30597), luteinizing hormone (LH) (Qzkndbio, SU-B30623), and anti-müllerian hormone (AMH) (Qzkndbio, SU-B30802) were determined using ELISA kits, following the manufacturer's instructions. For lipid profile analysis, serum samples were assessed for triglycerides (TG, GPO-PAP method), total cholesterol (TC, GPO-PAP method), high-density lipoprotein (HDL-C, direct method-selective inhibition method), and low-density lipoprotein (LDL-C, direct method-surfactant clearance method) using the LW C400 automatic biochemical analyzer, also per the manufacturer's instructions. All kits were provided by Ningbo PuRuiBai Biological Technology Co., Ltd. Prior to each insulin tolerance test (ITT) or glucose tolerance test (GTT), the mice were fasted for 4-6 h or overnight (12-16 h), and their basal blood glucose levels were measured. Subsequently, the mice received an intraperitoneal injection of insulin (1.0 U/kg) or glucose (1.2 g/kg). Blood glucose levels were measured at 30, 60, 90, and 120 min following insulin injection. The area under the curve (AUC) of the response curve was then calculated to evaluate the glycemic response.

Body composition analysis

The body composition analysis was conducted using a low-field NMR awake small animal body composition analyzer (Suzhou Newman Analytical Instruments Co.,

LTD). Following an 8 h fast, the rats were positioned with the bottom end of the outer test cannula inserted, and the inner cannula was fixed to confine the animal within the test cylinder. The test cylinder was then inserted into the analyzer and activated via computer command. Data collected included measurements for: animal fat (g), muscle (g), fat content (%), muscle content (%), free water content (%). This approach allows for accurate assessment of body composition in a non-invasive manner.

Collection of cumulus oocyte complexes (COCs)

The rats in the NL, CL and CN groups were administered a single subcutaneous injection of 50 IU of pregnant mare's serum gonadotropin (PMSG) for 48 h, followed by 50 IU of hCG for 16 h. After this treatment, the rats were euthanized, and the ovaries along with the fallopian tubes were collected in a 35 mm petri dish containing 0.5 mL of pre-warmed culture medium-2. Using a stereomicroscope, the fallopian tube was punctured and squeezed with a 1 mL needle to collect cumulus-oocyte complexes (COCs) from the ampulla. All granulosa cells (GCs) were partially denuded by manual pipetting with 0.01% hyaluronidase. Finally, the number of metaphase II (MII) oocytes was counted to assess oocyte quality and maturation.

Ovarian morphology and follicle count

The ovaries were promptly collected, cleared of adipose tissue, fixed in 4% paraformaldehyde, dehydrated in 70% ethanol, and embedded in paraffin. Subsequently, the ovaries were sectioned into 5 μm thick slices and stained with hematoxylin and eosin (Core facilities，Zhejiang university school of medicine). Follicles were enumerated in maximum cross-section and classified into different developmental stages according to established standards. The follicle count was determined through histopathological examination of ovarian tissues from model rats. Using microscopic evaluation, we quantified follicles at different developmental

stages, including primordial, primary, secondary, antral, Graafian follicles, cyst and corpus luteum. Folliculogenesis was assessed by enumerating developing follicles in ovarian wedge sections under microscopic observation.

Serum untargeted metabolomics sequencing

The service was provided by iProteome Biotechnology Co., Ltd (Shanghai, China).

1. Sample extraction

1.1 The serum is thawed at 4 °C environments, and then 50 μL is added to 200 μL of pre-cooled methanol: acetonitrile: water (2:2:1) solution. It is vortexed for 30 s and placed at -20 °C for 1 h, followed by centrifugation for 10 min at 4 °C and 14000 g.

1.2 The liquid above is transferred to the EP tube and vacuum dried in a -80 °C refrigerator. Before the machine is officially started, the same sampling steps are required for quality control purposes, which are used to detect the state of the instrument, balance the chromatography-mass spectrometry system, and evaluate the stability of the entire experimental system.

2. Chromatographic conditions

2.1 Positive ion mode (POS): mobile phase A (10 mM ammonium acetate; Acetonitrile: water 95:5; with 0.1 % formic acid); Mobile phase B (10 mM ammonium acetate; Acetonitrile: water 50:50; with 0.1 % formic acid).

2.2 Negative ion mode (NEG): mobile phase A (10 mM ammonium acetate; Acetonitrile: water 95:5; pH adjusted to 8.0 with ammonia). Mobile phase B (10 mM ammonium acetate; Acetonitrile: water 50:50; pH adjusted to 8.0 with ammonia).

2.3 The samples are separated using Nexera UHPLC LC-30A liquid system and analyzed on a Q Exactive HF-X mass spectrometer.

GCs targeted amino acid metabolomics

The service was provided by iProteome Biotechnology Co., Ltd (Shanghai, China).

1. Standard configuration

1.1 Weigh the appropriate amount of amino acid standard and prepare the mother liquor with methanol or water. Take an appropriate amount of mother liquor to create a mixed standard, and dilute it into gradient concentrations with 10 % formic acid methanol-water (1:1) to make a working solution. Freeze the mother liquor and working fluid.

1.2 Weigh an appropriate amount of isotope standard (Trp-d3) and prepare the mother liquor with a concentration of 1000 ng/mL using 10% formic acid methanol-water (1:1).

2. Metabolite extraction

2.1 Add 300 μL of 10% formic acid solution-water to the sample, vortex for 1 min, then place the centrifuge tube containing samples in a 2 mL adapter, immerse it in liquid nitrogen for quick freezing for 5 min, and thaw at room temperature.

2.2 Place the centrifuge tube in a 2 mL adapter again, install it into the grinder, shake at 55 Hz for 1 min, then centrifuge the samples at 12000 rpm for 10 min at 4 °C.

2.3 Take the supernatant and add in100 μL of Trp-d3 internal standard (100 ng/mL). After swirling for 30 s, pass the supernatant through a 0.22 μm filter membrane, and add the filtrate to the detection flask.

3.Machine testing

3.1 Using ACQUITYUPLC ® BEHC18 chromatographic column (2.1x100 mm ,17μm, Waters, USA), use 5ul of sample, column temperature 35°C, mobile phaseA-50% methanol water (containing 0.1% formic acid), B-10% methanol water (containing 0.1% formic acid).

3.2 The gradient elution conditions are as follows:0-6.5 min, 90-70 % B; 6 -7 min ,70-0 % B; 7-14 min, 0 % B;14-14.5 min ,0-90 % B;14.5-17.5 min ,90 % B. Velocity is from 0-8.0 min and 0.3 mL/min ;8-17.5 min and 0.4 mL/min.

3.3 Mass spectrometry conditions: The electrospray ionization source was used in positive ion ionization mode. The ion source temperature was set at 500 °C, with an iron source voltage of 5500 V. The collision gas pressure was maintained at 4 psi, while the curtain gas pressure was set to 40 psi. Additionally, both the atomization gas and auxiliary gas were adjusted to 50 psi. These conditions were utilized for Multiple Reaction Monitoring (MRM) scan analysis.

4.Mass spectrometry data analysis

4.1 The raw data from mass spectrometry analysis are processed through Progenesis QI software for database retrieval to eventually obtain identification information of the sample.

4.2 Bioinformatics analysis method This includes protein cluster analysis, orthogonal partial least-squares discriminant analysis (OPLS-DA), and KEGG pathway annotation and enrichment.

The diagnosis, inclusion and exclusion criteria of PCOS patients

PCOS was mainly diagnosed based on the 2003 Rotterdam consensus criteria: (1) Oligomenorrhea or absence of menstruation; (2) Clinical hyperandrogenism (e.g., body hair Ferriman-Gallwey score ≥ 6, severe acne, etc.) or biochemical hyperandrogenism (total testosterone ≥ 0.57 ng/mL, 1.97 nmol/L); (3) The presence of polycystic ovary changes: from the perspective of ultrasound, the unilateral or bilateral ovaries have 12 or more follicles with a diameter of 2-9 mm, and/or the ovarian volume increases (> 10 mL); (4) To exclude other endocrine diseases, such as serum prolactin levels, androgen-secreting tumors, congenital adrenal cortex hyperplasia, Cushing's syndrome, thyroid disease, etc. The diagnostic criteria are that any two of (1), (2), and (3) must be fulfilled.

1.Inclusion Criteria for the PCOS Group

(1) Meet the diagnostic criteria for PCOS. (2) Infertile women of reproductive age. (3) Must have signed informed consent. (4) Subgroups: PCOS with hyperandrogenism, HA-PCOS (n=12); PCOS without hyperandrogenism, NA-PCOS (n=17), determined by total testosterone levels.

2. Inclusion Criteria for the Control Group

(1) Must have a normal menstrual cycle, without hyperandrogenism or ovarian lesions. (2) Infertile women of normal childbearing age. (3) Infertility should be caused by simple tubal factors or male factors. (4) Must not have taken oral contraceptives, metformin, or other drugs in the past three months. (5) Must have signed informed consent for scientific research.

3. Exclusion Criteria for the Control Group

(1) Presence of any ovarian lesions (including cysts, tumors, tuberculosis, etc.). (2) Previous surgical history such as ovarian cystectomy, unilateral oophorectomy, or salpingectomy. (3) Any uterine abnormalities (e.g., uterine fibroids, adenomyosis, tumors, etc.).

Primer List:

Gene	Forward Primer	Reverse Primer
Bmal1	ATTCCAGGGGGAACCAGA	GAAGGTGATGACCCTCTTATCCT
Rev-erbα	AATGTTCTGCTGGCATGTC	GAAGTCTTCCCAGATCTCCT
Rev-erbβ	TTGCAAAGGGAAGTGATTGG	TGGCATGCTCTCAGATGAG
Per1	GAGTTCTCACAGTTCATCTTCTG	GAGTTTGTACTCTTGCTGCTC
Per2	AGGTGAAGGCTAATGAGGAG	TGTAGGAAGGCACATCCAC
Per3	CTACAGTCAGAAAGTGTTGCC	CTTGAATCCTTCTGCGGTC
Roc-a	ATAGGACCAGCAGAAACCG	ACTTGACAGCATCTCGAGAC
Dbp	CAGAGGAGGAATTGAAGCC	ACTTCTCATCCTTCTGTTCC
Npas2	CACTACTACATCACCTACCACC	ATCTGCGTAACTGACCACC
Cry1	ACTGCTCTCAAGGAAGTGG	AGCTTCTTCCTTGCTTTAGTG
Cry2	CTTGTACAGAAAGGTGAAGAGG	GTATAGAAGAATTCTCGCCATAGG
Slc3a2	CTTCCTGGACAGCCTATGG	GAGGCTTACAGGACTTGAGG
Slc7a5	CCATCAAGGTGAATCTGGC	GTGTCTTCCAGAAGGACAC
Bcat1	AAGAATTAGAAGCTTCGACCG	GATCTTTGGCCCTAAACGTC
Ppm1k	CAAGGAGTATTGGAGATTTGGA	CGTGGTAGAGCTTAATCCTG
Dld	TGTGACAGTGATAGGTTCTGG	ATGCAGACTGTCTTAAAGCCT
Sestrin2	TTCCCAGGAGGAAATGGAG	TCCAGAATATCCGCTGAGG
Sestrin3	ACTTTGGGATTGTGGACTC	CATCTTCCCTGTCTTCCTG
Ulk1	GCTTACAGACTGCCATTGAC	TTAGTCTGCGTACCACCTG
Ulk2	CTCTCCACGGAGTTCTGAC	AAAGGAGCTGTAGTCTTAGTAGG
LC3-I	TACAAAGAGGAGCATCCGT	TTTCCACTATCACCGGGAC
LC3-II	ATAATTAGAAGGCGCCTGC	TTCAGAGATGGGTGTGGAC
p62	CATTAAAGAGAAGAAGGAGTGC	CATCACAAATCACGTTGGG
Stx17	CCCTCCTAGTAAATTCTCAGCA	CCTCTTCAACATTCACAGCA
Gapdh	AACTCCCATTCTTCCACCT	TTGTCATACCAGGAAATGAGC

Title: The Leucine-mTOR-Autophagy Axis in Granulosa Cells Mediates Circadian Disruption-induced Anovulation

Index

Supplementary Table 1. Base characteristic of PCOS patients.
Supplementary Table 2. Overview of exposure and outcomes GWAS.
Supplementary Table 3. The characteristics of female shift work associated SNPs.
Supplementary Table 4. The characteristics of PCOS associated SNPs.
Supplementary Table 5. The characteristics of serum leucine associated SNPs.
Supplementary Table 6. The characteristics of isoleucine associated SNPs.
Supplementary Table 7. The characteristics of valine associated SNPs.
Supplementary Table 8. The characteristics of BCAA associated SNPs.
Supplementary Table 9 Comparison of four different statistical methods for MR analysis evaluating the causal association of female shift work,BCAA, PCOS and sex hormones.
Supplementary Table 10.Heterogeneity test for each group
Supplementary Table 11.Summary for directional horizontal pleiotropy tests

Supplementary Table1. Base characteristic of PCOS patients.

Serum	Control (n=16)	PCOS-HA (n=12)	PCOS-NA (n=17)	Con vs. PCOS-HA	Con vs. PCOS-NA	PCOS-HA vs. PCOS-NA
Age(years)	28.69±3.84	29.83±2.08	29.94±2.01	ns	ns	ns
Height(m)	1.60±0.06	1.62±0.07	163.24±4.62	ns	ns	ns
Weight(kg)	57.81±6.85	62.53±8.99	64.43±9.37	ns	ns	ns
BMI(kg/m2)	22.52±2.32	23.98±3.9	24.17±3.28	ns	ns	ns
Cycle length(Days)	29.53±2.7	54.83±22.77	51.28±21.75	***	***	ns
AMH（ng/ml）	3.86±1.95	10.12±5.59	10.26±4.26	***	****	ns
FSH(IU/L)	7.42±1.92	6.64±1.22	6.89±2.05	ns	ns	ns
LH(IU/L)	4.43±1.39	10.05±5.4	10.16±6.3	**	**	ns
LH/FSH ratio	0.65±0.31	1.54±0.76	1.41±0.6	***	***	ns
E2(pmol/L)	139.19±57.54	212.08±133.69	180.35±93.72	ns	ns	ns
P(ng/ml)	1.84±0.9	2.89±2.03	1.87±2.05	ns	ns	ns
PRL(ng/ml)	12.66±5.26	19.24±22.06	13.33±5.12	ns	ns	ns
TT(ng/ml)	1.69±0.46	3.32±0.7	1.99±0.27	****	ns	***
AFC	10.94±6.1	20.2±6.81	17.38±7.44	**	ns	ns
BCAA(ng/mL)	52863.45±6712.3	63897.96±10922.85	67367.18±11652.67	*	***	ns
Isoleucine(ng/mL)	4796.35±758.59	6106.37±1388.28	6674.6±1381.57	*	***	ns
Leucine(ng/mL)	29938.07±3923.3	36081.5±5785.16	37699.19±6890.34	*	***	ns
Valine(ng/mL)	18129.02±2412.41	21710.1±4164.85	22993.38±3683.93	*	***	ns

Supplementary Table2. Overview of exposure and outcomes GWAS.

	Consortium	(Case/Control) Samplesize	PMID
Exposure			
Chronotype（Morning person）	UK Biobank and 23andMe participants	697,828	30696823
Night Shift Work	UK Biobank	25,697	-
Outcome			
Total Testosterone (TT)	UK biobank	230,454	32042192
Bioavailable Testosterone (Bio-T)	UK biobank	188,507	32042192
Sex Hormone-Binding Globulin (SHBG)	UK biobank	189,473	32042192
Estradiol (E_2)	UK biobank	163,985	34255042
Anti-Mullerian Hormone (AMH)	Genome-Wide Meta-Analysis	3,344	30649302
Polycystic Ovaries Syndrome (PCOS)	Finn Gen	（31,548/179,322）210,870	-
BCAA(Ile,Leu,Val)	IEU open GWAS	115,048	-

Supplementary Table 3. The characteristics of female shift work associated SNPs.

SNP	CHR	POS	EA(OA)	EAF	BETA	SE	PVAL
rs182771659	1	16823391	A(G)	0.009	0.128	0.027	2.76E-06
rs142173175	1	23233263	C(T)	0.000	78.088	16.125	1.30E-06
rs184046835	1	29821248	G(T)	0.003	0.215	0.045	2.06E-06
rs201948211	1	44423100	T(C)	0.000	0.927	0.169	4.18E-08
rs137933045	1	46870705	A(G)	0.000	1.139	0.237	1.50E-06
rs74642512	1	55307332	T(G)	0.000	1.069	0.210	3.44E-07
rs4912220	1	60147341	T(C)	1.433	0.017	0.004	4.64E-06
rs59558623	1	62993823	A(G)	0.000	103.955	22.150	2.72E-06
rs190651703	1	78010770	G(A)	0.005	0.163	0.033	9.54E-07
rs200383865	1	86241379	C(T)	0.000	1.002	0.190	1.43E-07
rs139563237	1	118495213	A(G)	0.001	0.590	0.106	3.01E-08
rs138781234	1	119281165	C(T)	0.003	0.228	0.048	2.31E-06
rs143204667	1	150204202	G(A)	0.000	232.038	47.021	8.14E-07
rs201080691	1	158449899	T(C)	0.000	0.900	0.183	9.50E-07
rs541423433	1	165374667	C(T)	0.004	0.188	0.041	3.91E-06
rs34558931	1	168073846	G(C)	0.000	92.768	17.158	6.55E-08
rs185822112	1	181311536	A(G)	0.002	0.244	0.053	4.73E-06
rs147120459	1	184006377	T(C)	0.000	11.439	1.854	7.03E-10
rs148161299	1	200779754	T(C)	0.005	0.182	0.037	8.95E-07
rs377459546	1	201046130	T(C)	0.000	240.956	46.987	2.97E-07
rs78394637	1	206681383	T(C)	0.000	0.948	0.200	2.28E-06
rs560248440	1	219642243	TAGA(T)	0.008	0.144	0.030	1.98E-06

rs559901403	1	222904724	A(G)	0.000	8.671	1.641	1.28E-07
rs141943965	1	234059875	T(G)	0.005	0.194	0.035	4.78E-08
rs139243289	1	234064743	G(C)	0.005	0.207	0.035	4.58E-09
rs762300599	1	243630459	A(G)	0.002	0.273	0.054	3.92E-07
rs553178512	1	243822050	C(G)	0.002	0.276	0.055	5.86E-07
rs189208711	1	244245698	G(A)	0.002	0.256	0.054	2.36E-06
rs552288222	1	245776651	C(T)	0.006	0.147	0.032	4.45E-06
rs529677146	1	245810671	A(G)	0.005	0.185	0.037	7.29E-07
rs546246315	2	563903	G(A)	0.003	0.229	0.047	1.06E-06
None	2	735675	T(C)	0.002	0.342	0.061	1.97E-08
rs191977422	2	18335946	G(A)	0.011	0.129	0.025	2.21E-07
rs139510234	2	28799834	A(G)	0.016	0.097	0.021	2.09E-06
rs77810069	2	29274947	G(T)	0.000	0.898	0.184	1.04E-06
rs536619881	2	30576001	G(A)	0.004	0.208	0.042	7.00E-07
rs532258959	2	34959010	T(A)	0.002	0.309	0.065	2.25E-06
rs542150941	2	60069316	C(T)	0.003	0.284	0.050	1.75E-08
rs192090238	2	62744681	G(T)	0.009	0.124	0.027	3.84E-06
rs147344642	2	62816350	T(C)	0.008	0.126	0.027	2.17E-06
rs139775992	2	62821466	G(C)	0.008	0.128	0.027	1.63E-06
rs546798284	2	65780406	A(G)	0.006	0.148	0.032	3.73E-06
rs200032522	2	69748053	A(T)	0.000	1.001	0.200	5.72E-07
rs181236626	2	70169470	A(G)	0.004	0.206	0.041	5.06E-07
rs35743342	2	71627630	A(G)	0.000	0.926	0.184	5.19E-07
rs146862644	2	72007987	A(G)	0.008	0.137	0.030	4.03E-06
rs574664732	2	73234717	A(T)	0.003	0.204	0.043	2.69E-06

rs139147648	2	74379666	A(C)	0.009	0.159	0.027	4.93E-09
rs149013592	2	74388510	G(C)	0.006	0.157	0.034	3.41E-06
rs138654657	2	74473131	GT(G)	0.012	0.111	0.023	2.23E-06
rs114260443	2	78984229	C(A)	0.085	-0.041	0.009	3.14E-06
rs138125296	2	103040730	G(A)	0.000	237.319	47.000	4.50E-07
rs11681047	2	106626184	C(A)	0.004	0.203	0.041	8.81E-07
rs554470047	2	106673597	A(G)	0.004	0.239	0.042	1.00E-08
rs184622489	2	110065914	T(G)	0.000	1.792	0.368	1.12E-06
rs568831353	2	115161105	A(G)	0.005	0.173	0.036	1.61E-06
rs576478758	2	119872340	A(G)	0.002	0.272	0.059	4.00E-06
rs550108395	2	124301241	C(A)	0.002	0.272	0.054	4.00E-07
rs183637661	2	136552345	T(C)	0.000	141.014	29.728	2.13E-06
rs150555097	2	168539184	T(C)	0.003	0.292	0.049	2.02E-09
rs542652804	2	191035099	T(A)	0.004	0.186	0.040	4.08E-06
rs139154277	2	193328033	T(C)	0.040	0.061	0.013	1.60E-06
rs548091080	2	193736512	A(T)	0.004	0.185	0.040	3.78E-06
rs189420137	2	196788366	T(C)	0.000	235.717	47.002	5.38E-07
rs527454545	2	222365798	T(C)	0.000	0.882	0.182	1.37E-06
rs550305812	2	231889307	G(A)	0.001	0.398	0.082	1.05E-06
rs574375534	2	231923663	A(AC)	0.002	0.329	0.063	2.13E-07
rs149239709	2	231953427	A(G)	0.001	0.447	0.073	7.21E-10
rs184686310	2	231981143	C(G)	0.001	0.311	0.068	4.22E-06
rs571755702	2	232069934	G(T)	0.001	0.352	0.065	6.50E-08
rs7559279	2	233321739	A(G)	0.000	1.006	0.184	4.32E-08
rs572258695	2	235389311	C(T)	0.002	0.248	0.053	3.49E-06

rs570295956	2	242313024	G(C)	0.006	0.192	0.036	9.64E-08
rs190688446	3	19821434	T(G)	0.008	0.157	0.030	2.04E-07
rs551621797	3	26341128	C(A)	0.003	0.203	0.043	2.07E-06
rs114577010	3	32495950	A(G)	0.000	0.866	0.182	1.86E-06
rs184059386	3	32968680	T(C)	0.004	0.192	0.039	6.22E-07
rs570208465	3	37365302	G(T)	0.000	238.246	46.988	4.03E-07
rs201593796	3	47933902	T(C)	0.000	0.930	0.187	6.33E-07
rs535245518	3	49140119	A(G)	0.000	0.919	0.184	6.23E-07
rs146278360	3	49927981	C(T)	0.000	0.931	0.191	1.13E-06
rs542545521	3	52557486	T(C)	0.000	39.112	7.837	6.10E-07
rs545612302	3	52563907	C(T)	0.000	1.997	0.400	6.06E-07
rs189874739	3	54028623	A(C)	0.007	0.137	0.030	4.06E-06
rs142188228	3	56403614	C(T)	0.004	0.206	0.040	3.45E-07
rs367685479	3	57431828	A(G)	0.000	238.758	46.986	3.80E-07
rs187000736	3	71637047	G(A)	0.006	0.162	0.033	1.06E-06
rs777313428	3	74931582	G(A)	0.003	0.256	0.052	7.43E-07
rs553833735	3	76774082	G(A)	0.003	0.249	0.051	9.86E-07
None	3	93745859	C(T)	0.002	0.247	0.053	2.56E-06
rs190912140	3	104279992	T(C)	0.004	0.191	0.041	3.21E-06
rs150554107	3	114998314	A(C)	0.002	0.282	0.060	2.27E-06
rs141434122	3	115195759	G(C)	0.002	0.255	0.053	1.91E-06
rs151315684	3	131095550	TTCTG(T)	0.000	13.876	2.661	1.87E-07
rs148060228	3	131100595	AC(A)	0.000	17.329	3.122	2.92E-08
rs142215026	3	133673940	A(C)	0.000	39.339	8.514	3.87E-06
rs141740200	3	138347997	T(C)	0.000	0.938	0.184	3.64E-07

rs527798214	3	142275489	T(TAAGTA)	0.003	0.257	0.047	4.20E-08
rs200279997	3	155485450	A(G)	0.000	0.930	0.188	8.02E-07
rs187876428	3	168080532	T(C)	0.003	0.230	0.048	1.67E-06
rs186301411	3	187781077	T(C)	0.016	0.094	0.019	9.43E-07
rs201850320	3	190995844	T(C)	0.005	0.178	0.035	3.84E-07
rs114423071	3	195491903	T(C)	0.000	0.851	0.183	3.48E-06
rs540478847	3	195511618	G(A)	0.000	1.082	0.232	3.09E-06
rs372341933	4	2910323	T(C)	0.000	0.785	0.171	4.64E-06
rs75929906	4	43421265	A(G)	0.002	0.299	0.062	1.67E-06
rs140029223	4	63724430	G(C)	0.005	0.195	0.034	1.01E-08
rs144404739	4	63852339	A(G)	0.007	0.150	0.031	1.27E-06
rs540973953	4	72611379	C(T)	0.002	0.267	0.058	4.43E-06
rs147726745	4	72672213	G(A)	0.002	0.265	0.055	1.44E-06
rs549604242	4	84414555	A(T)	0.004	0.180	0.038	3.05E-06
rs7698902	4	87656031	G(A)	0.000	0.959	0.184	1.86E-07
rs6812524	4	88902725	A(G)	0.001	0.567	0.103	3.42E-08
rs1483919149	4	88929173	CGAG(C)	0.000	0.633	0.128	7.71E-07
rs554800458	4	118575172	T(A)	0.002	0.296	0.063	2.35E-06
rs61999315	4	120192664	A(T)	0.000	0.934	0.183	3.34E-07
rs191522966	4	124119669	T(C)	0.003	0.206	0.045	4.33E-06
rs181650024	4	124150903	C(T)	0.003	0.247	0.050	9.56E-07
rs560333899	4	125487128	A(G)	0.007	0.157	0.031	3.89E-07
rs188825313	4	125984904	G(A)	0.003	0.225	0.047	1.58E-06
rs140979926	4	140216926	C(G)	0.001	0.398	0.079	4.16E-07
rs369571582	4	147788684	C(T)	0.000	1.018	0.209	1.19E-06

rs756158610	4	157937424	T(C)	0.002	0.280	0.061	4.63E-06
rs570605130	4	159636174	T(C)	0.004	0.173	0.038	4.05E-06
rs545842533	4	159671030	T(C)	0.004	0.191	0.039	1.32E-06
rs543926847	4	159800792	G(A)	0.004	0.198	0.040	1.02E-06
rs181305730	4	159913849	C(T)	0.004	0.200	0.041	1.10E-06
rs183100215	4	164096696	T(C)	0.003	0.299	0.048	5.72E-10
rs185250090	4	179140566	C(T)	0.011	0.116	0.025	2.67E-06
rs115564310	5	163461	A(G)	0.001	0.424	0.092	4.33E-06
rs75434344	5	1243850	A(T)	0.000	4.687	1.025	4.89E-06
rs62338527	5	11261201	T(C)	0.190	0.028	0.006	4.81E-06
rs146516902	5	28348391	G(T)	0.006	0.157	0.032	8.95E-07
rs150849493	5	28414749	C(T)	0.005	0.180	0.039	4.70E-06
rs59329886	5	39209149	A(G)	0.003	0.253	0.052	1.25E-06
rs115808605	5	57755750	A(C)	0.000	236.195	47.010	5.12E-07
rs79281622	5	60516217	A(G)	0.044	0.054	0.012	4.37E-06
rs201855456	5	79809566	T(G)	0.000	233.972	47.008	6.54E-07
rs76512279	5	111756091	T(G)	0.000	0.551	0.116	2.16E-06
rs186716820	5	113051515	G(A)	0.005	0.165	0.036	4.31E-06
rs140620979	5	114517429	C(T)	0.002	0.308	0.062	8.01E-07
rs565172192	5	116028800	G(C)	0.004	0.200	0.043	3.39E-06
rs115737081	5	131951692	C(T)	0.000	1.916	0.414	3.72E-06
rs376374088	5	140176667	A(G)	0.000	0.679	0.130	2.01E-07
rs184669211	5	143184366	T(C)	0.003	0.212	0.044	1.09E-06
rs189696401	5	144661271	A(G)	0.002	0.284	0.056	3.80E-07
rs370761304	5	149767515	A(C)	0.000	0.925	0.184	5.30E-07

rs138390114	5	157065428	C(G)	0.000	13.462	2.846	2.27E-06
rs182220520	5	157078720	T(G)	0.000	10.129	2.164	2.89E-06
rs192392710	5	179709087	A(G)	0.007	0.146	0.032	4.50E-06
rs186173232	5	179733265	A(G)	0.007	0.135	0.029	4.76E-06
rs75582401	6	31807343	T(C)	0.000	52.608	11.240	2.90E-06
rs6453563	6	72400667	A(T)	1.092	0.016	0.003	2.95E-06
rs191538139	6	101655420	T(C)	0.002	0.260	0.052	6.06E-07
rs180802632	6	101655451	G(A)	0.002	0.267	0.055	1.14E-06
rs188185530	6	119348286	G(A)	0.002	0.346	0.064	8.12E-08
rs193203852	6	119372290	G(T)	0.002	0.353	0.066	7.86E-08
rs573671031	6	120585003	G(A)	0.002	0.334	0.060	2.91E-08
rs560134614	6	120676799	C(T)	0.002	0.347	0.064	5.07E-08
rs140617448	6	139233926	A(G)	0.000	73.673	15.670	2.61E-06
rs191577227	6	139644963	T(C)	0.003	0.239	0.050	2.16E-06
rs555248393	6	140230656	C(G)	0.007	0.151	0.031	7.95E-07
rs181640449	6	140230664	T(G)	0.006	0.146	0.031	3.28E-06
rs79405730	6	140606670	A(G)	0.009	0.121	0.026	2.51E-06
rs181149346	6	140774204	T(A)	0.007	0.144	0.030	1.48E-06
rs146644743	6	140879192	G(A)	0.009	0.122	0.026	1.91E-06
rs139128294	6	141582914	G(A)	0.006	0.167	0.032	2.31E-07
rs186097461	6	141901458	C(G)	0.002	0.250	0.054	3.24E-06
rs529110595	6	141964227	C(T)	0.003	0.252	0.050	3.96E-07
rs755686467	6	141975923	A(AC)	0.003	0.254	0.047	5.24E-08
rs189706560	6	144194584	T(C)	0.002	0.311	0.061	3.11E-07
rs370464891	6	151151830	T(C)	0.000	124.729	25.127	7.00E-07

rs113884013	6	154593535	G(T)	0.005	0.181	0.036	6.61E-07
rs111812121	6	154604767	C(T)	0.006	0.161	0.035	4.25E-06
rs201873667	6	158540054	T(C)	0.000	1.018	0.191	9.89E-08
rs371956531	6	159184408	A(G)	0.000	0.906	0.184	7.99E-07
rs147001164	6	159398788	A(C)	0.000	0.892	0.183	1.10E-06
rs34617108	6	167427045	G(C)	0.000	39.626	8.654	4.72E-06
rs569072010	7	794376	C(A)	0.000	231.083	47.007	8.96E-07
rs966270553	7	7592555	T(TG)	0.000	0.942	0.154	9.53E-10
rs188864553	7	8010341	A(G)	0.005	0.163	0.035	2.57E-06
rs572988266	7	12965068	A(G)	0.002	0.294	0.060	8.46E-07
rs544429650	7	12965554	T(G)	0.002	0.297	0.059	4.20E-07
rs112664820	7	12967606	A(G)	0.002	0.284	0.058	1.09E-06
rs554079376	7	12973826	C(T)	0.002	0.306	0.059	1.78E-07
rs534502839	7	12974086	A(G)	0.002	0.301	0.057	1.44E-07
rs529216410	7	12974848	A(G)	0.002	0.297	0.058	3.58E-07
rs573996051	7	12978787	A(T)	0.002	0.306	0.057	8.80E-08
rs549437119	7	12979005	G(C)	0.002	0.276	0.057	1.19E-06
rs536073781	7	12979557	A(G)	0.002	0.297	0.058	3.72E-07
rs531440275	7	12981741	C(A)	0.002	0.291	0.057	3.77E-07
rs533894613	7	12982884	T(C)	0.002	0.296	0.059	4.44E-07
rs527741700	7	12983411	C(A)	0.002	0.297	0.058	3.68E-07
rs762620296	7	12984350	T(C)	0.002	0.308	0.058	1.28E-07
rs570776530	7	12988716	G(A)	0.002	0.295	0.058	3.29E-07
rs569663180	7	12988780	C(A)	0.002	0.311	0.059	1.28E-07
rs201310718	7	12991709	G(A)	0.002	0.292	0.058	4.10E-07

rs562644231	7	12992950	T(A)	0.002	0.301	0.058	1.68E-07
rs567751710	7	12993336	G(T)	0.002	0.304	0.058	1.49E-07
rs569903322	7	12994083	G(A)	0.002	0.306	0.059	1.96E-07
rs748418399	7	12995938	T(C)	0.002	0.297	0.058	3.13E-07
rs535233032	7	12996119	G(A)	0.002	0.303	0.059	3.02E-07
rs17167679	7	13978734	A(G)	0.012	0.119	0.025	1.51E-06
rs545366700	7	20489907	CATTT(C)	0.007	0.160	0.033	1.10E-06
rs188761431	7	46569503	T(C)	0.002	0.278	0.057	1.19E-06
rs182206648	7	73157367	T(G)	0.004	0.205	0.041	4.49E-07
rs76531289	7	84547605	T(G)	0.017	0.094	0.020	3.30E-06
rs538827948	7	87707326	A(G)	0.003	0.212	0.046	4.48E-06
rs561335434	7	100648702	C(G)	0.000	0.658	0.134	9.64E-07
rs370014497	7	121616897	C(G)	0.000	1.229	0.248	7.65E-07
rs2551020	7	133478601	A(C)	1.997	-0.214	0.047	4.67E-06
rs560118240	7	138987150	T(G)	0.004	0.210	0.044	1.87E-06
rs574111087	7	141080412	G(T)	0.003	0.249	0.051	1.28E-06
rs183564897	7	141920900	T(A)	0.000	1.046	0.205	3.32E-07
rs183005143	7	148010443	C(T)	0.002	0.245	0.053	3.32E-06
rs187374134	7	148017756	A(G)	0.003	0.247	0.051	1.04E-06
rs73726142	7	150174628	T(C)	0.000	7.496	1.318	1.33E-08
rs78465761	7	150741191	G(A)	0.000	20.611	4.504	4.79E-06
rs533811103	7	158889595	A(G)	0.000	10.727	2.321	3.87E-06
rs185299496	8	1143085	T(G)	0.004	0.178	0.039	4.90E-06
rs7017210	8	6479160	T(C)	0.000	0.907	0.169	7.55E-08
rs75101520	8	15381100	G(T)	0.005	0.183	0.037	7.01E-07

rs189555450	8	18591850	A(G)	0.002	0.301	0.060	4.63E-07
rs113766194	8	18683360	A(C)	0.003	0.223	0.048	2.93E-06
rs760161463	8	22731562	T(G)	0.002	0.294	0.053	3.16E-08
rs115376990	8	22864488	A(G)	0.000	52.402	9.591	4.76E-08
rs140868573	8	22901208	C(T)	0.002	0.285	0.060	1.76E-06
rs575334686	8	25084913	C(T)	0.003	0.216	0.042	3.72E-07
rs541606308	8	25104103	TCTTA(T)	0.003	0.214	0.043	6.03E-07
rs199560931	8	41559633	T(C)	0.000	0.988	0.180	4.28E-08
rs562534182	8	42059414	G(A)	0.005	0.178	0.038	3.68E-06
rs549441260	8	58264673	G(A)	0.002	0.266	0.052	3.71E-07
rs2229740	8	59502029	T(C)	0.000	1.013	0.192	1.40E-07
rs181211769	8	72602780	T(A)	0.002	0.276	0.052	9.07E-08
rs73686243	8	72607488	A(G)	0.003	0.218	0.046	2.67E-06
rs55717151	8	72608734	C(A)	0.003	0.223	0.047	2.62E-06
rs73686245	8	72611231	G(A)	0.003	0.212	0.046	4.36E-06
rs141315747	8	73233096	A(G)	0.020	0.084	0.018	3.24E-06
rs574774706	8	85323000	G(A)	0.002	0.281	0.052	8.82E-08
rs542426182	8	85599640	A(C)	0.002	0.278	0.053	1.24E-07
rs545547438	8	85773699	A(AAATT)	0.002	0.334	0.057	3.55E-09
rs140939214	8	89423765	C(T)	0.005	0.167	0.036	2.86E-06
rs142287256	8	89430709	G(A)	0.005	0.171	0.036	2.00E-06
rs543388864	8	90448567	G(C)	0.003	0.218	0.047	3.95E-06
rs768065763	8	100990223	C(CATA)	0.005	0.183	0.037	9.13E-07
rs146689235	8	109640585	G(A)	0.002	0.250	0.054	4.18E-06
rs184718161	8	118648596	A(T)	0.004	0.219	0.042	2.07E-07

rs74445276	8	135207101	A(G)	0.071	0.045	0.009	1.10E-06
rs138502935	8	135245362	A(G)	0.067	0.044	0.009	4.00E-06
rs538331455	8	142505504	A(G)	0.000	35.440	7.165	7.67E-07
rs549188427	8	145167028	A(T)	0.000	0.936	0.185	4.07E-07
rs572930896	9	36433697	G(A)	0.003	0.245	0.050	1.18E-06
rs565224701	9	36484797	C(T)	0.003	0.246	0.050	1.11E-06
rs143756117	9	74481093	A(G)	0.002	0.261	0.054	1.74E-06
rs79461339	9	75664888	C(T)	0.000	1.273	0.258	8.16E-07
rs557024552	9	84109891	A(C)	0.004	0.184	0.040	4.88E-06
rs567884248	9	94519643	G(A)	0.000	0.565	0.121	3.30E-06
rs530034502	9	99285901	T(C)	0.000	26.836	5.305	4.28E-07
rs150380925	9	99325064	C(T)	0.000	0.689	0.131	1.42E-07
rs62640054	9	117038033	T(C)	0.001	0.444	0.095	3.12E-06
rs572129150	9	127622717	C(G)	0.003	0.280	0.051	3.13E-08
rs552139261	9	127958889	TA(T)	0.004	0.192	0.041	3.29E-06
rs182003112	9	129842948	T(C)	0.004	0.199	0.042	1.70E-06
rs536946158	9	131026798	T(C)	0.003	0.221	0.048	4.06E-06
rs557431908	9	131052072	G(A)	0.003	0.223	0.048	3.29E-06
rs34691037	9	136029312	A(G)	0.001	0.489	0.087	1.81E-08
rs375584272	9	140328518	T(C)	0.000	0.660	0.131	4.43E-07
rs780027653	10	33475217	T(C)	0.000	1.204	0.210	1.00E-08
rs115162931	10	50724316	T(C)	0.000	0.909	0.185	8.56E-07
rs149200339	10	61732055	T(A)	0.004	0.204	0.041	5.52E-07
rs75876877	10	62974009	T(A)	0.042	0.054	0.012	4.38E-06
rs112144078	10	73046546	T(C)	0.001	0.356	0.070	3.28E-07

rs117904519	10	78156139	C(G)	0.008	0.140	0.028	8.07E-07
rs542877145	10	99185439	T(TC)	0.000	235.767	38.353	8.14E-10
rs3781365	10	105361871	T(C)	0.000	0.910	0.184	7.90E-07
rs551873691	10	116665956	G(C)	0.003	0.220	0.046	1.69E-06
rs79599682	10	121632039	T(C)	0.000	5.201	1.126	3.93E-06
rs2277242	10	124358420	A(C)	0.000	0.984	0.180	4.37E-08
rs145027455	10	124358621	C(T)	0.000	0.995	0.182	4.60E-08
rs61622276	10	124377636	T(C)	0.000	0.980	0.181	6.43E-08
rs59879555	10	124377660	T(C)	0.000	0.833	0.164	4.15E-07
rs532741043	10	124439956	C(A)	0.003	0.246	0.053	2.99E-06
rs74989047	10	125331762	C(T)	0.003	0.217	0.043	4.64E-07
rs77534112	10	125343192	C(G)	0.003	0.211	0.045	2.24E-06
rs114878148	10	125348874	G(A)	0.003	0.227	0.046	7.27E-07
rs147358912	10	125367519	G(A)	0.003	0.240	0.049	8.54E-07
rs144787672	10	126422841	T(C)	0.004	0.185	0.040	2.94E-06
rs146762280	10	129824037	G(C)	0.003	0.221	0.048	4.94E-06
rs192728686	10	132127302	A(G)	0.003	0.217	0.044	6.94E-07
rs563563171	11	1887768	T(C)	0.000	234.504	46.984	6.09E-07
rs551369423	11	2373513	T(C)	0.003	0.228	0.048	2.52E-06
rs199763905	11	4594581	T(C)	0.000	0.921	0.186	7.12E-07
None	11	4870101	C(T)	0.000	0.969	0.196	7.42E-07
rs188880331	11	40289050	T(C)	0.003	0.215	0.043	5.14E-07
rs143599553	11	40295819	T(C)	0.003	0.211	0.043	8.67E-07
rs190767511	11	40299885	T(C)	0.003	0.211	0.043	9.78E-07
rs80161108	11	40302844	T(C)	0.004	0.193	0.040	1.99E-06

rs145155883	11	40305353	T(C)	0.004	0.183	0.040	4.84E-06
rs35688434	11	41493863	TA(T)	0.013	0.126	0.024	1.63E-07
rs138878258	11	46911956	C(T)	0.000	0.727	0.134	5.80E-08
rs566025569	11	48682644	G(T)	0.003	0.229	0.049	2.56E-06
rs535287271	11	48685479	A(C)	0.002	0.262	0.056	2.53E-06
rs561577682	11	49316814	T(G)	0.002	0.272	0.059	3.51E-06
rs189064841	11	50563144	T(C)	0.003	0.238	0.048	8.30E-07
rs188289262	11	51216813	C(G)	0.002	0.279	0.057	9.35E-07
rs183995646	11	51312071	T(G)	0.003	0.226	0.048	2.52E-06
rs191336644	11	51374088	T(C)	0.003	0.227	0.047	1.71E-06
rs536095983	11	51374572	A(G)	0.003	0.224	0.047	2.28E-06
rs184067866	11	51491527	A(G)	0.002	0.249	0.054	3.87E-06
rs577264853	11	55352659	T(C)	0.002	0.246	0.054	4.87E-06
rs545904058	11	56156879	G(A)	0.003	0.229	0.048	1.85E-06
rs772761142	11	56193851	A(ATAT)	0.003	0.227	0.048	1.98E-06
rs146542991	11	56757183	C(T)	0.000	103.768	22.181	2.93E-06
rs185703456	11	59163821	G(C)	0.007	0.135	0.029	4.56E-06
rs530009948	11	59316892	G(A)	0.008	0.129	0.028	3.84E-06
rs571569718	11	59446565	T(C)	0.007	0.142	0.030	1.95E-06
rs147250208	11	64604225	T(C)	0.000	0.936	0.185	4.45E-07
rs4084085	11	64757664	T(C)	0.000	0.918	0.150	1.08E-09
rs34563314	11	64884936	A(G)	0.000	0.918	0.151	1.13E-09
rs192331810	11	68137112	T(C)	0.004	0.190	0.040	2.17E-06
rs113763456	11	68139800	T(G)	0.004	0.213	0.042	3.39E-07
rs530109034	11	68246775	T(A)	0.004	0.198	0.042	2.79E-06

rs573369840	11	68257094	C(G)	0.004	0.200	0.042	2.51E-06
rs572828494	11	70754024	C(T)	0.000	0.948	0.185	2.89E-07
rs529435929	11	78756279	G(C)	0.002	0.282	0.053	1.15E-07
rs551363291	11	80964761	C(T)	0.003	0.245	0.052	2.46E-06
rs540184165	11	90395889	T(A)	0.003	0.214	0.046	2.76E-06
rs575057772	11	90844798	T(C)	0.004	0.175	0.038	4.04E-06
rs571980031	11	91376662	CT(C)	0.012	0.109	0.024	4.34E-06
rs200763217	11	92534418	T(C)	0.000	235.778	47.015	5.38E-07
rs148013820	11	116729220	A(C)	0.000	238.241	46.993	4.04E-07
rs150221377	11	116798092	A(G)	0.000	143.611	29.723	1.37E-06
rs35411582	11	122720776	C(A)	0.000	0.915	0.184	6.91E-07
rs35561350	11	122726491	A(G)	0.000	0.930	0.193	1.49E-06
rs115562527	11	123024017	C(A)	0.003	0.239	0.052	4.11E-06
rs529413456	11	126645600	C(T)	0.002	0.347	0.061	1.64E-08
rs182061091	11	127494030	C(G)	0.006	0.145	0.031	2.95E-06
rs187358272	11	130256789	G(A)	0.003	0.210	0.043	9.27E-07
rs182592519	11	132819609	G(T)	0.003	0.220	0.046	2.25E-06
rs56981471	12	6120958	A(G)	0.000	0.766	0.166	4.10E-06
rs61749078	12	9013771	T(C)	0.000	69.029	14.867	3.47E-06
rs116751107	12	9147759	T(C)	0.000	8.139	1.593	3.26E-07
rs747831456	12	10503795	C(T)	0.002	0.311	0.063	9.84E-07
rs371894989	12	10782151	C(T)	0.001	0.447	0.094	2.02E-06
rs149408382	12	22063868	A(G)	0.000	10.010	2.189	4.88E-06
rs139956964	12	28935707	G(C)	0.003	0.250	0.047	8.69E-08
rs115853420	12	29904621	C(G)	0.000	0.898	0.183	1.00E-06

rs139268517	12	43788475	T(C)	0.002	0.292	0.059	8.35E-07
rs144754830	12	55338818	A(G)	0.050	0.053	0.011	1.98E-06
rs187451644	12	55968557	A(G)	0.000	170.058	33.223	3.13E-07
rs371921611	12	56628738	T(C)	0.000	0.870	0.181	1.63E-06
rs375241196	12	68646383	T(G)	0.000	2.649	0.539	9.15E-07
rs141505617	12	73080692	G(A)	0.009	0.137	0.026	9.17E-08
rs192934342	12	73218719	G(A)	0.005	0.182	0.036	5.20E-07
rs140260981	12	73291996	A(G)	0.006	0.156	0.034	3.07E-06
rs11109058	12	97873725	C(A)	0.044	0.054	0.012	3.42E-06
rs58459135	12	97876172	T(C)	0.043	0.055	0.012	2.26E-06
rs58618155	12	97876293	A(G)	0.044	0.056	0.012	1.79E-06
rs182523654	12	97876555	A(T)	0.043	0.057	0.012	1.03E-06
rs144336599	12	97891969	AT(A)	0.042	0.056	0.012	3.06E-06
rs201437849	12	97901044	T(TTG)	0.037	0.067	0.013	4.95E-07
rs7299748	12	102113302	C(T)	0.000	0.627	0.137	4.48E-06
rs144203181	12	123019295	G(A)	0.000	89.398	18.433	1.25E-06
rs199729163	12	124821618	A(G)	0.000	232.732	46.998	7.45E-07
rs200226590	12	132335486	T(C)	0.001	0.461	0.097	1.79E-06
rs546321188	13	23878943	G(T)	0.004	0.211	0.042	5.51E-07
rs9511319	13	25096550	G(A)	1.210	0.017	0.003	1.68E-06
rs67261322	13	25097178	C(CA)	1.213	0.017	0.003	1.84E-06
rs2050881	13	25098393	T(G)	1.208	0.016	0.003	2.48E-06
rs2050882	13	25098413	T(C)	1.211	0.016	0.003	2.16E-06
rs34588519	13	25098632	TG(T)	1.209	0.017	0.003	1.62E-06
rs6490947	13	25098720	G(A)	1.209	0.017	0.003	1.70E-06

rs6490948	13	25098977	C(A)	1.210	0.017	0.003	1.70E-06
rs2153605	13	25099451	G(A)	1.207	0.016	0.003	2.37E-06
rs5802287	13	25099828	CA(C)	1.205	0.016	0.003	3.19E-06
rs9507364	13	25100882	T(C)	1.209	0.016	0.003	1.94E-06
rs2862909	13	25101167	T(G)	1.209	0.016	0.003	1.94E-06
rs2862910	13	25101598	C(T)	1.207	0.016	0.003	2.74E-06
rs9551111	13	25102326	A(T)	1.209	0.016	0.003	3.89E-06
rs6490949	13	25102598	C(T)	1.209	0.016	0.003	1.95E-06
rs9511328	13	25103491	C(T)	1.209	0.016	0.003	1.94E-06
rs7321954	13	25103620	A(T)	1.207	0.016	0.003	2.75E-06
rs9511329	13	25105042	A(C)	1.209	0.016	0.003	1.91E-06
rs1590557	13	25105428	A(C)	1.209	0.016	0.003	1.93E-06
rs1590558	13	25105798	A(T)	1.209	0.016	0.003	1.92E-06
rs1579727	13	25105968	C(T)	1.207	0.016	0.003	2.75E-06
rs138083853	13	25106164	CAATGAAAATCAAA(C)	1.203	0.017	0.003	1.76E-06
rs2862911	13	25106555	C(A)	1.207	0.016	0.003	2.76E-06
rs2862912	13	25106557	T(C)	1.201	0.016	0.003	2.36E-06
rs2862913	13	25106558	T(A)	1.207	0.016	0.003	2.76E-06
rs1575844	13	25107715	C(A)	1.207	0.016	0.003	2.78E-06
rs4770705	13	25108315	T(C)	1.208	0.017	0.003	1.76E-06
rs1572896	13	25109054	A(G)	1.209	0.016	0.003	1.95E-06
rs4769364	13	25112187	C(T)	1.215	0.016	0.003	3.22E-06
rs7324236	13	25113470	G(A)	1.209	0.016	0.003	2.36E-06
rs7329566	13	25113645	C(T)	1.210	0.017	0.003	1.94E-06
rs2275939	13	25453420	G(A)	0.001	0.345	0.073	2.08E-06

rs61729909	13	25480145	A(G)	0.001	0.358	0.070	3.15E-07
rs116981543	13	25487096	C(T)	0.000	0.553	0.121	4.78E-06
rs772015709	13	27202882	T(C)	0.003	0.250	0.046	6.18E-08
rs183031593	13	40455985	C(G)	0.014	0.097	0.021	3.53E-06
rs9595522	13	47303011	G(A)	0.000	0.976	0.171	1.26E-08
rs561198053	13	49789175	C(T)	0.004	0.217	0.045	1.48E-06
rs116017864	13	51188874	T(A)	0.002	0.240	0.052	4.06E-06
rs146100710	13	51198351	G(T)	0.002	0.266	0.055	1.47E-06
rs74967981	13	54769948	A(G)	0.006	0.172	0.034	3.88E-07
rs187463763	13	55242159	A(G)	0.048	0.055	0.012	1.53E-06
rs193140334	13	55242160	A(C)	0.048	0.056	0.012	1.51E-06
rs148827339	13	93926306	A(G)	0.007	0.162	0.029	2.33E-08
rs118062841	13	93957136	C(A)	0.007	0.145	0.029	6.69E-07
rs576459644	13	99097195	T(C)	0.005	0.189	0.038	7.65E-07
rs573717053	13	103449232	T(A)	0.000	236.805	46.990	4.74E-07
rs545823085	13	105859444	G(A)	0.006	0.156	0.033	2.35E-06
rs149225506	13	110368942	G(A)	0.021	0.087	0.018	1.47E-06
rs142858783	13	110371618	A(T)	0.021	0.086	0.018	1.63E-06
rs2233412	14	35872989	A(G)	0.000	1.624	0.333	1.13E-06
rs189935247	14	44918486	T(C)	0.002	0.242	0.053	4.34E-06
rs34854642	14	58831567	G(A)	0.000	11.919	2.495	1.80E-06
rs76717297	14	69257781	C(T)	0.000	49.491	10.512	2.53E-06
rs184741686	14	75506718	T(C)	0.000	0.912	0.185	7.90E-07
rs548360391	14	78002248	G(C)	0.000	0.934	0.187	5.90E-07
rs17108255	14	79270039	A(G)	0.000	1.868	0.368	3.89E-07

rs75032153	14	95771728	C(T)	0.128	0.034	0.007	1.11E-06
rs575658165	14	99664352	G(C)	0.003	0.231	0.049	2.02E-06
rs185486466	14	99673862	T(C)	0.003	0.227	0.049	3.50E-06
rs566955887	14	101207878	A(G)	0.002	0.239	0.052	4.70E-06
rs112745231	15	42982708	G(A)	0.000	1.155	0.232	6.86E-07
rs151268951	15	49907355	T(C)	0.000	1.246	0.258	1.44E-06
rs540061015	15	57433851	T(C)	0.002	0.237	0.052	4.49E-06
rs181965656	15	62902276	T(C)	0.008	0.139	0.030	2.88E-06
rs539628058	15	74501772	A(G)	0.000	0.577	0.121	2.05E-06
rs183908636	15	85444442	G(A)	0.003	0.296	0.050	3.36E-09
rs142254313	15	89424828	A(G)	0.000	232.585	47.002	7.59E-07
rs11635459	15	98665216	T(G)	0.799	0.016	0.004	4.89E-06
rs572504542	15	99465449	A(G)	0.000	138.912	29.718	2.98E-06
rs143244233	16	538993	C(A)	0.000	0.932	0.184	4.22E-07
rs113290057	16	836187	T(C)	0.000	46.549	9.908	2.66E-06
rs112402703	16	836694	A(G)	0.000	46.549	9.908	2.66E-06
rs182138191	16	20060442	C(G)	0.003	0.227	0.045	5.56E-07
rs565725793	16	20800413	C(A)	0.003	0.228	0.049	3.69E-06
rs577519487	16	24341707	A(C)	0.002	0.263	0.053	8.31E-07
rs544972670	16	24341717	A(C)	0.002	0.265	0.054	7.36E-07
rs558528097	16	47627474	T(TAAGCTTTTTCCTGAAATTTAAGC)	0.000	233.996	47.018	6.56E-07
rs200126025	16	49557658	A(C)	0.000	0.922	0.184	5.64E-07
rs566574447	16	54684006	A(G)	0.008	0.139	0.029	1.66E-06
rs79648263	16	73431293	T(C)	0.007	0.130	0.028	4.65E-06
rs560161792	16	76192690	A(G)	0.010	0.122	0.025	1.46E-06

rs542259137	16	80244744	T(C)	0.010	0.133	0.026	2.28E-07
rs140400679	16	80317150	A(G)	0.015	0.097	0.020	1.84E-06
rs555315798	16	80997202	C(G)	0.003	0.213	0.046	3.51E-06
rs141351731	16	83106920	A(G)	0.012	0.112	0.024	3.46E-06
rs149738118	16	83739763	G(A)	0.008	0.133	0.028	2.34E-06
rs139633564	16	88874566	A(G)	0.000	4.733	0.949	6.22E-07
rs146025366	16	89348801	A(G)	0.004	0.193	0.040	1.75E-06
rs374705201	17	7158010	A(T)	0.000	233.380	47.007	6.97E-07
rs188303942	17	11940989	G(T)	0.004	0.206	0.039	1.39E-07
rs747147437	17	54449527	A(G)	0.003	0.216	0.047	4.68E-06
rs116630414	17	58156116	A(G)	0.000	0.950	0.184	2.53E-07
rs549355707	17	73624440	A(G)	0.001	0.554	0.108	3.07E-07
rs145976111	17	73738809	T(C)	0.004	0.196	0.036	6.14E-08
rs190896190	17	73741861	A(G)	0.004	0.213	0.040	7.30E-08
rs182333211	17	73741949	A(G)	0.004	0.199	0.040	8.22E-07
rs201255090	17	79682084	T(C)	0.000	139.029	29.738	2.97E-06
rs199916012	17	79682739	T(C)	0.000	139.029	29.738	2.97E-06
rs183868810	17	80246161	T(C)	0.008	0.145	0.029	7.56E-07
rs575821089	17	80265611	A(T)	0.007	0.144	0.031	2.98E-06
rs200019465	17	80606205	T(C)	0.000	86.266	15.244	1.56E-08
rs548955439	18	5431753	C(G)	0.003	0.237	0.050	2.11E-06
rs116232392	18	6908976	A(G)	0.001	0.438	0.095	4.46E-06
rs78279255	18	6947270	C(T)	0.001	0.778	0.116	2.32E-11
rs532285276	18	28847896	A(G)	0.005	0.165	0.035	2.95E-06
rs145568479	18	33767517	A(G)	0.000	17.321	3.788	4.86E-06

rs192929615	18	62519367	C(A)	0.005	0.174	0.037	3.38E-06
rs557389662	18	72335790	CTTA(C)	0.003	0.327	0.049	3.13E-11
rs547126619	18	72593044	T(C)	0.000	233.034	46.991	7.18E-07
rs140034619	18	72802503	A(G)	0.003	0.222	0.048	4.71E-06
rs145498618	18	74679836	G(C)	0.001	0.364	0.076	1.53E-06
rs113534319	19	501843	A(C)	0.000	7.315	1.444	4.14E-07
rs746236290	19	932574	G(A)	0.000	0.932	0.190	9.44E-07
rs529435184	19	4117465	T(C)	0.000	0.921	0.184	5.78E-07
rs186521961	19	6421882	T(C)	0.014	0.104	0.021	6.33E-07
rs113577546	19	6438510	T(C)	0.000	1.185	0.219	6.29E-08
rs746791777	19	17581347	G(A)	0.000	0.561	0.120	2.75E-06
rs201929387	19	22363821	C(G)	0.000	232.040	47.018	8.12E-07
rs190082118	19	32558030	C(T)	0.005	0.168	0.037	4.60E-06
rs79072548	19	39221183	G(T)	0.009	0.115	0.025	3.48E-06
rs147553354	19	44514859	T(C)	0.000	137.300	29.731	3.91E-06
rs373073593	19	48197588	G(A)	0.000	115.403	23.498	9.17E-07
rs202132729	19	48305786	A(G)	0.000	233.390	46.985	6.88E-07
rs147569295	19	50093627	A(G)	0.000	236.099	47.004	5.16E-07
rs187624868	19	52960101	C(T)	0.003	0.227	0.047	1.22E-06
rs566977650	19	53737736	T(C)	0.008	0.130	0.028	3.44E-06
rs188476103	19	58321334	A(G)	0.004	0.197	0.043	4.39E-06
rs147047403	20	4850572	T(G)	0.000	0.931	0.184	4.46E-07
rs148274374	20	16410504	G(C)	0.000	1.979	0.415	1.85E-06
rs539898837	20	30486203	T(C)	0.002	0.339	0.062	6.13E-08
rs201868448	20	39976188	T(G)	0.000	0.954	0.197	1.23E-06

rs189477591	20	47284983	C(T)	0.002	0.277	0.057	1.46E-06
rs573446668	20	47499548	A(G)	0.002	0.282	0.054	1.53E-07
rs560934598	20	47517360	A(C)	0.002	0.276	0.050	2.89E-08
rs186240834	20	47580570	C(A)	0.002	0.267	0.049	4.38E-08
rs531710094	20	47584376	G(A)	0.002	0.265	0.049	6.59E-08
rs76718800	20	47600162	G(A)	0.003	0.260	0.048	5.53E-08
rs192967050	20	47617386	C(G)	0.002	0.265	0.049	6.83E-08
rs547937535	20	47619662	A(G)	0.002	0.268	0.049	4.04E-08
rs188728835	20	47632151	T(C)	0.002	0.270	0.049	4.00E-08
rs79113581	20	47633391	G(T)	0.003	0.258	0.048	8.76E-08
rs187193013	20	47696782	G(T)	0.002	0.270	0.048	2.57E-08
rs190531251	20	47700451	A(G)	0.002	0.270	0.048	2.58E-08
rs75886438	20	47746535	A(G)	0.003	0.260	0.048	5.45E-08
rs562708331	20	47747097	C(T)	0.002	0.270	0.048	2.58E-08
rs535677465	20	47793574	A(G)	0.002	0.273	0.049	3.20E-08
rs143923594	20	47794148	A(G)	0.003	0.259	0.048	6.00E-08
rs373194550	20	47802744	A(G)	0.002	0.276	0.049	1.92E-08
rs371073056	20	47804626	A(AGGCCC)	0.002	0.267	0.048	3.38E-08
rs544947123	20	47815516	A(G)	0.002	0.269	0.048	2.76E-08
rs76619987	20	47898145	A(C)	0.002	0.268	0.048	3.17E-08
rs191713676	20	47940708	T(C)	0.002	0.265	0.049	5.56E-08
rs143601779	20	48155950	T(C)	0.002	0.246	0.050	8.84E-07
rs766817580	20	50629172	A(G)	0.004	0.195	0.042	4.03E-06
rs181934654	20	53150702	A(G)	0.009	0.131	0.027	1.65E-06
rs538939228	20	60885736	A(G)	0.000	48.086	10.136	2.12E-06

rs567108349	20	61360740	T(G)	0.006	0.161	0.034	2.44E-06
rs181426921	21	32647431	G(C)	0.003	0.228	0.047	1.06E-06
rs190645241	21	35128865	A(T)	0.003	0.229	0.049	2.42E-06
rs557717323	21	45196097	A(G)	0.000	234.174	46.985	6.32E-07
rs78240331	21	46876226	T(C)	0.000	0.902	0.184	1.02E-06
rs374142908	22	24037707	C(G)	0.000	0.926	0.185	5.31E-07
rs143554731	22	26707734	G(C)	0.000	0.935	0.184	4.05E-07
rs140927687	22	38636549	A(G)	0.012	0.111	0.024	4.00E-06
None	22	42416052	GGA(G)	2.000	-0.678	0.130	2.02E-07
rs552914564	22	44069475	A(G)	0.002	0.350	0.064	5.43E-08
rs147879775	22	50897692	T(C)	0.000	1.121	0.238	2.56E-06
rs374823102	22	50899091	A(G)	0.000	0.676	0.130	2.06E-07
rs16985634	X	9607103	G(A)	0.018	0.084	0.018	3.52E-06
rs763374678	X	47378939	C(T)	0.003	0.232	0.050	3.20E-06
rs181309104	X	112480536	T(C)	0.002	0.269	0.058	3.48E-06
rs192958939	X	115549787	C(T)	0.030	0.072	0.015	2.80E-06
rs147369153	X	119589270	A(G)	0.001	0.575	0.106	6.16E-08
rs1055829	X	122805568	C(T)	0.000	239.168	46.978	3.62E-07
rs143653619	X	129519281	T(C)	0.000	39.888	7.781	3.00E-07
rs782815072	X	144534915	A(G)	0.007	0.139	0.030	2.88E-06
rs377123063	X	150349618	T(C)	0.000	0.686	0.137	6.03E-07
rs111335722	X	152944494	A(T)	0.012	0.114	0.024	1.65E-06
rs74331897	X	153176208	C(G)	0.000	29.560	5.719	2.40E-07
rs192526096	X	153191583	T(C)	0.000	81.545	17.766	4.48E-06

Supplementary Table 4. The characteristics of PCOS associated SNPs.

SNP	CHR	POS	EA(OA)	EAF	BETA	SE	PVAL
rs7563201	2	43561780	G(A)	0.451	-0.108	0.017	3.68E-10
rs2178575	2	213391766	A(G)	0.151	0.166	0.022	3.34E-14
rs13164856	5	131813204	T(C)	0.729	0.124	0.019	1.45E-10
rs804279	8	11623889	T(A)	0.262	0.128	0.018	3.76E-12
rs10739076	9	5440589	A(C)	0.308	0.110	0.020	2.51E-08
rs7864171	9	97723266	A(G)	0.428	-0.093	0.017	2.95E-08
rs9696009	9	126619233	A(G)	0.068	0.202	0.031	7.96E-11
rs11031005	11	30226356	T(C)	0.854	-0.159	0.022	8.66E-13
rs11225154	11	102043240	A(G)	0.094	0.179	0.027	5.44E-11
rs1784692	11	113949232	A(G)	0.824	0.144	0.023	1.88E-10
rs2271194	12	56477694	T(A)	0.416	0.097	0.017	4.57E-09
rs1795379	12	75941042	T(C)	0.240	-0.117	0.020	1.81E-09
rs8043701	16	52375777	A(T)	0.815	-0.127	0.021	9.61E-10
rs853854	20	31420757	T(A)	0.499	-0.098	0.016	2.36E-09

Supplementary Table 5. The characteristics of serum leucine associated SNPs.

SNP	CHR	POS	EA(OA)	EAF	BETA	SE	PVAL
rs11166420	1	100702216	A(T)	0.904	-0.038	0.007	1.70E-08
rs145815601	1	155027082	T(G)	0.037	-0.056	0.011	1.60E-07
rs182491129	1	100426478	A(C)	0.012	-0.084	0.018	4.20E-06
rs549751842	2	296410	C(T)	0.320	-0.020	0.004	3.00E-06
rs13389219	2	165528876	T(C)	0.393	-0.032	0.004	2.30E-15
rs115597043	2	181353805	A(G)	0.001	-0.270	0.057	2.10E-06
rs200115642	2	227067191	A(T)	0.637	0.023	0.004	7.80E-08
rs1260326	2	27730940	C(T)	0.604	-0.048	0.004	3.60E-32
rs2422358	2	65231806	T(A)	0.436	0.025	0.004	1.40E-09
rs10496378	2	104602478	A(T)	0.136	-0.027	0.006	3.50E-06
rs13402475	2	3639909	G(C)	0.816	0.024	0.005	4.70E-06
rs3796352	3	52913279	T(C)	0.100	-0.031	0.007	2.10E-06
rs7649917	3	193289549	C(T)	0.227	0.023	0.005	1.50E-06
rs13100474	3	121878781	A(G)	0.206	0.026	0.005	1.90E-07
rs34894639	3	135798658	T(C)	0.232	-0.026	0.005	2.50E-08
rs150277164	4	89258580	A(G)	0.014	0.116	0.017	5.20E-12
rs7655059	4	89149144	G(C)	0.175	-0.034	0.005	1.10E-10
rs79545275	4	142418761	A(G)	0.028	-0.056	0.012	3.90E-06
rs10018448	4	89225171	G(A)	0.535	0.088	0.004	4.30E-108
rs17310469	5	116224877	G(A)	0.159	0.026	0.006	2.60E-06

rs61587941	5	55814890	A(G)	0.051	-0.045	0.009	5.90E-07
rs2950851	5	90399012	T(A)	0.096	-0.033	0.007	1.40E-06
rs11948950	5	55876283	A(G)	0.105	0.030	0.007	3.60E-06
rs592423	6	139840693	C(A)	0.554	-0.020	0.004	8.70E-07
rs182222149	7	12609043	G(T)	0.016	-0.080	0.016	8.00E-07
rs2977929	8	76454025	T(C)	0.197	0.028	0.005	1.80E-08
rs17703747	8	88255713	A(G)	0.040	-0.048	0.010	2.40E-06
rs17096421	10	88820592	T(A)	0.056	0.044	0.009	4.70E-07
rs10822186	10	65350383	G(A)	0.494	-0.020	0.004	7.20E-07
rs4584484	10	5148669	A(G)	0.218	0.022	0.005	4.40E-06
rs2168101	11	8255408	A(C)	0.308	-0.020	0.004	3.80E-06
rs1564369	12	21335290	G(T)	0.178	0.024	0.005	3.70E-06
rs79020793	12	47199549	A(G)	0.068	0.041	0.008	2.30E-07
rs10774625	12	111910219	G(A)	0.503	-0.023	0.004	5.30E-09
rs2638315	12	56865056	C(G)	0.182	0.041	0.005	1.20E-15
rs36181536	12	122528263	C(T)	0.673	-0.020	0.004	1.50E-06
rs10848097	12	130897844	C(A)	0.607	0.019	0.004	4.40E-06
rs1601935	15	58671765	T(G)	0.655	0.023	0.004	7.10E-08
rs9940976	16	72311826	C(A)	0.202	-0.023	0.005	2.80E-06
rs35452938	16	70270907	C(T)	0.114	-0.095	0.006	4.00E-50
rs9930957	16	72149923	T(C)	0.160	0.053	0.005	2.10E-22
rs79327462	16	67958058	A(G)	0.045	-0.045	0.010	2.60E-06
rs2326458	16	84987679	A(C)	0.745	-0.022	0.005	9.30E-07

rs117643180	17	7185779	A(C)	0.026	-0.118	0.013	4.00E-21
rs3848453	17	68362094	C(T)	0.584	0.019	0.004	4.00E-06
rs4801776	19	49304215	T(C)	0.299	-0.032	0.004	2.40E-13
rs10403561	19	33790502	G(A)	0.426	0.018	0.004	4.30E-06
rs429358	19	45411941	C(T)	0.155	-0.026	0.005	3.20E-06
rs12974412	19	14151809	G(A)	0.780	-0.029	0.005	3.30E-09
rs62111417	19	7193981	T(C)	0.713	-0.021	0.004	2.10E-06
rs5747934	22	18915282	T(C)	0.042	-0.057	0.010	8.60E-09

Supplementary Table 6. The characteristics of isoleucine associated SNPs.

SNP	CHR	POS	EA(OA)	EAF	BETA	SE	PVAL
rs11166420	1	100702216	A(T)	0.904	-0.032	0.007	3.10E-06
rs1260326	2	27730940	C(T)	0.604	-0.047	0.004	2.30E-29
rs36216559	2	262335	G(A)	0.350	-0.020	0.004	2.40E-06
rs2422358	2	65231806	T(A)	0.436	0.023	0.004	1.00E-07
rs10184004	2	165508389	T(C)	0.406	-0.030	0.004	4.00E-13
rs3771600	2	159404780	G(C)	0.275	-0.021	0.005	3.30E-06
rs7649917	3	193289549	C(T)	0.227	0.022	0.005	3.60E-06
rs1471740	3	136328270	C(T)	0.740	0.025	0.005	6.10E-08
rs146774705	3	96082587	A(G)	0.030	0.056	0.012	3.30E-06
rs150277164	4	89258580	A(G)	0.014	0.080	0.017	3.00E-06
rs2127864	4	89147993	G(A)	0.175	-0.031	0.005	5.00E-09
rs116431586	4	142523371	T(C)	0.027	-0.067	0.013	1.90E-07
rs10018448	4	89225171	G(A)	0.535	0.067	0.004	4.10E-60
rs7380516	5	135118327	T(A)	0.644	-0.023	0.005	4.30E-06
rs2973444	5	90417593	C(T)	0.086	-0.037	0.007	4.90E-07
rs30351	5	55794632	A(G)	0.744	0.022	0.005	1.70E-06
rs35509506	6	44595179	T(C)	0.135	0.028	0.006	4.20E-06
rs565181303	6	160073565	T(G)	0.203	-0.025	0.005	4.60E-06
rs199607859	6	139835418	T(G)	0.595	-0.020	0.004	2.30E-06
rs1682628	7	95283613	A(G)	0.642	-0.020	0.004	2.50E-06
rs2941456	8	76443463	A(G)	0.198	0.029	0.005	9.10E-09

rs73343569	8	132537295	A(G)	0.010	-0.107	0.022	1.50E-06
rs113821719	10	11824902	C(G)	0.018	0.076	0.016	4.10E-06
rs1924699	10	95565254	T(G)	0.940	0.039	0.009	3.80E-06
rs2616272	11	97448214	A(G)	0.496	-0.020	0.004	6.60E-07
rs2168101	11	8255408	A(C)	0.308	-0.021	0.005	4.90E-06
rs79259321	12	47056356	A(C)	0.072	0.039	0.008	5.90E-07
rs7302925	12	56861458	G(A)	0.801	-0.041	0.005	3.60E-16
rs941952	14	104559623	C(T)	0.719	0.023	0.005	7.50E-07
rs111423330	15	62418810	C(T)	0.327	-0.021	0.004	3.40E-06
rs7201861	16	79532675	C(G)	0.711	0.021	0.004	2.70E-06
rs117164273	16	68292616	A(G)	0.045	-0.051	0.010	1.70E-07
rs2326458	16	84987679	A(C)	0.745	-0.024	0.005	3.80E-07
rs12325419	16	70368909	A(G)	0.115	-0.074	0.006	1.10E-31
rs117643180	17	7185779	A(C)	0.026	-0.101	0.013	2.90E-15
rs9899432	17	8204370	C(G)	0.158	-0.030	0.006	8.90E-08
rs1605749	17	68475145	G(A)	0.511	-0.020	0.004	5.70E-07
rs589625	18	524173	C(G)	0.812	0.024	0.005	4.10E-06
rs4805474	19	30250970	G(T)	0.712	0.021	0.004	3.80E-06
rs545587	19	49319664	C(A)	0.515	0.037	0.004	2.30E-19
rs5747934	22	18915282	T(C)	0.042	-0.049	0.010	1.10E-06

Supplementary Table 7. The characteristics of valine associated SNPs.

SNP	CHR	POS	EA(OA)	EAF	BETA	SE	PVAL
rs6427304	1	156156789	G(A)	0.355	0.021	0.004	8.10E-07
rs4660293	1	40028180	G(A)	0.233	0.024	0.005	2.60E-07
rs41294810	1	55063953	G(T)	0.261	-0.022	0.005	1.30E-06
rs2943652	2	227108446	T(C)	0.645	0.024	0.004	1.20E-08
rs2422358	2	65231806	T(A)	0.436	0.061	0.004	2.90E-47
rs1128249	2	165528624	T(G)	0.392	-0.035	0.004	1.50E-17
rs1260326	2	27730940	C(T)	0.604	-0.051	0.004	6.50E-36
rs13402475	2	3639909	G(C)	0.816	0.026	0.005	8.00E-07
rs10469763	2	211510528	C(T)	0.451	0.019	0.004	2.80E-06
rs7649917	3	193289549	C(T)	0.227	0.023	0.005	1.70E-06
rs62271373	3	150066540	A(T)	0.060	0.040	0.009	3.20E-06
rs7650482	3	12841804	G(A)	0.651	-0.021	0.004	1.10E-06
rs34894639	3	135798658	T(C)	0.232	-0.028	0.005	6.00E-09
rs13100474	3	121878781	A(G)	0.206	0.024	0.005	1.60E-06
rs7659144	4	3098321	G(C)	0.344	-0.020	0.004	4.10E-06
rs2127864	4	89147993	G(A)	0.175	-0.048	0.005	1.30E-19
rs10018448	4	89225171	G(A)	0.535	0.105	0.004	2.30E-148
rs71624137	5	55859146	C(T)	0.124	0.031	0.006	5.20E-07
rs3112484	5	90401221	A(G)	0.096	-0.033	0.007	1.30E-06
rs61587941	5	55814890	A(G)	0.051	-0.050	0.009	3.40E-08
rs74823953	5	150679610	T(C)	0.013	-0.088	0.018	8.00E-07

rs35509506	6	44595179	T(C)	0.135	0.028	0.006	3.90E-06
rs75041599	6	143782486	A(G)	0.017	0.077	0.016	2.20E-06
rs6941263	6	109162094	A(T)	0.188	-0.029	0.005	3.40E-08
rs71562509	6	139835423	T(G)	0.593	-0.019	0.004	3.00E-06
rs12174589	6	161099044	T(A)	0.207	0.025	0.005	6.50E-07
rs56001710	7	25983400	T(A)	0.581	-0.020	0.004	3.00E-06
rs6953476	7	98921600	C(G)	0.128	-0.030	0.006	4.90E-07
rs1682628	7	95283613	A(G)	0.642	-0.020	0.004	2.10E-06
rs2977929	8	76454025	T(C)	0.197	0.027	0.005	1.20E-07
rs1333051	9	22136489	T(A)	0.136	-0.028	0.006	1.80E-06
rs1438506	10	133108620	A(T)	0.353	-0.019	0.004	4.50E-06
rs2394861	10	74078627	C(T)	0.658	0.022	0.004	1.10E-07
rs2497349	10	94521565	T(G)	0.308	0.021	0.004	8.20E-07
rs17096421	10	88820592	T(A)	0.056	0.063	0.009	1.40E-12
rs911268	11	30809653	C(T)	0.331	0.021	0.004	1.80E-06
rs2168101	11	8255408	A(C)	0.308	-0.021	0.004	1.90E-06
rs2616272	11	97448214	A(G)	0.496	-0.019	0.004	1.40E-06
rs140900031	11	18489528	A(G)	0.479	0.019	0.004	4.10E-06
rs2638315	12	56865056	C(G)	0.182	0.043	0.005	2.20E-16
rs579526	12	373120	C(T)	0.231	0.022	0.005	2.40E-06
rs76895963	12	4384844	G(T)	0.021	-0.075	0.016	1.70E-06
rs12318565	12	92736142	C(A)	0.348	0.022	0.005	7.30E-07
rs36181536	12	122528263	C(T)	0.674	-0.032	0.004	1.30E-13

rs11045819	12	21329813	A(C)	0.161	0.025	0.005	3.50E-06
rs1028883	13	74108587	G(T)	0.572	-0.020	0.004	1.20E-06
rs28435138	13	20694866	A(G)	0.327	-0.021	0.004	1.60E-06
rs2274815	14	102718052	A(G)	0.205	0.028	0.005	1.80E-08
rs75663391	15	63910433	T(G)	0.680	-0.021	0.004	1.60E-06
rs6501179	16	3610642	C(T)	0.440	0.019	0.004	3.90E-06
rs17817288	16	53807764	G(A)	0.485	0.021	0.004	2.20E-07
rs117164273	16	68292616	A(G)	0.045	-0.047	0.010	1.40E-06
rs12325419	16	70368909	A(G)	0.115	-0.090	0.006	1.10E-46
rs9941239	16	84987947	G(A)	0.756	-0.024	0.005	5.50E-07
rs8071084	17	79634162	G(T)	0.162	-0.032	0.005	3.10E-09
rs12936169	17	40824823	A(G)	0.286	-0.022	0.004	1.20E-06
rs222856	17	7110413	A(G)	0.020	-0.071	0.015	1.30E-06
rs62088469	17	73519589	C(T)	0.166	-0.026	0.005	2.10E-06
rs117643180	17	7185779	A(C)	0.026	-0.175	0.013	6.30E-43
rs12974412	19	14151809	G(A)	0.780	-0.027	0.005	4.80E-08
rs35230038	19	49300431	A(G)	0.045	-0.098	0.010	1.10E-23
rs66921136	19	7197880	C(T)	0.712	-0.024	0.004	7.10E-08
rs117048185	19	49309776	C(G)	0.017	0.190	0.015	8.70E-35
rs837616	19	49365588	G(A)	0.292	-0.025	0.004	1.60E-08
rs67555182	21	26686210	A(G)	0.106	0.032	0.007	1.30E-06
rs3970551	22	18906839	G(A)	0.114	-0.034	0.006	1.10E-07
rs2238732	22	18915347	T(C)	0.042	-0.062	0.010	5.80E-10

Supplementary Table 8. The characteristics of BCAA associated SNPs.

SNP	CHR	POS	EA(OA)	EAF	BETA	SE	PVAL
rs4660293	1	40028180	G(A)	0.233	0.023	0.005	1.30E-06
rs145815601	1	155027082	T(G)	0.037	-0.054	0.011	6.60E-07
rs2943652	2	227108446	T(C)	0.645	0.023	0.004	2.20E-08
rs1128249	2	165528624	T(G)	0.392	-0.035	0.004	2.00E-17
rs1260326	2	27730940	C(T)	0.604	-0.051	0.004	2.70E-36
rs13402475	2	3639909	G(C)	0.816	0.026	0.005	9.10E-07
rs2422358	2	65231806	T(A)	0.436	0.044	0.004	7.70E-26
rs34894639	3	135798658	T(C)	0.232	-0.028	0.005	3.50E-09
rs13100474	3	121878781	A(G)	0.206	0.025	0.005	5.70E-07
rs7649917	3	193289549	C(T)	0.227	0.024	0.005	6.50E-07
rs116431586	4	142523371	T(C)	0.027	-0.059	0.013	2.80E-06
rs55962025	4	3112109	C(A)	0.355	-0.020	0.004	2.30E-06
rs2127864	4	89147993	G(A)	0.175	-0.042	0.005	1.40E-15
rs10018448	4	89225171	G(A)	0.535	0.096	0.004	7.29E-128
rs11948950	5	55876283	A(G)	0.105	0.032	0.007	1.70E-06
rs74823953	5	150679610	T(C)	0.013	-0.084	0.018	2.10E-06
rs61587941	5	55814890	A(G)	0.051	-0.048	0.009	9.30E-08
rs3112484	5	90401221	A(G)	0.096	-0.035	0.007	4.10E-07
rs6941263	6	109162094	A(T)	0.188	-0.027	0.005	1.80E-07
rs12174589	6	161099044	T(A)	0.207	0.023	0.005	3.30E-06
rs35509506	6	44595179	T(C)	0.135	0.027	0.006	3.90E-06

rs199607859	6	139835418	T(G)	0.595	-0.020	0.004	1.10E-06
rs10255399	7	98970124	C(G)	0.128	-0.029	0.006	1.20E-06
rs56001710	7	25983400	T(A)	0.581	-0.019	0.004	4.00E-06
rs2977929	8	76454025	T(C)	0.197	0.029	0.005	1.10E-08
rs9416015	10	74090617	T(G)	0.620	0.019	0.004	2.40E-06
rs1438506	10	133108620	A(T)	0.353	-0.020	0.004	2.70E-06
rs12219397	10	5146578	C(T)	0.218	0.023	0.005	2.20E-06
rs17096421	10	88820592	T(A)	0.056	0.054	0.009	9.70E-10
rs2021807	11	30806998	C(T)	0.331	0.020	0.004	2.70E-06
rs2616272	11	97448214	A(G)	0.496	-0.019	0.004	3.00E-06
rs2168101	11	8255408	A(C)	0.308	-0.022	0.004	1.20E-06
rs79297632	12	47181325	C(G)	0.068	0.038	0.008	1.50E-06
rs36181536	12	122528263	C(T)	0.674	-0.026	0.004	7.00E-10
rs579526	12	373120	C(T)	0.231	0.022	0.005	3.00E-06
rs12318565	12	92736142	C(A)	0.348	0.022	0.005	1.30E-06
rs4766578	12	111904371	A(T)	0.503	-0.020	0.004	9.10E-07
rs1564369	12	21335290	G(T)	0.178	0.025	0.005	1.70E-06
rs2638315	12	56865056	C(G)	0.182	0.044	0.005	1.80E-17
rs28435138	13	20694866	A(G)	0.327	-0.020	0.004	3.10E-06
rs2274815	14	102718052	A(G)	0.205	0.025	0.005	7.40E-07
rs12325419	16	70368909	A(G)	0.115	-0.092	0.006	1.00E-48
rs117164273	16	68292616	A(G)	0.045	-0.050	0.010	1.60E-07
rs2326458	16	84987679	A(C)	0.745	-0.024	0.005	2.60E-07

rs28731397	16	72097279	C(T)	0.326	0.023	0.004	1.40E-07
rs17817288	16	53807764	G(A)	0.485	0.019	0.004	1.90E-06
rs1605749	17	68475145	G(A)	0.511	-0.019	0.004	2.40E-06
rs117643180	17	7185779	A(C)	0.026	-0.149	0.013	3.80E-32
rs8071084	17	79634162	G(T)	0.162	-0.027	0.005	6.10E-07
rs589625	18	524173	C(G)	0.812	0.024	0.005	3.60E-06
rs12974412	19	14151809	G(A)	0.780	-0.028	0.005	1.80E-08
rs146363641	19	49706439	T(C)	0.019	0.072	0.016	4.00E-06
rs66921136	19	7197880	C(T)	0.712	-0.023	0.004	1.80E-07
rs4801776	19	49304215	T(C)	0.299	-0.039	0.004	1.30E-18
rs3970551	22	18906839	G(A)	0.114	-0.030	0.006	3.50E-06
rs5747934	22	18915282	T(C)	0.042	-0.061	0.010	1.20E-09

Supplementary Table 9. Comparison of four different statistical methods for MR analysis evaluating the causal association of female shift work, BCAA, PCOS and sex hormones.

Exposure-Outcome	Methods	nSNP	β/OR 95%CI	PVAL
Women shift work-PCOS	MR Egger	206	1.02(0.97-1.07)	5.56E-01
	Weighted median	206	1.03(0.96-1.11)	4.40E-01
	Inverse variance weighted	206	1.06(1.01-1.1)	**1.31E-02**
	Weighted mode	206	1.04(0.97-1.11)	2.50E-01
Women shift work-TT	MR Egger	345	0.00(-0.02-0.03)	6.58E-01
	Weighted median	345	-0.01(-0.03-0.02)	6.58E-01
	Inverse variance weighted	345	0.00(-0.02-0.01)	7.07E-01
	Weighted mode	345	-0.07(-0.16-0.02)	1.28E-01
Women shift work-Bio-T	MR Egger	345	0.04(0.02-0.06)	**4.54E-05**
	Weighted median	345	0.02(-0.01-0.04)	1.79E-01
	Inverse variance weighted	345	-0.02(-0.04-0.00)	7.22E-02
	Weighted mode	345	0.08(0.00-0.17)	5.97E-02
	MR-PRESSO	345	-0.02(-0.04-0.00)	5.36E-02
Women shift work-SHBG	MR Egger	345	-0.03(-0.04--0.02)	**3.27E-06**
	Weighted median	345	-0.01(-0.02-0.01)	4.62E-01
	Inverse variance weighted	345	0.00(-0.01-0.013)	5.11E-01
	Weighted mode	345	-0.01(-0.06-0.03)	6.05E-01
Women shift work-E_2	MR Egger	345	0.00(-0.00-0.01)	3.97E-01
	Weighted median	345	-0.01(-0.02-0.00)	**2.10E-02**

	Inverse variance weighted	345	0(-0.005-0.005)	9.79E-01
	Weighted mode	345	-0.02(-0.04-0.01)	1.89E-01
Women shift work-AMH	MR Egger	52	-0.15(-0.75-0.46)	6.35E-01
	Weighted median	52	-0.11(-0.57-0.36)	6.53E-01
	Inverse variance weighted	52	-0.15(-0.50-0.19)	3.90E-01
	Weighted mode	52	-0.09(-1.00-0.82)	8.49E-01
Leu-PCOS	MR Egger	44	0.99(0.84-1.18)	9.45E-01
	Weighted median	44	1.08(0.93-1.25)	3.35E-01
	Inverse variance weighted	44	1.12(1.01-1.24)	**2.68E-02**
	Weighted mode	44	1.01(0.85-1.19)	9.39E-01
Leu-TT	MR Egger	49	0.07(-0.10-0.25)	4.21E-01
	Weighted median	49	0.05(0.01-0.10)	**2.66E-02**
	Inverse variance weighted	49	-0.07(-0.16-0.02)	1.19E-01
	Weighted mode	49	0.06(0.02-0.10)	**1.05E-02**
	MR-PRESSO	49	0.01(-0.05-0.06)	8.60E-01
Leu-Bio-T	MR Egger	49	0.01(-0.15-0.18)	8.85E-01
	Weighted median	49	0.07(0.02-0.12)	**4.59E-03**
	Inverse variance weighted	49	0.12(0.04-0.20)	**5.25E-03**
	Weighted mode	49	0.05(0.01-0.10)	**2.08E-02**
	MR-PRESSO	49	0.09(0.04-0.13)	**4.78E-04**
BioT-Leu	MR Egger	157	0.05(-0.02-0.12)	1.86E-01
	Weighted median	157	0.07(0.02-0.12)	**4.57E-03**

	Inverse variance weighted	157	0.06(0.02-0.10)	**6.71E-03**
	Weighted mode	157	0.08(0.03-0.13)	**2.55E-03**
	MR-PRESSO	157	0.05(0.01-0.09)	1.07E-02
Leu-SHBG	MR Egger	49	0.03(-0.17-0.22)	7.90E-01
	Weighted median	49	-0.04(-0.07--0.01)	**6.97E-03**
	Inverse variance weighted	49	-0.16(-0.26--0.06)	**1.96E-03**
	Weighted mode	49	-0.03(-0.06--0.01)	**1.18E-02**
	MR-PRESSO	49	-0.10(-0.14--0.06)	**2.20E-05**
SHBG-Leu	MR Egger	315	-0.12(-0.20--0.04)	**2.26E-03**
	Weighted median	315	-0.10(-0.16--0.04)	**1.15E-03**
	Inverse variance weighted	315	-0.20(-0.25--0.14)	**4.52E-13**
	Weighted mode	315	-0.10(-0.15--0.04)	**4.15E-04**
	MR-PRESSO		-0.17(-0.22--0.13)	**6.61E-13**
Leu-E_2	MR Egger	50	0.00(-0.03-0.03)	9.80E-01
	Weighted median	50	0.00(-0.02-0.01)	7.04E-01
	Inverse variance weighted	50	-0.01(-0.03-0.00)	1.66E-01
	Weighted mode	50	0.01(-0.01-0.02)	4.72E-01
	MR-PRESSO	50	-0.01(-0.02-0.00)	2.06E-01
Leu-AMH	MR Egger	43	0.36(-0.17-0.88)	1.96E-01
	Weighted median	43	0.13(-0.26-0.52)	5.10E-01
	Inverse variance weighted	43	0.13(-0.12-0.38)	3.19E-01
	Weighted mode	43	0.17(-0.24-0.58)	4.30E-01

Val-PCOS	MR Egger	54	1.06(0.85-1.33)	5.87E-01
	Weighted median	54	0.98(0.84-1.14)	8.18E-01
	Inverse variance weighted	54	0.98(0.88-1.09)	7.58E-01
	Weighted mode	54	1.04(0.84-1.30)	7.01E-01
Val-TT	MR Egger	66	0.00(-0.12-0.12)	9.80E-01
	Weighted median	66	0.04(-0.00-0.08)	5.35E-02
	Inverse variance weighted	66	-0.02(-0.09-0.05)	5.76E-01
	Weighted mode	66	0.04(0.00-0.08)	5.27E-02
	MR-PRESSO	66	0.02(-0.01-0.06)	1.88E-01
Val-Bio-T	MR Egger	66	0.06(-0.05-0.17)	3.16E-01
	Weighted median	66	0.06(0.02-0.10)	**2.60E-03**
	Inverse variance weighted	66	0.12(0.06-0.18)	**1.80E-04**
	Weighted mode	66	0.06(0.01-0.10)	**1.06E-02**
	MR-PRESSO	66	0.10(0.07-0.14)	**1.40E-06**
BioT-Val	MR Egger	157	0.05(-0.04-0.13)	2.72E-01
	Weighted median	157	0.07(0.02-0.12)	**3.99E-03**
	Inverse variance weighted	157	0.07(0.02-0.12)	**3.78E-03**
	Weighted mode	157	0.08(0.03-0.13)	**3.72E-03**
	MR-PRESSO	157	0.06(0.02-0.10)	**3.85E-03**
Val-SHBG	MR Egger	66	-0.06(-0.17-0.05)	3.20E-01
	Weighted median	66	-0.04(-0.06--0.01)	**4.67E-03**
	Inverse variance weighted	66	-0.14(-0.2--0.08)	**1.55E-05**

	Weighted mode	66	-0.04(-0.06--0.02)	**4.27E-04**
	MR-PRESSO	66	-0.11(-0.14--0.08)	**5.19E-09**
SHBG-Val	MR Egger	315	-0.14(-0.22--0.06)	**1.15E-03**
	Weighted median	315	-0.07(-0.13--0.01)	**1.51E-02**
	Inverse variance weighted	315	-0.24(-0.29--0.18)	**6.74E-16**
	Weighted mode	315	-0.08(-0.13--0.03)	**3.21E-03**
	MR-PRESSO	315	-0.28(-0.34--0.22)	**2.81E-17**
Val-E_2	MR Egger	66	0.00(-0.02-0.02)	9.96E-01
	Weighted median	66	0.01(0.00-0.02)	2.34E-01
	Inverse variance weighted	66	-0.01(-0.02-0.01)	3.46E-01
	Weighted mode	66	0.01(-0.01-0.02)	2.92E-01
	MR-PRESSO	66	0.00(-0.01-0.01)	9.94E-01
Val-AMH	MR Egger	57	0.11(-0.24-0.46)	5.41E-01
	Weighted median	57	0.08(-0.25-0.40)	6.44E-01
	Inverse variance weighted	57	0.08(-0.11-0.27)	4.30E-01
	Weighted mode	57	0.04(-0.28-0.36)	7.85E-01
Ile-PCOS	MR Egger	34	1.17(0.83-1.64)	3.77E-01
	Weighted median	34	1.08(0.90-1.29)	4.26E-01
	Inverse variance weighted	34	1.12(0.98-1.28)	1.09E-01
	Weighted mode	34	0.96(0.71-1.30)	8.04E-01
lle-TT	MR Egger	40	-0.01(-0.22-0.20)	9.52E-01
	Weighted median	40	0.06(0.01-0.12)	**2.91E-02**

	Inverse variance weighted	40	-0.07(-0.16-0.02)	1.44E-01
	Weighted mode	40	0.06(0.00-0.13)	5.44E-02
	MR-PRESSO	40	0.02(-0.04-0.07)	5.60E-01
lle-Bio-T	MR Egger	40	0.02(-0.15-0.19)	8.05E-01
	Weighted median	40	0.08(0.03-0.14)	**2.23E-03**
	Inverse variance weighted	40	0.11(0.04-0.19)	**2.82E-03**
	Weighted mode	40	0.07(0.01-0.13)	**3.01E-02**
	MR-PRESSO	40	0.07(0.03-0.12)	**1.09E-03**
BioT-lle	MR Egger	157	0.04(-0.03-0.12)	2.57E-01
	Weighted median	157	0.07(0.02-0.12)	**1.19E-02**
	Inverse variance weighted	157	0.05(0.00-0.09)	**3.59E-02**
	Weighted mode	157	0.08(0.02-0.14)	**7.06E-03**
	MR-PRESSO	157	0.05(0.01-0.08)	**8.21E-03**
lle-SHBG	MR Egger	40	-0.04(-0.24-0.17)	7.28E-01
	Weighted median	40	-0.05(-0.08--0.02)	**1.06E-03**
	Inverse variance weighted	40	-0.16(-0.25--0.07)	**5.24E-04**
	Weighted mode	40	-0.04(-0.08--0.01)	**1.53E-02**
	MR-PRESSO	40	-0.09(-0.12--0.05)	**3.51E-05**
SHBG-Ile	MR Egger	315	-0.12(-0.2--0.05)	**1.80E-03**
	Weighted median	315	-0.09(-0.15--0.04)	**1.52E-03**
	Inverse variance weighted	315	-0.18(-0.23--0.13)	**2.43E-11**
	Weighted mode	315	-0.09(-0.14--0.04)	**7.57E-04**

	MR-PRESSO	315	-0.16(-0.21--0.12)	**2.25E-11**
lle-E_2	MR Egger	40	0.00(-0.03-0.04)	8.08E-01
	Weighted median	40	-0.01(-0.02-0.01)	4.50E-01
	Inverse variance weighted	40	-0.02(-0.03--0.00)	**3.83E-02**
	Weighted mode	40	0.00(-0.02-0.02)	7.20E-01
lle-AMH	MR Egger	36	0.11(-0.48-0.70)	7.22E-01
	Weighted median	36	0.14(-0.26-0.54)	4.83E-01
	Inverse variance weighted	36	0.24(-0.01-0.49)	6.42E-02
	Weighted mode	36	0.20(-0.30-0.70)	4.32E-01
BCAA-PCOS	MR Egger	42	1.18(0.89-1.56)	2.47E-01
	Weighted median	42	1.13(0.95-1.34)	1.76E-01
	Inverse variance weighted	42	1.07(0.94-1.21)	3.15E-01
	Weighted mode	42	1.12(0.92-1.37)	2.69E-01
BCAA-TT	MR Egger	54	0.00(-0.16-0.16)	9.61E-01
	Weighted median	54	0.05(0.00-0.10)	**3.75E-02**
	Inverse variance weighted	54	-0.04(-0.12-0.04)	3.63E-01
	Weighted mode	54	0.05(0.01-0.09)	**3.07E-02**
	MR-PRESSO	54	-0.00(-0.06-0.05)	9.06E-01
BCAA-Bio-T	MR Egger	54	0.02(-0.11-0.16)	7.38E-01
	Weighted median	54	0.07(0.02-0.11)	**5.45E-03**
	Inverse variance weighted	54	0.12(0.05-0.19)	**9.65E-04**
	Weighted mode	54	0.06(0.01-0.10)	**1.77E-02**

	MR-PRESSO	54	0.11(0.07-0.15)	**1.02E-05**
BioT-BCAA	MR Egger	157	0.05(-0.03-0.13)	2.34E-01
	Weighted median	157	0.08(0.03-0.13)	**2.64E-03**
	Inverse variance weighted	157	0.06(0.02-0.11)	**5.63E-03**
	Weighted mode	157	0.09(0.04-0.14)	**9.00E-04**
	MR-PRESSO	157	0.06(0.02-0.09)	**6.96E-03**
BCAA-SHBG	MR Egger	54	-0.04(-0.18-0.10)	6.05E-01
	Weighted median	54	-0.03(-0.06--0.01)	**8.70E-03**
	Inverse variance weighted	54	-0.14(-0.21--0.07)	**2.09E-04**
	Weighted mode	54	-0.03(-0.05--0.01)	**4.49E-03**
	MR-PRESSO	54	-0.12(-0.16--0.08)	**2.14E-06**
SHBG-BCAA	MR Egger	315	-0.13(-0.22--0.05)	**1.14E-03**
	Weighted median	315	-0.09(-0.15--0.03)	**2.58E-03**
	Inverse variance weighted	315	-0.22(-0.28--0.17)	**6.83E-15**
	Weighted mode	315	-0.09(-0.14--0.04)	**4.26E-04**
	MR-PRESSO	315	-0.26(-0.33--0.2)	**4.69E-16**
BCAA-E_2	MR Egger	54	-0.01(-0.03-0.02)	6.37E-01
	Weighted median	54	0.00(-0.01-0.01)	9.10E-01
	Inverse variance weighted	54	-0.01(-0.02-0.00)	1.23E-01
	Weighted mode	54	0.00(-0.01-0.02)	5.51E-01
BCAA-AMH	MR Egger	46	0.25(-0.17-0.66)	2.50E-01
	Weighted median	46	0.08(-0.25-0.42)	6.24E-01

	Inverse variance weighted	46	0.11(-0.10-0.32)	2.88E-01
	Weighted mode	46	0.03(-0.33-0.39)	8.64E-01
Women shift work-Val	MR Egger	177	0.03(-0.02-0.07)	1.98E-01
	Weighted median	177	0.05(0.00-0.10)	**3.68E-02**
	Inverse variance weighted	177	0.09(0.05-0.12)	**1.03E-06**
	Weighted mode	177	0.03(-0.11-0.16)	7.12E-01
Women shift work-lle	MR Egger	177	-0.01(-0.05-0.04)	7.96E-01
	Weighted median	177	0.05(0.00-0.10)	5.10E-02
	Inverse variance weighted	177	0.08(0.04-0.12)	**8.12E-05**
	Weighted mode	177	-0.02(-0.18-0.13)	7.61E-01
Women shift work-BCAA	MR Egger	177	0.02(-0.02-0.06)	3.84E-01
	Weighted median	177	0.05(0.01-0.10)	**3.03E-02**
	Inverse variance weighted	177	0.07(0.04-0.11)	**4.20E-05**
	Weighted mode	177	0.02(-0.12-0.16)	7.66E-01
Bold	P-value <0.05			

Supplementary Table10. Heterogeneity test for each group.

Exposure-Outcome	Methods	Q	Q_df	QVAL
Women shift work-PCOS	MR-Egger	164.120	204	9.82E-01
		175.350	205	9.34E-01
Women shift work-TT	MR-Egger	318.960	343	8.20E-01
		320.890	344	8.10E-01
Women shift work-Bio-T	MR-Egger	425.760	343	**1.53E-03**
		569.450	344	**2.25E-13**
Women shift work-SHBG	MR-Egger	336.040	343	5.96E-01
		432.100	344	**8.64E-04**
Women shift work-E_2	MR-Egger	346.150	343	4.42E-01
		348.700	344	4.19E-01
Women shift work-AMH	MR-Egger	23.530	50	9.99E-01
		23.530	51	1.00E+00
Women shift work-lle	MR-Egger	174.020	175	5.07E-01
		216.280	176	**2.08E-02**
Women shift work-Leu	MR-Egger	163.320	175	7.27E-01
		167.430	176	6.66E-01
Women shift work-Val	MR-Egger	144.030	175	9.58E-01
		164.860	176	7.16E-01
Women shift work-BCAA	MR-Egger	155.770	175	8.49E-01
		173.320	176	5.43E-01

Ile-PCOS	MR-Egger	40.301	32	1.49E-01
		40.396	33	1.76E-01
Ile-TT	MR-Egger	276.180	38	**6.38E-38**
		279.119	39	**4.86E-38**
Ile-Bio-T	MR-Egger	215.545	38	**1.13E-26**
		223.476	39	**9.98E-28**
Bio-T-Ile	MR-Egger	349.972	155	**4.19E-17**
		349.991	156	**6.31E-17**
Ile-SHBG	MR-Egger	991.123	38	**3.18E-183**
		1036.054	39	**6.49E-192**
SHBG-Ile	MR-Egger	808.689	313	**1.51E-45**
		819.499	314	**8.60E-47**
Ile-E_2	MR-Egger	77.659	38	**1.55E-04**
		81.045	39	**8.94E-05**
Ile-AMH	MR-Egger	27.357	34	7.83E-01
		27.597	35	8.09E-01
Leu-PCOS	MR-Egger	46.019	42	3.09E-01
		49.137	43	2.41E-01
PCOS-Leu	MR-Egger	31.850	96	1.00E+00
		31.921	97	1.00E+00
Leu-TT	MR-Egger	421.789	47	**1.05E-61**
		452.658	48	**3.16E-67**
Leu-Bio-T	MR-Egger	462.708	47	**1.08E-69**

		483.590	48	**2.75E-73**
Bio-T-Leu	MR-Egger	351.424	155	**2.78E-17**
	IVW	351.621	156	**3.98E-17**
Leu-SHBG	MR-Egger	1984.449	47	0.00E+00
		2181.065	48	0.00E+00
SHBG-Leu	MR-Egger	879.335	313	**2.97E-55**
		899.997	314	**6.02E-58**
Leu-E_2	MR-Egger	137.439	48	**1.47E-10**
		139.148	49	**1.43E-10**
Leu-AMH	MR-Egger	50.040	41	1.57E-01
		51.158	42	1.57E-01
Val-PCOS	MR-Egger	67.757	52	7.00E-02
		68.574	53	7.37E-02
Val-TT	MR-Egger	481.010	64	**3.23E-65**
		482.191	65	**5.33E-65**
Val-Bio-T	MR-Egger	516.681	64	**5.28E-72**
		529.845	65	**4.60E-74**
Bio-T-Val	MR-Egger	436.017	155	**1.52E-28**
		437.422	156	**1.62E-28**
Val-SHBG	MR-Egger	1641.486	64	**9.95E-301**
		1715.765	65	**1.51E-315**
SHBG-Val	MR-Egger	979.581	313	**9.38E-70**

		1013.233	314	**1.57E-74**
Val-E_2	MR-Egger	145.486	64	**2.77E-08**
		146.345	65	**3.31E-08**
Val-AMH	MR-Egger	60.846	55	2.74E-01
		60.901	56	3.04E-01
BCAA-PCOS	MR-Egger	55.907	40	**4.86E-02**
		56.829	41	5.10E-02
BCAA-TT	MR-Egger	458.424	52	**2.09E-66**
		461.532	53	**1.57E-66**
BCAA-Bio-T	MR-Egger	396.250	52	**1.76E-54**
		415.636	53	**1.02E-57**
Bio-T-BCAA	MR-Egger	409.191	155	**8.17E-25**
		409.852	156	**1.09E-24**
BCAA-SHBG	MR-Egger	1415.336	52	**5.42E-262**
		1487.868	53	**1.81E-276**
SHBG-BCAA	MR-Egger	952.377	313	**9.62E-66**
		979.276	314	**1.85E-69**
BCAA-E_2	MR-Egger	117.363	52	**5.88E-07**
		117.649	53	**8.28E-07**
BCAA-AMH	MR-Egger	46.979	44	3.51E-01
		47.543	45	3.70E-01
Bold	P-value <0.05			

Supplementary Table11. Summary for directional horizontal pleiotropy tests.

Exposure-Outcome	Methods	effect	se	PVAL
Women shift work-PCOS	MR-Egger Intercept	0.007	0.002	**9.59E-04**
		-0.001	0.001	1.66E-01
Women shift work-Bio-T	MR-Egger Intercept	-0.007	0.001	**1.86E-23**
		0.003	0.000	**3.71E-20**
Women shift work-E_2	MR-Egger Intercept	0.000	0.000	1.13E-01
		0.000	0.008	9.91E-01
Women shift work-lle	MR-Egger Intercept	0.006	0.001	**8.06E-10**
		0.002	0.001	**4.40E-02**
Women shift work-Val	MR-Egger Intercept	0.004	0.001	**9.40E-06**
		0.004	0.001	**4.42E-05**
Ile-PCOS	MR-Egger Intercept	-0.001	0.005	7.86E-01
		-0.002	0.004	5.29E-01
Ile-Bio-T	MR-Egger Intercept	0.003	0.003	2.44E-01
		0.000	0.001	9.27E-01
Ile-SHBG	MR-Egger Intercept	-0.004	0.003	1.97E-01
		-0.001	0.001	**4.16E-02**
Ile-E_2	MR-Egger Intercept	-0.001	0.001	2.06E-01
		0.005	0.010	6.27E-01

Leu-PCOS	MR-Egger Intercept	0.005	0.003	9.90E-02
		0.000	0.002	7.91E-01
Leu-TT	MR-Egger Intercept	-0.006	0.003	6.99E-02
		0.004	0.003	1.52E-01
Bio-T-Leu	MR-Egger Intercept	0.000	0.001	7.68E-01
		-0.008	0.003	**3.61E-02**
SHBG-Leu	MR-Egger Intercept	-0.002	0.001	**7.06E-03**
		0.000	0.001	4.44E-01
Leu-AMH	MR-Egger Intercept	-0.009	0.010	3.44E-01
		-0.003	0.004	4.32E-01
Val-TT	MR-Egger Intercept	-0.001	0.002	6.93E-01
		0.003	0.002	2.06E-01
Bio-T-Val	MR-Egger Intercept	0.001	0.001	4.81E-01
		-0.003	0.002	9.36E-02
SHBG-Val	MR-Egger Intercept	-0.002	0.001	**1.16E-03**
		0.000	0.000	5.41E-01
Val-AMH	MR-Egger Intercept	-0.001	0.007	8.25E-01
		-0.004	0.005	4.21E-01
BCAA-TT	MR-Egger Intercept	-0.002	0.003	5.55E-01
		0.004	0.002	1.17E-01
Bio-T-BCAA	MR-Egger Intercept	0.001	0.001	6.18E-01

		-0.004	0.003	1.09E-01
SHBG-BCAA	MR-Egger Intercept	-0.002	0.001	**3.18E-03**
		0.000	0.000	7.23E-01
BCAA-AMH	MR-Egger Intercept	-0.006	0.008	4.71E-01
Bold	P-value <0.05			
